# High-content phenotyping of Parkinson's disease patient stem cell-derived midbrain dopaminergic neurons using machine learning classification

**DOI:** 10.1016/j.stemcr.2022.09.001

**Published:** 2022-09-29

**Authors:** Aurore Vuidel, Loïc Cousin, Beatrice Weykopf, Simone Haupt, Zahra Hanifehlou, Nicolas Wiest-Daesslé, Michaela Segschneider, Joohyun Lee, Yong-Jun Kwon, Michael Peitz, Arnaud Ogier, Laurent Brino, Oliver Brüstle, Peter Sommer, Johannes H. Wilbertz

**Affiliations:** 1 Ksilink, Strasbourg, France; 2 Institute of Reconstructive Neurobiology, University of Bonn Medical Faculty & University Hospital Bonn, Bonn, Germany; 3 LIFE & BRAIN GmbH, Bonn, Germany

**Keywords:** Parkinson's disease, LRRK2, SNCA, iPSC, disease modeling, machine learning

## Abstract

Combining multiple Parkinson's disease (PD) relevant cellular phenotypes might increase the accuracy of midbrain dopaminergic neuron (mDAN) *in vitro* models. We differentiated patient-derived induced pluripotent stem cells (iPSCs) with a LRRK2 G2019S mutation, isogenic control, and genetically unrelated iPSCs into mDANs. Using automated fluorescence microscopy in 384-well-plate format, we identified elevated levels of α-synuclein (αSyn) and serine 129 phosphorylation, reduced dendritic complexity, and mitochondrial dysfunction. Next, we measured additional image-based phenotypes and used machine learning (ML) to accurately classify mDANs according to their genotype. Additionally, we show that chemical compound treatments, targeting LRRK2 kinase activity or αSyn levels, are detectable when using ML classification based on multiple image-based phenotypes. We validated our approach using a second isogenic patient-derived *SNCA* gene triplication mDAN model which overexpresses αSyn. This phenotyping and classification strategy improves the practical exploitability of mDANs for disease modeling and the identification of novel LRRK2-associated drug targets.

## Highlights

• Parkinson’s disease phenotypes are present in two midbrain neuronal models
•Assay automation and adaption to 384-well plates
•Machine learning classifies neuronal genotypes based on image-extracted features
•Machine learning identifies drug-treated neurons based on image-extracted features

## Graphical abstract

**Figure:**
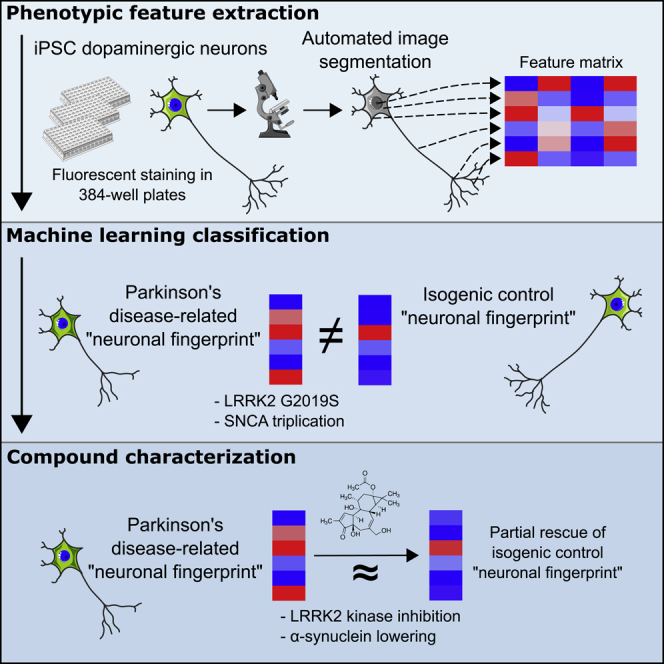

## Teaser

In this article, Wilbertz and colleagues show that machine learning based on phenotypic features can distinguish Parkinson’s disease patient stem cell-derived dopaminergic neurons from isogenic controls. LRRK2 G2019S or SNCA triplication neurons were fluorescently stained, and quantitative phenotypic features were extracted. The resulting phenotypic profiles allowed classification algorithms to identify different genotypes and the detection of chemical compound effects.

## Introduction

Parkinson's disease (PD) is a heterogeneous movement disorder with a combination of motor and non-motor features caused by environmental and genetic risk factors or mutations in specific genes. Pathological characteristics of PD include the progressive loss of midbrain dopaminergic neurons (mDANs) and often the appearance of Lewy bodies, cytoplasmic inclusions containing aggregated α-synuclein (αSyn) protein (Blesa et al., 2022; Poewe et al., 2017).

Mutations in the leucine-rich repeat kinase 2 gene (*LRRK2*) have been associated with PD. The glycine to serine substitution at position 2019 (G2019S) in the LRRK2 kinase domain increases its activity and is assumed to be one reason for mDAN loss (Smith et al., 2006; West et al., 2005; Weykopf et al., 2019). One hypothesis is that LRRK2 G2019S causes defects in mitochondrial biology. Increased autophagy markers, but also PINK1/Parkin-, and Miro1-related defects support the idea that specifically mitophagy-linked processes are disturbed in LRRK2 G2019S neurons (Bonello et al., 2019; Hsieh et al., 2016; Schwab et al., 2017). Additionally, LRRK2 G2019S could induce mDAN loss by increasing the levels of phosphorylated αSyn, leading to its aggregation, since LRRK2 kinase inhibition can prevent phosphorylated αSyn from forming protein inclusions (Daher et al., 2014; Longo et al., 2017; Obergasteiger et al., 2020; Volpicelli-Daley et al., 2016; Xiong et al., 2017).

An emerging picture of PD is therefore that multiple disease mechanisms act together or can even exacerbate each other. Human patient induced pluripotent stem cell (iPSC)-derived mDANs expressing LRRK2 G2019S constitute a valuable *in vitro* model to understand PD. Despite the apparent value of neuronal models, important challenges remain: individual *in vitro* PD pathological features are often subtle or variable when examined across different differentiation batches or genotypes. Furthermore, single isolated PD phenotypes do not capture the multifactorial complexity of PD. Additionally, and despite their relevance physiologically, iPSC neuronal models are rarely used for PD-related drug discovery due to throughput feasibility concerns based on technical complexity as well as genetic variability (Cobb et al., 2018; Elitt et al., 2018). The goal of this study was therefore to develop a robust methodology able to detect multiple cellular PD-related pathophysiological phenotypes in a physiologically relevant human mDAN model system. We aimed for sufficient sensitivity to detect phenotypic variations based on genetic, but also chemical compound-induced phenotypic changes.

We demonstrate that multiple PD-relevant cellular phenotypes can be detected in microscopic images obtained from human patient LRRK2 G2019S iPSC-derived mDANs in 384-well-plate format. We show that machine learning (ML) can be used to distinctively classify PD from control mDANs based on multiple image-derived phenotypes. Finally, we demonstrate that our multi-phenotype classification approach is sensitive enough to detect different small molecules with a PD-relevant mode of action. Our work outlines a novel strategy to use iPSC-derived mDANs from LRRK2 or SNCA mutation carriers for imaging-based disease modeling by computationally combining multiple disease-relevant phenotypes.

## Results

### LRRK2 G2019S mDANs display multiple hallmarks of PD

iPSC lines from a patient with a confirmed LRRK2 G2019S mutation and an isogenic control line (GS/+ and +/+, respectively) were differentiated into mDANs expressing neuronal markers (TUBB3 and MAP2) and dopaminergic neuron markers, including tyrosine hydroxylase (TH) in combination with expression of FOXA2, while the glial marker glial fibrillary acidic protein was only weakly expressed (Figures S1A and S1B). Immunostaining showed similar percentages of TH- and MAP2-expressing isogenic control +/+ and GS/+ neurons indicating comparable differentiation potentials in both genotypes (Figure S1C).

**Figure 1:**
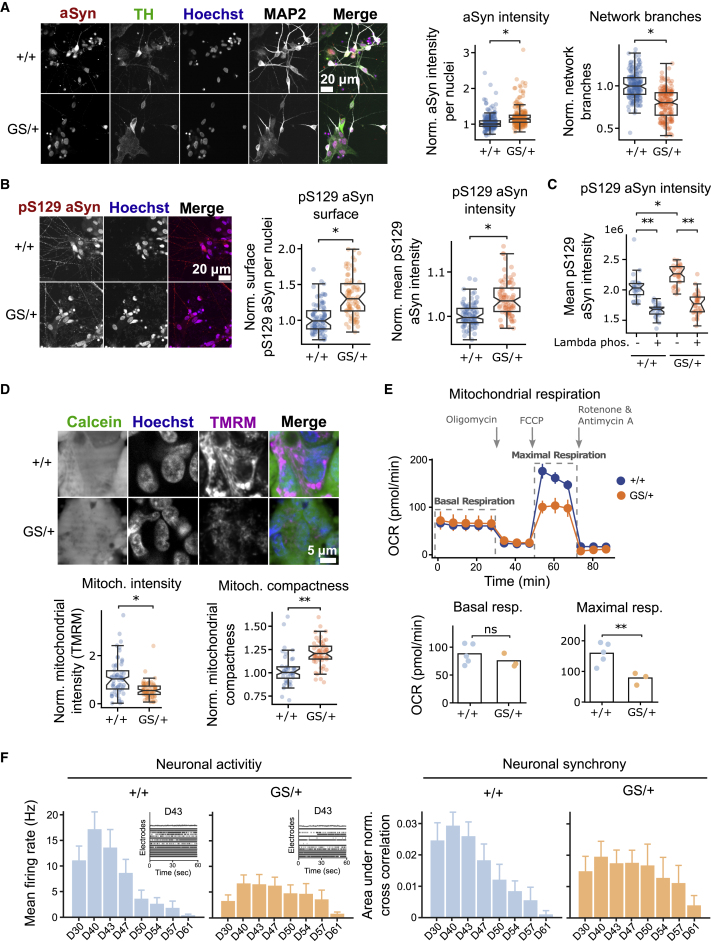
LRRK2 G2019S mDANs overexpress αSyn and display mitochondrial dysfunction (A) iPSC-derived LRRK2 G2019S mDANs were immunostained against αSyn, TH, and MAP2 and αSyn intensity in TH-positive GS/+ neurons as well as a neuronal network complexity was quantified in microscopic images. (B) Immunofluorescence staining against pS129 αSyn, αSyn, and MAP2 and quantification of pS129 αSyn in neurites as well as overall pS129 αSyn fluorescence intensity. (C) mDANs were treated with lambda phosphatase before staining with a pS129 αSyn antibody. (D) Staining with the live cell dye calcein and mitochondrial membrane potential-sensitive dye TMRM and quantification of TMRM intensity and mitochondrial (TMRM) compactness. (E) Assessment of mitochondrial respiration using the Seahorse XF analyzer. Seahorse experiments were performed in triplicate, and means ± SEMs are shown. Imaging experiments shown in panels (B)–(D) were performed at least in duplicate with multiple technical replicates. Each data point represents one well. All data have been median normalized to the respective +/+ condition per plate. Welch’s unequal variances t test was used for significance testing. Notches in boxplots indicate the 95% confidence interval. (F) Multi-electrode array (MEA) recordings of +/+ and GS/+ neuronal population between differentiation D30 and D61. Neuronal activity was determined by measuring action potentials per second on single electrodes. Inserts show sample D43 activity traces from multiple electrodes in one well (left panel). Synchrony between neurons was determined by measuring simultaneous action potentials across multiple electrodes (right panel).

To detect image-based hallmarks of PD, we designed an immunofluorescence-based workflow in 384-well-plate format. Cryopreserved 30-day-old mDANs were seeded in 384-well plates, cultured for 7 days, fixed, and stained. Automated microscopy and image segmentation was used to extract multiple quantitative image features. First, GS/+ and +/+ mDANs were stained with antibodies against αSyn, TH, and MAP2. In the TH-positive GS/+ neuronal population, αSyn levels were increased by 15% (Figure 1A). Western blotting with a different antibody confirmed the increase in αSyn levels across multiple differentiation batches (Figures S1F–S1I). In addition, MAP2 staining indicated that fewer dendritic branches were present in GS/+ neurons (Figure 1A). Staining with a pS129 αSyn antibody showed that the surface area occupied by pS129 αSyn and its fluorescence intensity were increased (Figure 1B). Together, both observations indicate that accumulation as well as increased phosphorylation are occurring. To exclude signal originating from non-phosphorylated forms of αSyn, we treated the fixed cells with lambda phosphatase. Lambda phosphatase treatment strongly reduced pS129 αSyn signal intensity in both GS/+ and +/+ neurons, indicating that pS129 αSyn levels are indeed increased in GS/+ mDANs (Figure 1C).

Staining with the live cell dye calcein and the mitochondrial membrane potential-sensitive dye tetramethylrhodamine (TMRM) indicated that the overall TMRM fluorescence in living cells was decreased by 33% in GS/+ mDANs suggesting that the intactness of the mitochondrial membrane is compromised in GS/+ mDANs. Additionally, mitochondria in GS/+ mDANs were more compact (Figure 1D). We used the mitochondria-targeting toxin rotenone to validate the TMRM staining (Figure S2). Additionally, we measured the oxygen consumption rate. The basal respiration rate did not differ between both genotypes, while the maximal respiration rate after carbonyl cyanide p-(tri-fluromethoxy)phenyl-hydrazone (FCCP) treatment was 2-fold increased in +/+ controls (Figure 1E). To test the mDANs electrophysiological activity, we generated multi-electrode array (MEA) recordings from differentiation D30 to D61. We found that the mean action potential firing rate in GS/+ neurons was less than half compared with +/+ neurons. Additionally, inter-neuronal synchrony was less pronounced in GS/+ neurons, indicating potential synaptic defects (Figure 1F). Taken together, we found that LRRK2 G2019S mDA neurons show multiple hallmarks of PD rendering them useful as a disease model.

### ML classification can distinguish neuronal genotypes based on image-derived cellular features

**Figure 2:**
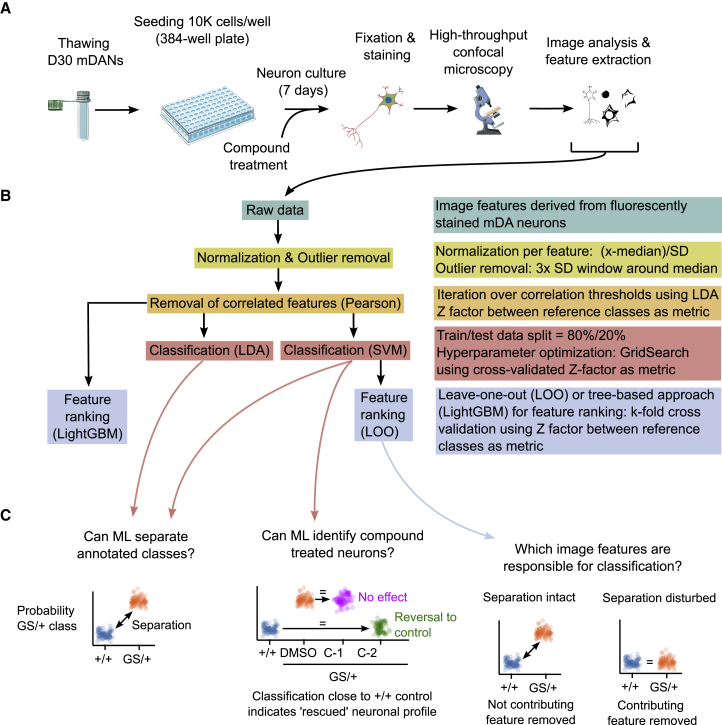
Machine learning (ML) strategy to classify neurons based on image-derived cellular features (A) Schematic depiction of the generation of image-derived cellular feature data. (B) Overview of the data processing steps and ML methodology. (C) Schematic depiction of how ML classification was used to separate different neuronal cell lines (left panel), identify bioactive chemical compounds (middle panel), and how “leave-one-out” analysis can identify the contribution of individual image-derived cellular features to ML classification (right panel).

We hypothesized that the combination of multiple image-based phenotypes would give rise to a “neuronal fingerprint” or “profile” and allow the accurate and robust identification of different cell lines or treatment conditions, thereby making it a useful tool for iPSC-based disease modeling or compound screening. We applied different ML algorithms termed “classifiers” to achieve this task. Specifically, we used linear discriminant analysis (LDA) (Fisher, 1936), support vector machine (SVM) (Cortes and Vapnik, 1995), and light gradient boosting machine (LightGBM) (Ke et al., 2017) algorithms (Figure 2). Quantitative image-derived features were used as input data, and the ML classifiers were trained to separate two classes from each other (Tables S1 and S2). Next, additional classes could be mapped to the pre-trained reference classes. For example, to estimate the effect of a chemical compound treatment, compound-treated cells can be classified in comparison to DMSO-treated mutant and wild-type cells. An increased classification proximity indicates a higher phenotypic similarity (Figure 2C).

**Figure 3:**
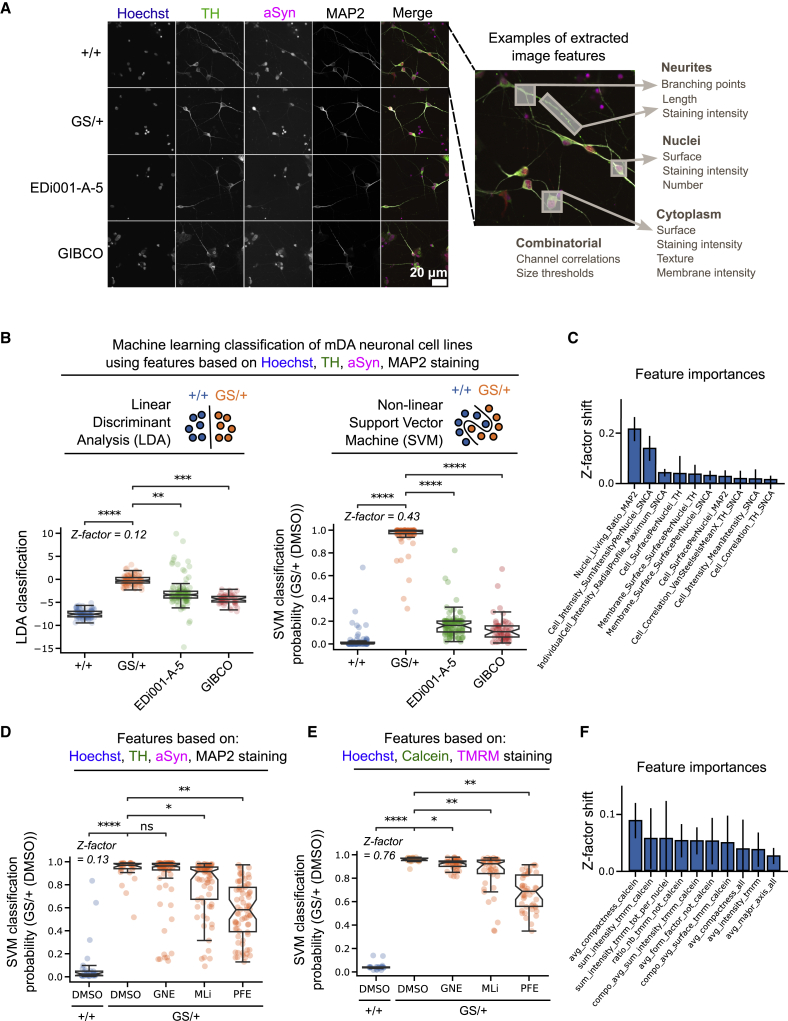
Machine learning (ML) classification can identify genotype-related and chemical compound-induced phenotypic differences based on image-derived cellular features in mDANs (A) Representative images of neurons stained with Hoechst and antibodies against TH, αSyn, and MAP2. Image-derived cellular features were extracted from such images. (B) The two supervised ML classification algorithms linear discriminant analysis (LDA) and support vector machine (SVM) were trained to separate the two reference classes GS/+ and +/+ isogenic control mDANs. The additional mDAN control lines were then mapped to the reference classes’ feature space. (C) Leave-one-out cross-validation (LOOCV) to identify individual feature contributions to SVM classification of multiple cell lines in (B). (D) SVM classification of GS/+ and +/+ isogenic control mDANs and mapping of neurons treated with the LRRK2 inhibitors GNE-7915, MLi-2, and PFE-360 to the reference classes’ feature space. (E) Same experiment as in (D) but instead neurons were stained with Hoechst, tetramethylrhodamine (TMRM), and calcein. (F) LOOCV to identify individual feature contributions to SVM classification in (E). All imaging data were generated in duplicate experiments with multiple technical replicates. Each data point represents one well. Mann-Whitney U-testing was performed for significance testing. Notches in boxplots indicate the 95% confidence interval.

Using these ML classifiers, we tested whether PD-specific phenotypic neuronal fingerprints might exist. To evaluate the specificity of a GS/+ phenotypic fingerprint compared with the +/+ isogenic control line, we differentiated two additional and genetically unrelated control iPSC lines (EDi001-A-5 and GIBCO) into mDANs (Figure S3). All four mDAN cell lines were then stained with Hoechst and antibodies against αSyn, TH, and MAP2. We derived a total of 126 quantitative features from the images (Figure 3A, Table S1). 54 image features were TH+ cell type specific (43%) and specifically represented mDAN biology. We hypothesized that a weighted combination of all 126 cellular image features might allow the generation of a unique phenotypic fingerprint per cell line. Secondly, we hypothesized that the generated phenotypic fingerprint of GS/+ neurons would be significantly different from all control lines.

To test our hypotheses, we evaluated the two supervised ML classifiers LDA and SVM. In a first step, we determined the Pearson correlations of all image features to remove strongly correlated image features. Both LDA and SVM algorithms were then trained repeatedly on shuffled sets of 80% of the imaging data and tested on 20% of the imaging data. In total, training and testing were repeated 25 times on shuffled slices of the dataset in a process referred to as cross-validation (CV) (Figure 2B). CV is useful to detect and prevent overfitting and to increase robustness since the ML models are trained on multiple slightly different datasets. We observed that training variability over all cycles was generally low, indicating that sufficient training data were provided to both the LDA and SVM algorithms. Alternatively, we used data from one plate for model training, evaluated the model on two unseen plates, and obtained similar results (Figure S5A).

Although by eye the four mDAN lines appeared similar (Figure 3A), both LDA and SVM classification algorithms distinguished GS/+ neurons from control cell lines. Overall, control cell lines appeared more similar compared to each other than the GS/+ neurons. Next, we calculated the Z-factor between the GS/+ and +/+ neuronal classifications (Zhang et al., 1999). The SVM classification Z-factor was superior to the LDA Z-factor (0.12 versus 0.43) (Figure 3B). Based on these results, we focused mainly on SVM classification. To obtain a biological meaningful explanation of the classification results, we applied leave-one-out cross-validation (LOOCV). During LOOCV, each image feature is left out once, classification is performed repeatedly on the remaining image features, and the resulting Z-factor is calculated (Figure 2C). LOOCV demonstrated that our SVM results can most likely be explained by cell line differences concerning the ratio of MAP2-positive neurons and the level of αSyn (Figure 3C). To test whether strong, but more general cellular stress responses such as protein folding or reactive oxygen species induced stress could mimic the effects of a G2019S mutation, neurons were treated with tunicamycin or sodium arsenite (NaAsO_2_). Despite the presence of the stressors, LDA and SVM analysis did not detect any change in +/+ control neuron classification (Figure S6). Together these findings demonstrate that iPSC-derived mDANs can be classified based on a genetic mutation and image-extracted phenotypes.

### ML classification identifies LRRK2 inhibitor-treated neurons based on image-derived cellular features

Next, we asked whether SVM-driven analysis can detect chemical compound-induced phenotypic changes. We hypothesized that LRRK2 inhibitor treatment might partially rescue the previously observed combined feature phenotype (Figure 3B). Cryopreserved D30 mDANs were seeded in 384-well plates. 6 days after seeding the LRRK2 inhibitors GNE-7915, PFE-360, and MLi-2 were added for 24 h, and the neurons were fixed and stained using Hoechst, αSyn, TH, and MAP2 antibodies. Image-based feature extraction, data processing, and SVM model training were performed. The SVM classifier successfully distinguished +/+ and GS/+ mDANs treated with DMSO with a Z-factor of 0.13 (Figure 3D). Next, LRRK2 inhibitor-treated GS/+ mDANs were classified relative to the DMSO controls. GNE-7915 did not lead to phenotypic changes detectable in our assays and resembled the DMSO control classification. PFE-360 and MLi-2 induced subtle phenotypic differences detected by Hoechst/αSyn/TH/MAP2 staining and were classified as significantly different from DMSO-treated neurons. The shift toward the +/+ isogenic control was strongest for the PFE-360 treated GS/+ mDANs (Figure 3D). Additionally, we tested all three inhibitors in a second patient-derived PD mDAN model containing an SNCA gene triplication (Devine et al., 2011; Gwinn et al., 2011). We observed only modest GNE-7915 phenotypic effects and no detectable response to PFE-360 or MLi-2 (Figure S4). LRRK2 kinase inhibitors therefore seem to induce stronger phenotypic changes in LRRK2 mutated neurons than in SNCA triplication neurons.

Next, we tested whether LRRK2 inhibitor treatment would also lead to SVM-detectable changes on the mitochondrial level. Neurons were cultured and treated as before and stained with Hoechst, the live cell dye calcein, and the mitochondria-specific dye TMRM. 96 image features were calculated based on these three stainings (Table S2), and an SVM model was trained to distinguish +/+ from GS/+ mDANs. We then applied the SVM model to sets of mitochondrial image features from LRRK2 inhibitor-treated mDANs. Similar to the previous results obtained with the Hoechst/αSyn/TH/MAP2 staining, we detected only a weak effect of GNE-7915 on the measured mitochondrial phenotypes, while PFE-360 and MLi-2 treatment of GS/+ mDANs led to a classification shift toward +/+ control mDANs (Figure 3E). To identify the mitochondrial features most responsible for the observed classification result, we performed LOOCV analysis. We found that mitochondrial shape (i.e., compactness and form factor) as well as TMRM intensity contributed the most to the classification result (Figure 3F).

### Detection protein kinase C (PKC) agonist-treated single wells using multiple image-derived cellular features in LRRK2 G2019S neurons

**Figure 4:**
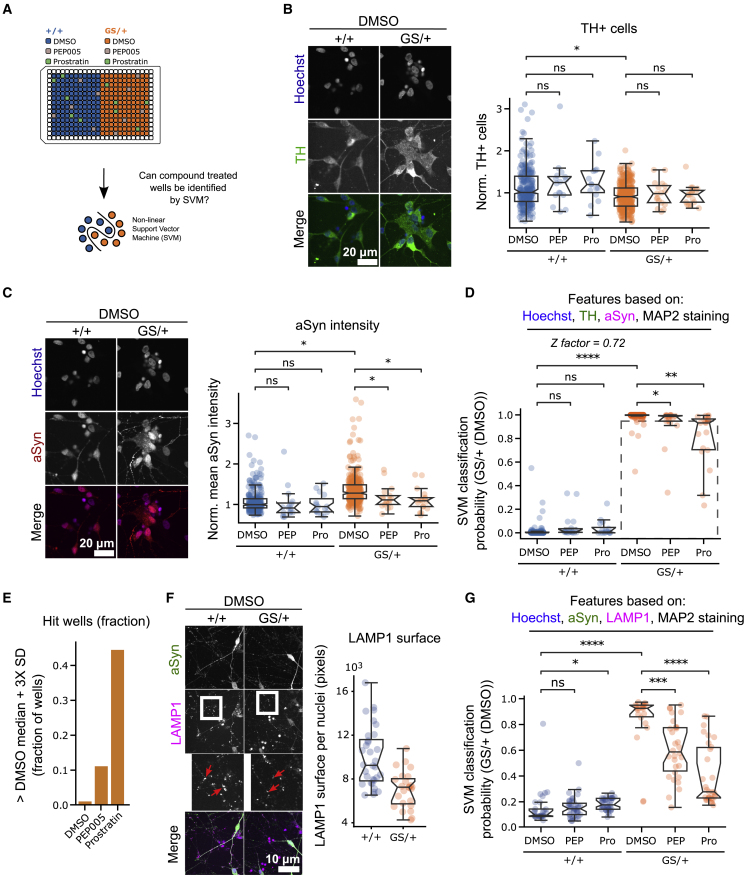
Machine learning (ML) can identify PKC agonist-treated LRRK2 G2019S mDANs in a simulated screening setup (A) Schematic depiction of experimental design. Single wells spiked with PEP005 or prostratin were randomly distributed over the plate. Support vector machine (SVM) classification was applied to identify these wells. (B) and (C) Representative images illustrate PEP005 and prostratin effects on the number of TH-positive cells and αSyn staining intensity. (D) SVM classification of GS/+ and +/+ isogenic control mDANs based on cellular image features extracted from Hoechst, αSyn, TH, and MAP2 staining. PEP005- and prostratin-treated wells were then mapped to the reference classes’ feature space. The broken square includes datapoints (wells) that are at a distance of more than three standard deviations (SDs) from the GS/+ DMSO-treated median. (E) Quantification of the fraction of wells more than three SDs from the GS/+ DMSO-treated class median. (F) Representative images illustrate LAMP1 staining and differences in surface area. (G) SVM classification of treated and untreated GS/+ and +/+ isogenic control mDANs based on cellular image features extracted from Hoechst, αSyn, LAMP1, and MAP2 staining. All imaging data were generated in triplicate experiments with multiple technical replicates. Each data point represents one well. Mann-Whitney U-testing was performed for significance testing. Notches in boxplots indicate the 95% confidence interval.

Recently, Laperle et al. demonstrated that lysosomal activation by phorbol esters, such as PEP005 and prostratin, reduced αSyn levels in iPSC-derived mDANs (Laperle et al., 2020). Given the established connection between LRRK2 and lysosomal biology, we hypothesized that PEP005 and prostratin might also be able to lower the elevated αSyn levels in our LRRK2 G2019S model and thereby shift multiple cellular phenotypes toward a control phenotype (Hockey et al., 2015; Obergasteiger et al., 2020). To demonstrate that ML classification can detect a chemical modulation in mDANs, we treated six randomly selected wells per plate with PEP005 or prostratin for 72 h (Figure 4A).

Next, cells were fixed and stained with Hoechst and αSyn, TH, and MAP2 antibodies, and 126 image features were extracted (Table S1). Verification of individual image features, such as the number of TH+ cells, showed that PEP005 and prostratin compound treatments were not toxic for either GS/+ nor +/+ neurons (Figure 4B). PEP005 and prostratin treatments led to a decrease in αSyn levels, specifically in GS/+ neurons, but not control +/+ neurons, confirming the initial results of Laperle et al. obtained in different PD mDAN lines (Figure 4C). Next, we trained an SVM model to distinguish +/+ from GS/+ mDANs using image-based features as input. Consistent with our previous results, SVM was able to separate both DMSO-treated control classes +/+ and GS/+ with high accuracy (0.98 ± SEM 0.02) and a Z-factor of 0.72 (Figure 4D). We then applied the SVM model to sets of image features originating from PEP005- and prostratin-treated wells. Compound-treated GS/+ neurons classified differently than the DMSO-treated GS/+ neurons. Although this effect was small for PEP005, most prostratin-treated wells shifted toward the +/+ isogenic control neurons. Additionally, we observed that +/+ control neurons responded less to PEP005 and prostratin treatment (Figure 4D).

To assess whether single PEP005- or prostratin-treated wells could be detected in a typical screen setup using only a small number of replicates, we determined a 3x standard deviation (SD) threshold around the median of the DMSO-treated GS/+ neurons. We calculated the percentage of compound-treated wells beyond the threshold that could be regarded as a hit. For GS/+ neurons treated with DMSO, less than 1% of wells were more than 3 SDs away from the median, while this was 11% of PEP005- and 43% of prostratin-treated wells (Figure 4E). To estimate the minimum number of wells required to determine whether a compound is active in a screening setup using neuronal profiles, we performed a calculation based on a typical GS/+ (DMSO) data distribution and measured how many replicate wells would be needed to detect at least one hit at least 3 SDs from the median. The calculation was repeated with different anticipated effect sizes and desired statistical power thresholds (Figures S5B–S5C). For example, given an effect size of 2 and a desired power of 0.85 to detect a deviation from the null hypothesis requires at least four replicates.

Since both LRRK2 as well as PEP005 and prostratin have been linked to lysosomal biology, we additionally investigated whether our assay would show improved compound detection using a lysosome-specific stain. A lysosome-associated membrane protein 1 (LAMP1) antibody was used to detect lysosomal image features. Several were altered in GS/+ neurons, such as decreased LAMP1 signal surface (Figure 4F). Treatment with PEP005 and prostratin and using multiple LAMP1 image features during SVM classification resulted in a larger shift of compound-treated GS/+ neuron-containing wells toward +/+ control neurons compared with lysosome-unspecific staining (Figures 4D and 4G).

### Detection PKC agonist-treated single wells using multiple image-derived cellular features in SNCA triplication neurons

**Figure 5:**
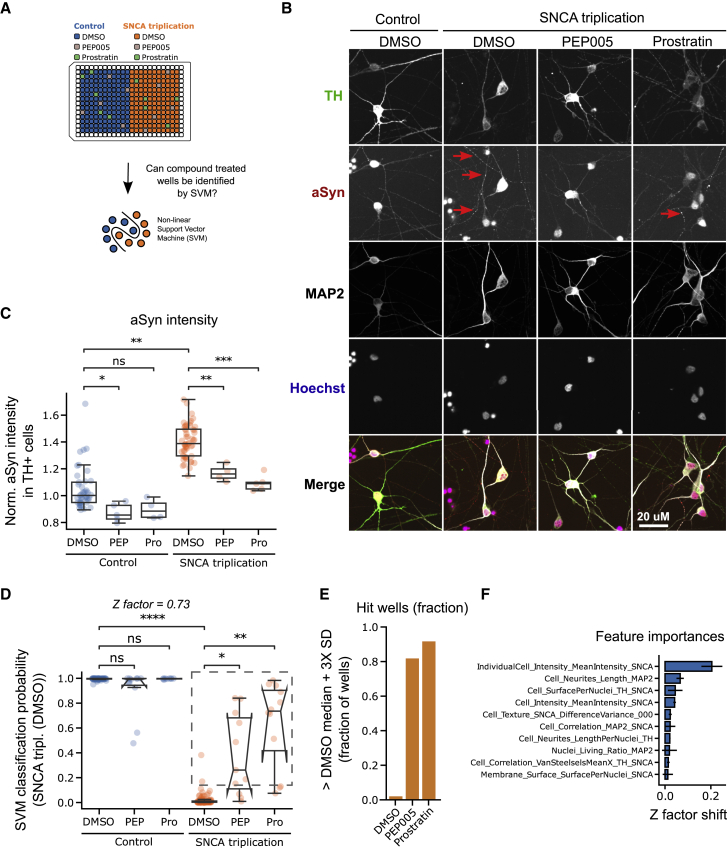
Machine learning (ML) can identify protein kinase C (PKC) agonist-treated SNCA triplication mDANs in a simulated screening setup (A) Schematic depiction of experimental design. Single wells spiked with PEP005 or prostratin were randomly distributed over the plate. Support vector machine (SVM) classification was applied to identify these wells. (B) Representative images of neurons stained with Hoechst and TH, αSyn, and MAP2 antibodies after 37 days of differentiation and treated with either DMSO, PEP005, or prostratin. Red arrows indicate αSyn staining in neurites. (C) Quantification of αSyn staining intensity across all treatment conditions. (D) SVM classification of SNCA triplication and isogenic control mDANs based on cellular image features extracted from Hoechst, αSyn, TH, and MAP2 staining. PEP005- and prostratin-treated wells were then mapped to the reference classes’ feature space. The broken square includes datapoints (wells) that are at a distance of more than three standard deviations (SDs) from the SNCA triplication DMSO-treated median. (E) Quantification of the fraction of wells at a distance of more than three SDs from the SNCA triplication DMSO-treated class median. (F) Leave-one-out cross-validation (LOOCV) to identify individual feature contributions to SVM classification in (D). All imaging data were generated in triplicate experiments with multiple technical replicates. Each data point represents one well. Mann-Whitney U-testing was performed for significance testing. Notches in boxplots indicate the 95% confidence interval.

To generalize our neuronal profiling approach, we established a second PD mDAN model based on SNCA gene triplication-carrying donor iPSCs expressing four copies of SNCA and an isogenic control (Figure S3). Using both cell lines, we performed a similar experiment as described in Figure 4A with the aim to detect individual wells treated with PEP005 or prostratin using SVM classification (Figure 5A). SNCA triplication mDANs showed signs of αSyn accumulation in dendrites and a reduced dendritic network (Figure 5B). Image feature quantification confirmed that indeed αSyn levels were increased in SNCA triplication mDANs. Additionally, we observed αSyn lowering of 15% by PEP005 and 25% by prostratin (Figure 5C). Next, we trained an SVM classifier to separate isogenic control from SNCA triplication mDANs. Similar to the LRRK2 model, the SVM algorithm was able to separate isogenic control from SNCA triplication mDANs with high accuracy (0.97 ± SEM 0.03) resulting in a Z-factor of 0.73 (Figure 5D). SVM classification of SNCA triplication mDANs treated with PEP005 or prostratin showed a shift toward isogenic control mDANs. Isogenic control neurons treated with both compounds had a similar image feature-based profile and were statistically indistinguishable from DMSO-treated control neurons, suggesting a specific effect of PEP005 and prostratin in SNCA triplication neurons (Figure 5D).

In SNCA triplication neurons treated with DMSO, less than 1% of wells were more than 3 SDs away from the median, while this was 81% of PEP005- and 91% of prostratin-treated wells (Figure 5E). The single most important image feature distinguishing SNCA cell lines was the αSyn staining intensity, a proxy for cellular αSyn content explaining 0.2 points of the observed 0.73 Z-factor (Figure 5F). We confirmed the contribution of αSyn content and other features by using LightGBM, a different classification algorithm. These findings in a second PD-relevant disease model indicate that bioactive molecules such as PEP005 and prostratin can be detected using neuronal profiles and a small number of technical replicates.

### mDAN characterization at differentiation D50 reveals altered image feature profiles compared with D37 neurons

**Figure 6:**
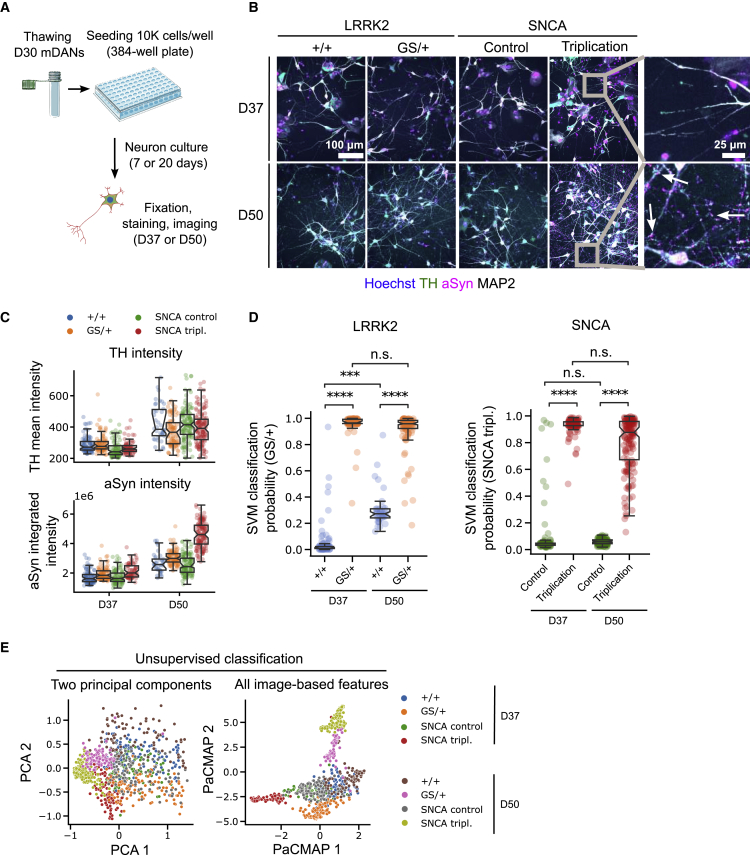
Imaging-based LRRK2 G2019S and SNCA triplication mDAN characterization at differentiation D50 reveals altered image feature profiles compared with D37 neurons (A) Schematic depiction of experimental design. (B) Representative images of neuronal cultures at differentiation D37 and D50. Arrows indicate clusters of αSyn staining signal. (C) Quantification of the TH and αSyn staining signal at D37 and D50. (D) Support vector machine (SVM) classification of SNCA triplication and isogenic control mDANs based on cellular image features extracted from Hoechst, αSyn, TH, and MAP2 staining. (E) Unsupervised classification of neuronal image feature profiles by principal component analysis (PCA) and pairwise controlled manifold approximation (PaCMAP). Imaging data were generated in triplicate experiments with multiple technical replicates per plate. Each data point represents one well. Mann-Whitney U-testing was performed for significance testing. Notches in boxplots indicate the 95% confidence interval.

To test whether multi-feature neuronal profiling could be applied to more mature neurons, we repeated classification experiments with 50-day-old SNCA and LRRK2 neurons and compared the results to 37-day-old neurons (Figures 6A and 6B). Levels of αSyn or TH were increased in D50 neurons, indicating increased maturity (Figure 6C). To test whether neuronal profiles were different between both time points, we classified D50 data using an SVM classification model developed with D37 data and found no significant differences, although some shifts were visible (Figure 6D). Unsupervised classification using principal component analysis (PCA) or PaCMAP (Wang et al., 2021) was able to separate all timepoints and genotypes from each other highlighting that detectable phenotypic alterations of neuronal profiles exist after prolonged maturation (Figure 6E).

## Discussion

In this study, we demonstrate that image-derived phenotypes in human iPSC-derived mDANs can be used for cell line stratification and the identification of chemical compound-treated neurons by ML classification approaches. iPSC-derived neurons are only rarely used in drug discovery due to complex cell culture protocols, long culture duration, and genetic or clonal heterogeneity (Cobb et al., 2018; Elitt et al., 2018). We applied multiple strategies to improve the reproducibility of our iPSC-derived neuron models. First, we worked with large, cryopreserved batches to reduce the number of required differentiations and used isogenic controls to reduce sources of inter-donor genetic variability. Additionally, we developed a compact and automated 7-day experimental protocol in 384-well-plate format to reduce intervention steps and technical variability.

The functions of LRRK2 are not fully understood, but it has become clear that LRRK2 can trigger autophosphorylation at Ser1292 and phosphorylate a subset of Rab small GTPases (Rab8A and Rab10) (Rocha et al., 2022; Sheng et al., 2012; Steger et al., 2016). A direct readout of these targets was not present in our panel of stains. This is likely the reason why one of the three tested LRRK2 inhibitors showed only little effects in our experimental setup. Similarly, the used phorbol esters PEP005 and prostratin have specific phosphorylation-inducing effects on PKC subunits α and δ (Hampson et al., 2005; Laperle et al., 2020; Mischak et al., 1993), which we did not examine directly in our phenotypic characterization. We observed PEP005 and prostratin effects in both the LRRK2, but especially the SNCA triplication model, likely because both molecules have αSyn-lowering capabilities in mDANs (Laperle et al., 2020). Additionally, we show that both compounds can be identified with higher confidence if a LAMP1 stain is used to extract lysosome-related image features. Using a multi-phenotype strategy to characterize or detect chemical compounds therefore requires a compromise between the identification and reversal of general PD hallmarks or the focus on a specific mode of action related to a class of compounds.

Two previous studies described compound screening in iPSC-derived mDANs using resistance to rotenone-induced apoptosis and neurite outgrowth (Tabata et al., 2018) or resistance to carbonyl cyanide *m*-chlorophenylhydrazone-induced apoptosis and rescued mitophagy as readouts (Yamaguchi et al., 2020). In contrast to previous work, we used ML classification to bundle multiple phenotypes, which offers certain advantages: the used cellular stainings allow the extraction of many PD-relevant image features and thereby create a more biologically diverse representation of mDANs amendable to chemical interventions. Second, the combination of multiple, including subtle, phenotypes is statistically more robust than single phenotypic approaches. Additionally, our ML classification approach allows us to determine which phenotypic features contributed to the overall phenotypic differences between healthy and disease mDANs and might therefore aid the target deconvolution process.

Future research will need to evaluate whether only effects of monogenetic alterations, such as the mutations in LRRK2 or SNCA genes tested here, lead to distinguishable phenotypes or whether also idiopathic forms of PD, in which no single disease cause is known, have unique phenotypic profiles. Recent work using fibroblasts from 91 PD patients among which were 32 idiopathic PD cases indeed point into the direction that global phenotypic PD profiles might exist (Schiff et al., 2022). We anticipate that image-based multidimensional readouts capturing multiple PD-relevant phenotypes might increase the chance to detect active chemical compounds that rescue not only an isolated phenotype, but an ensemble of disease-relevant phenotypes.

## Experimental procedures

### Generation of iPSC lines and differentiation into mDANs

All iPSC lines were generated by third parties and are deposited in the European Bank for Induced Pluripotent Stem Cells (EBiSC, https://cells.ebisc.org/) and listed in the Human Pluripotent Stem Cell Registry (hPSCreg, https://hpscreg.eu/) (Table S3). The original generators have obtained the informed consent from the donors. iPSCs were cultivated on Geltrex-coated (Thermo Fisher Scientific) dishes in StemMACS iPS-Brew XF (Miltenyi Biotech). The medium was changed daily, and cells were passaged twice a week using 0.5 mM EDTA in PBS (Thermo Fisher Scientific). Mycoplasma testing was performed twice per month.

mDANs were differentiated using a modified protocol based on Kriks et al. (Kriks et al., 2011; Ryan et al., 2013; Weykopf et al., 2019). Briefly, iPSCs were seeded onto Geltrex-coated six-well plates or T75 flasks at a density of 2 × 10^5^ cells/cm^2^ in StemMACS iPS-Brew XF containing 10 μM Y-27632 (Hiss). The next day, medium was switched to KnockOut DMEM medium containing KnockOut serum replacement (both Thermo Fisher Scientific) supplemented with 200 nM LDN19318 (Axon Medchem) and 10 μM SB431542 (Biozol) for dual SMAD-inhibition. On day 2, also 100 ng/mL Shh C24II (Miltenyi Biotech), 2 μM Purmorphamine (Miltenyi Biotec), 100 ng/mL FGF8 (Peprotech), and 3 μM CHIR99021 (Miltenyi Biotec) were added to the medium. After 5 days, medium was gradually shifted to Neurobasal medium (Thermo Fisher Scientific), and SB431542 was omitted from the medium. Starting at day 7, cells were grown only in the presence of LDN19318 and CHIR99021. On day 11, cells were switched to Neurobasal/B27/L-glutamine medium supplemented with CHIR99021 only. On day 13, cells were replated onto Geltrex-coated dishes in Neurobasal/B27/L-glutamine medium supplemented with 20 ng/mL BDNF, 20 ng/mL GDNF (both Cell Guidance Sys.), 221 μM L-ascorbic-acid (Sigma-Aldrich), 10 μM DAPT (Axon Medchem), 1 ng/mL TGF-βIII (Peprotech), 0.5 mM dibutyryl-cAMP (Enzo Life Sciences), and 10 μM Y-27632 (Hiss). Cells were maintained in the same medium but without Y-27632. Around day 23–25, cells were dissociated using StemPro Accutase (Thermo Fisher Scientific) and plated at a density of 1.4 x 105 cells/cm^2^ onto Geltrex-coated dishes. To eliminate non-neuronal cells, cultures were treated with 1 μg/mL Mitomycin C for 2 h on day 26. At day 30, neuronal cultures were dissociated using StemPro Accutase supplemented with 10 μM Y-27632 and singularized. Cells were counted and cryopreserved at 2.5 × 10^6^ cells/vial in CryoStor CS 10 (Sigma-Aldrich).

### Neuronal culture and compound treatment

30 DIV (days *in vitro*)-old neurons were thawed and centrifuged (400 *g*, 5 min, RT) in basal medium (Table S3) supplemented with ROCK inhibitor (Tocris #1254). Cell pellets were resuspended in differentiation medium (Table S3) supplemented with ROCK inhibitor. 384-well plates (Perkin Elmer, #6007558) were coated with 15 μg/mL poly-L-ornithine for 1 h at 37°C followed by 10 μg/ml laminin overnight at 4°C. Using trypan blue (Sigma, #T8154-20ML) and a Countess automated cell counter (Invitrogen), 10 × 10^3^ cells/well were seeded in 384-well plates. Edge wells were avoided for seeding and filled with PBS. Typically, thawed cells were incubated at 37°C and 5% CO_2_ for 7 days until 37 DIV with differentiation medium changes every other day. Plate coating, cell seeding, and medium changes were automated using an Agilent Bravo pipetting robot (Agilent) and EL406 plate washer and dispenser (Biotek). Compound treatment with 1 μM PEP005 (Tocris, #4054) and 5 μM prostratin (Tocris, # 5739) was performed at 34 DIV for 72 h until 37 DIV. Compound treatment with 0.1 μM GNE-7915 (MedChemExpress, #HY-18163), 0.1 μM MLi-2 (MedChemExpress, #HY-100411), 0.1 μM PFE-360 (MedChemExpress, #HY-120085), and 0.1 μM rotenone (Sigma, #R8875) was performed at 36 DIV for 24 h until 37 DIV. Treatment with 2 μM tunicamycin (MedChemExpress, #HY-A0098) and 50 μM sodium arsenite (Sigma, #1062771000) was performed for 3 h on DIV 37. For western blotting experiments, 5 μM AraC (Sigma, #C6645) was added for 24 h before cell lysis on DIV 37 or 44.

### *In situ* cytochemistry

Fixation was performed in 4% PFA (EMS Euromedex, #15710) for 20 min, followed by two PBS (Gibco, #14190) washes and permeabilization and blocking with 10% FBS (Gibco, #10270-106) and 0.1% Triton X-100 (Sigma, #T9284) dissolved in PBS for 1 h. Primary antibodies (Table S3) were prepared in antibody dilution buffer (PBS supplemented with 5% FBS and 0.1% Triton X-100) and incubated with the cells overnight at 4°C, followed by three PBS washes. Secondary antibodies and Hoechst (Table S3) in antibody dilution buffer were added to the cells for 1 h at RT, followed by three PBS washes. Mitochondrial imaging was performed in live cells. All dyes (Table S3) were prepared in differentiation medium and incubated with the cells for 30 min at 37°C and 5% CO_2_, followed by a wash with differentiation medium. Cells were imaged in a preheated microscope chamber at 37°C and 5% CO_2_. *In situ* cytochemistry was automated using an Agilent Bravo pipetting robot (Agilent) and EL406 plate washer and dispenser (Biotek).

### Imaging and image analysis

Imaging was performed on a Yokogawa CV7000 microscope in scanning confocal mode using a dual Nipkow disk. 384-well plates (Perkin Elmer, #6007558) were mounted on a motorized stage and images were acquired in a row-wise “zigzag” fashion at RT for fixed cells and 37°C and 5% CO_2_ for living cells. The system’s CellVoyager software and 405/488/561/640-nm solid laser lines were used to acquire single Z-plane 16-bit TIFF images through a dry 40× objective lens using a cooled sCMOS camera with 2,560 × 2,160 pixels and a pixel size of 6.5 μm without pixel binning. Nine images in a 3 × 3 orientation were acquired from the center of each well. Image segmentation and feature extraction was performed with an in-house software written in C++. Except for the detection of mitochondrial structures, image segmentation was performed on illumination-corrected raw images based on fluorescent channel intensity thresholds empirically determined per plate. Multiple quantitative image features were calculated (Tables S1 and S2). Mitochondrial structures and features were detected in rolling-ball background-subtracted and top-hat filtered images similar to a protocol described previously (Iannetti et al., 2016).

### ML analysis

To support the reproducibility of the ML method of this study, the ML summary table is included in the supplemental information per data, optimization, model, and evaluation (DOME) recommendations (Walsh et al., 2021) (Table S4). Multiple datasets were generated differing in terms of the used mDANs, chemical compound treatment, and fluorescent staining (Table S5). Input data were normalized, outliers were removed, and the number of input features was reduced by removing strongly correlated features (Figure 2, Table S4). We applied the Python-written ML library scikit-learn to train and test all models (Pedregosa et al., 2011). We primarily used supervised binary classification algorithms. Figure 2B summarizes the overall ML workflow. All models’ hyperparameters were optimized using scikit-learn’s GridSearchCV module and evaluated using k-fold cross-validation. Performance was checked using accuracy. All raw data are listed in Table S5 and are available together with the corresponding Jupyter notebook ML pipelines on GitHub (https://github.com/johanneswilbertz/mDA-neuron-classification).

### Statistics

All data were generated at least in duplicate with neurons from a single differentiation batch. All data are represented as boxplots. The notches of the box represent the 95% confidence interval of the median obtained by bootstrapping with parameter value 1,000. Each data point represents the mean of a single well of a 384-well plate comprised of nine images. Data from different plates were median normalized to allow comparison across plates acquired on different days. Data processing and plotting were carried out with Python packages Pandas (McKinney, 2010), Matplotlib (Hunter, 2007), and Seaborn (Waskom, 2021). Null hypothesis significance testing was performed with the freely available Python package Statannot (Weber, 2022). For data not displaying a normal distribution, the non-parametrical Mann-Whitney U-test was performed. For normally distributed data, Welch’s t test was applied. Statistical significance is presented in the figures as ^∗^p < 0.05, ^∗∗^p < 0.01, ^∗∗∗^p < 0.001, ^∗∗∗∗^p < 0.0001, and not significant (ns = p > 0.05).

## Author contributions

A.V. performed profiling experiments; L.C. developed and performed image analysis and data processing; Z.H. developed the ML pipeline; N.W.D. developed the image analysis software; B.W., M.S., and S.H. performed neuronal differentiation and quality controls; J.L. designed neuronal stainings; Y.J.K. initiated the study; M.P. and O.B. supervised differentiation experiments; A.O. supervised image analysis procedures; P.S. and L.B. co-supervised the study and designed experiments; J.H.W. co-supervised the study, designed experiments, conceptualized and performed image analysis and data processing, and wrote the manuscript with input from all authors.

## Supplemental information

Document S1. Figures S1–S6, Tables S1–S6, and Supplemental experimental procedures

Document S2. Article plus Supplemental Information
