# Supplementary material for: High-content phenotyping of Parkinson's disease patient stem cell-derived midbrain dopaminergic neurons using machine learning classification
Source: Stem Cell Reports. 2022 Sep 29;17(10):2349–64. doi: 10.1016/j.stemcr.2022.09.001 (PMC9561636; doi:10.1016/j.stemcr.2022.09.001)
Supplement: Document S1. Figures S1–S6, Tables S1–S6, and Supplemental experimental procedures [file mmc1.pdf]

Stem Cell Reports, Volume *17*

Supplemental Information

High-content phenotyping of Parkinson's disease patient stem cell-derived midbrain dopaminergic neurons using machine learning classification

Aurore Vuidel, Loïc Cousin, Beatrice Weykopf, Simone Haupt, Zahra Hanifehlou, Nicolas Wiest-Daesslé, Michaela Segschneider, Joohyun Lee, Yong-Jun Kwon, Michael Peitz, Arnaud Ogier, Laurent Brino, Oliver Brüstle, Peter Sommer, and Johannes H. Wilbertz

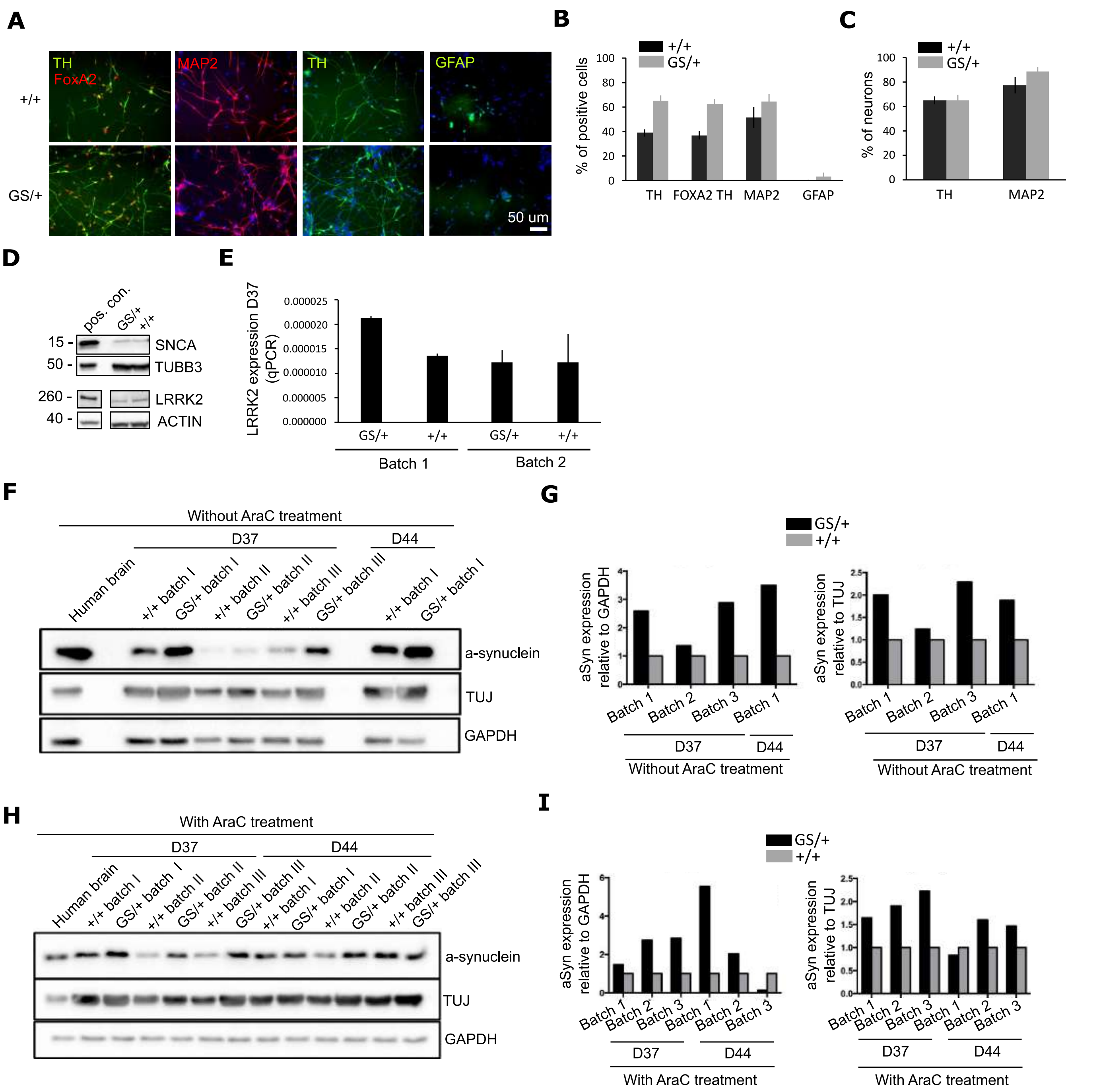

Figure S1 (referring to Figure 1): Characterization of human iPSC-derived LRRK2 G2019S mDA neurons. (A) Batches of +/+ isogenic control and GS/+ iPSCs were differentiated into mDA neurons for 30 days and cryo-preserved at 2x106 cells per well. Thawed neurons were cultured for additional 7 days and stained for Tyrosine hydroxylase (TH), Forkhead box protein A2 (FOXA2), Microtubule-associated protein 2 (MAP2), and Glial Fibrillary Acidic Protein (GFAP). **(B)** The purity of the mDA neuron population after differentiation and thawing was measured by the ratio of neuronal marker MAP2, dopaminergic marker TH, midbrain dopaminergic markers TH/FOXA2, and astrocyte marker GFAP positive cells. **(C)** The ratio of TH and MAP2 positive cells was calculated for the β-tubulin III (TUBB3) staining-positive neuronal population. **(D)** The presence of the proteins α-synuclein, TUBB3, and LRRK2 in the neuronal culture was confirmed by Western blotting. **(E)** qPCR analysis showed the expression of LRRK2 in isogenic control +/+ neurons as well as in mutation carrying GS/+ neurons. **(F)** D30 mDA neurons were cryopreserved, thawed and cultured for either 7 days (D37) or 14 days (D44) and lysed. α-synuclein, β-tubulin III (TUJ antibody) and GAPDH were detected by Western blotting. **(G)** Western blots were analyzed densiometrically and the α-synuclein level was expressed relative to GAPDH (left panel) or TUJ signal (right panel). **(H)** Experiment performed as in **(F)**, but cultured neurons were treated with the cytostatic drug cytosine arabinoside (AraC) in order to prevent glial proliferation. **(I)** Analysis was performed as in **(G)**.

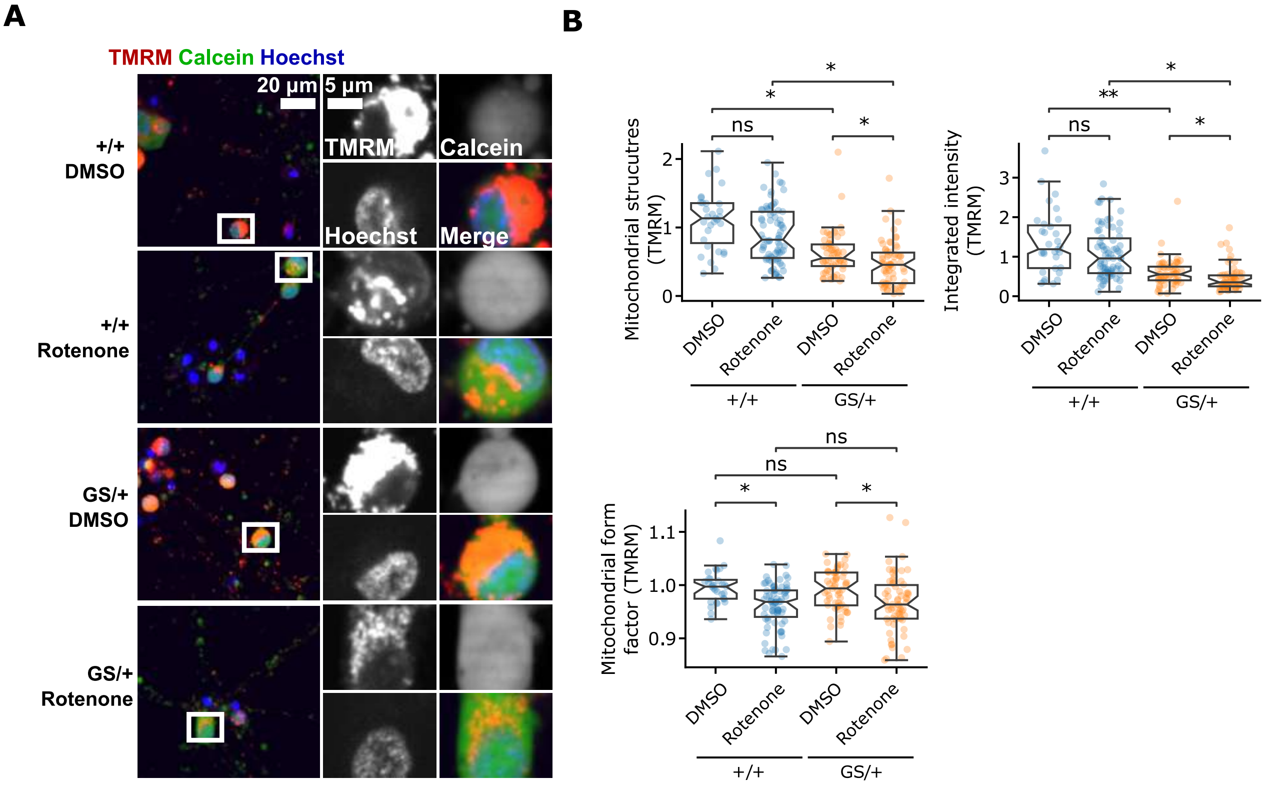

Figure S2 (referring to Figure 1): LRRK2 G2019S mDA neurons are more sensitive to the mitochondrial stressor Rotenone. (**A**) Representative images of cryopreserved D30 mDA neurons cultured for 7 days and treated with DMSO or Rotenone during the last 24 hours. Cells were stained with Hoechst, Calcein and TMRM and imaged. (**B**) Multiple mitochondrial features were quantified based on the TMRM stain including mitochondrial number, intensity, and shape.

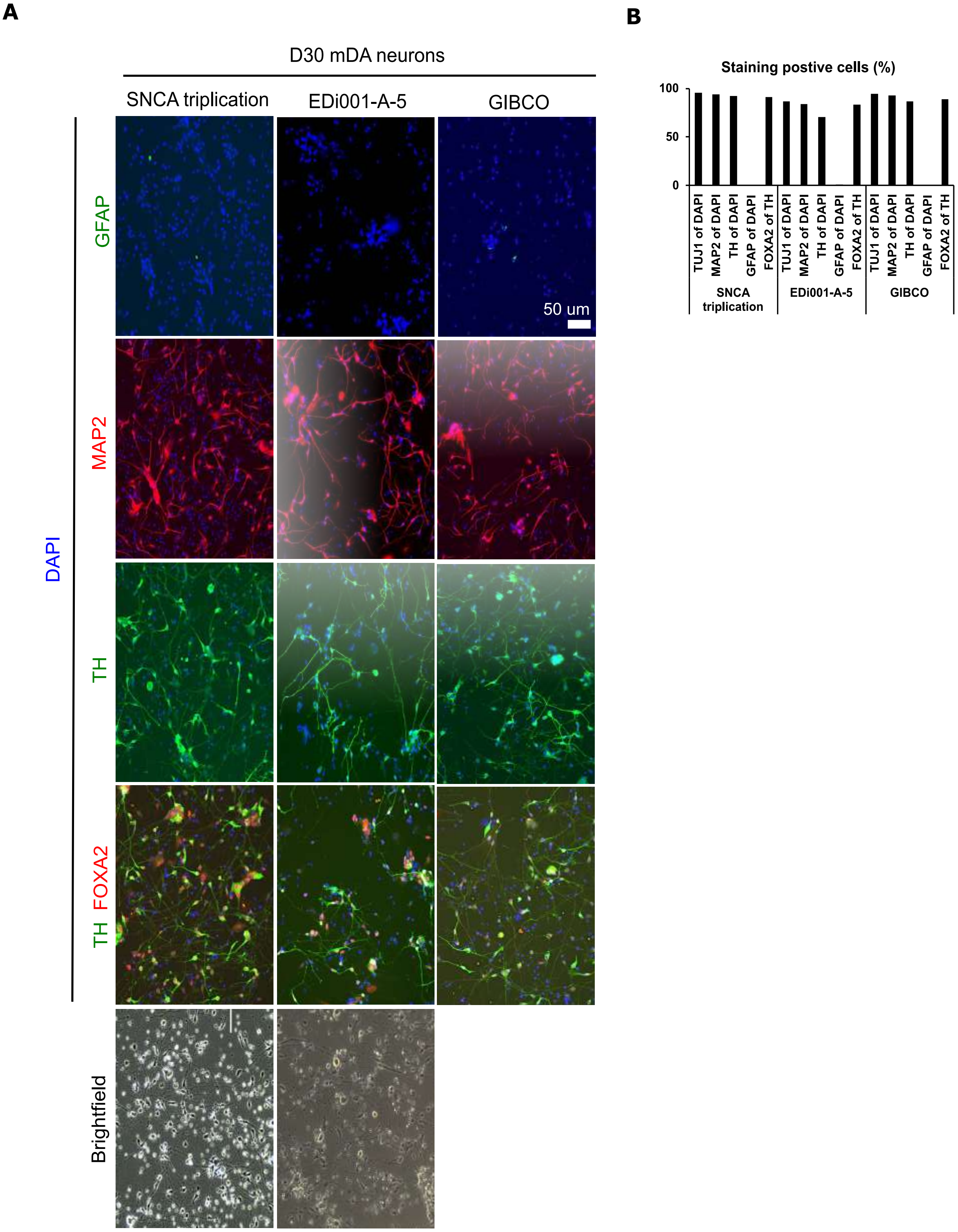

Figure S3 (referring to Figure 5): Characterization of additional (non-LRRK2) human iPSC-derived mDA neurons. (**A**) Batches of SNCA triplication, isogenic control (Edi001-A-5) and a genetically unrelated control (Gibco) iPSCs were differentiated into mDA neurons for 30 days and cryo-preserved at 2x106 cells per well. Thawed neurons were cultured for additional 7 days and stained for Tyrosine hydroxylase (TH), Forkhead box protein A2 (FOXA2), Microtubule-associated protein 2 (MAP2), and Glial Fibrillary Acidic Protein (GFAP). Representative images are shown. (**B**) Quantification of staining positive cells.

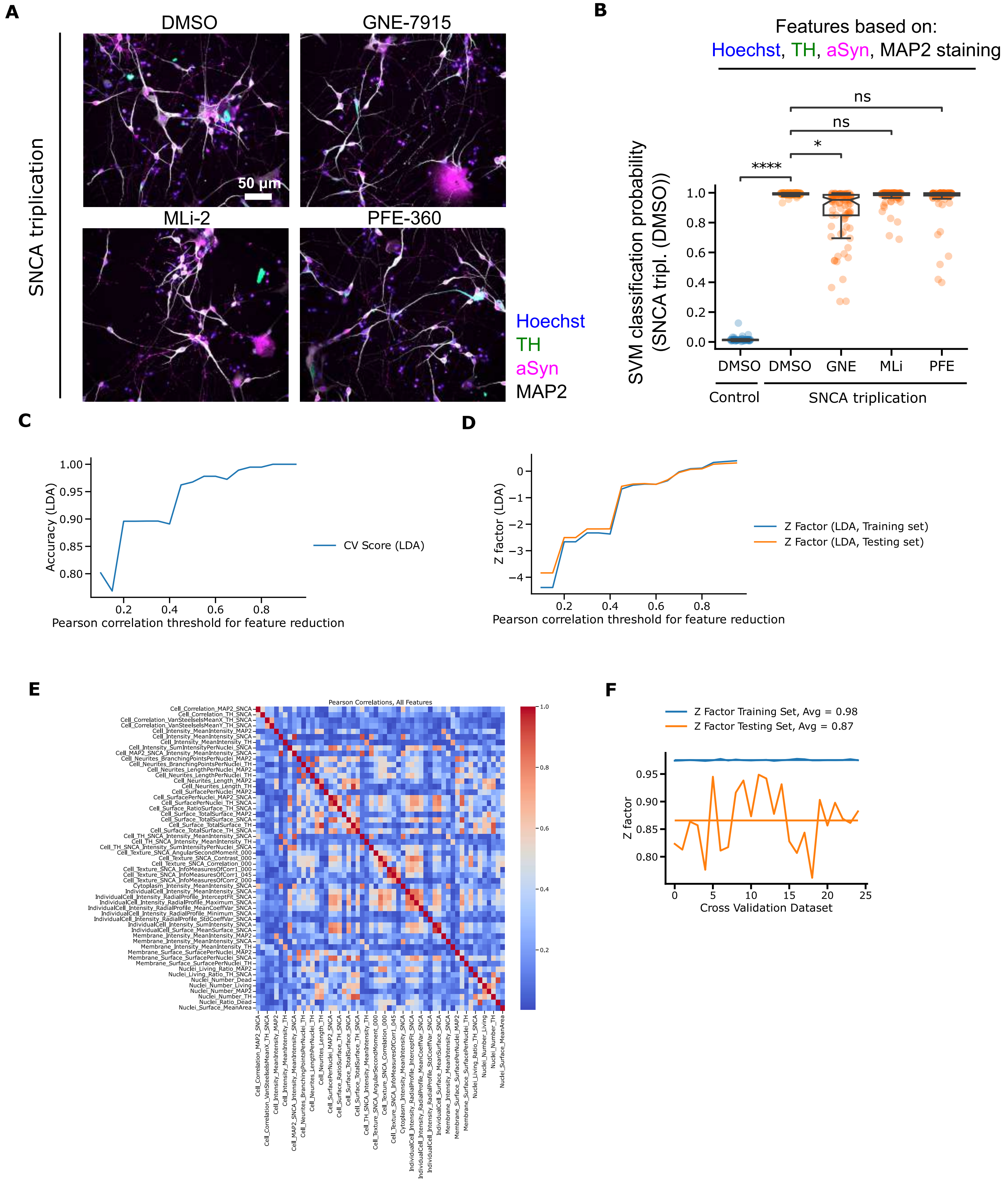

Figure S4 (referring to Figure 3): Feature selection, training and testing of Support Vector Machine (SVM) classifier using Hoechst/TH/α-synuclein/MAP2 staining derived features originating from SNCA triplication and isogenic control neurons. (**A**) Representative images of mDA neurons treated with vehicle control DMSO or LRRK2 inhibitors GNE-7915, MLi-2, and PFE-360. (**B**) SVM classification of SNCA triplication and isogenic control mDANs and mapping of neurons treated with the LRRK2 inhibitors GNE-7915, MLi-2 and PFE-360 to the reference classes' feature space. (**C**) Linear Discriminant Analysis (LDA) classification was used to select image features based on Hoechst/TH/α-synuclein/MAP2 staining that are not strongly correlated. LDA classifier accuracy is shown as a function of Pearson's correlation thresholds. (D) LDA training and testing set Z-factors between isogenic control and SNCA triplication reference classes as a function of Pearson's correlation thresholds used to exclude correlated image features. (E) Pearson's correlation matrix of all selected image features for model training with threshold 0.95. (**F**) Performance of SVM classifier during 25 cycles of training (80% of data) and testing (20% of data) using 25 shuffled data sets. Z-factor values were calculated between isogenic control and SNCA triplication reference classes.

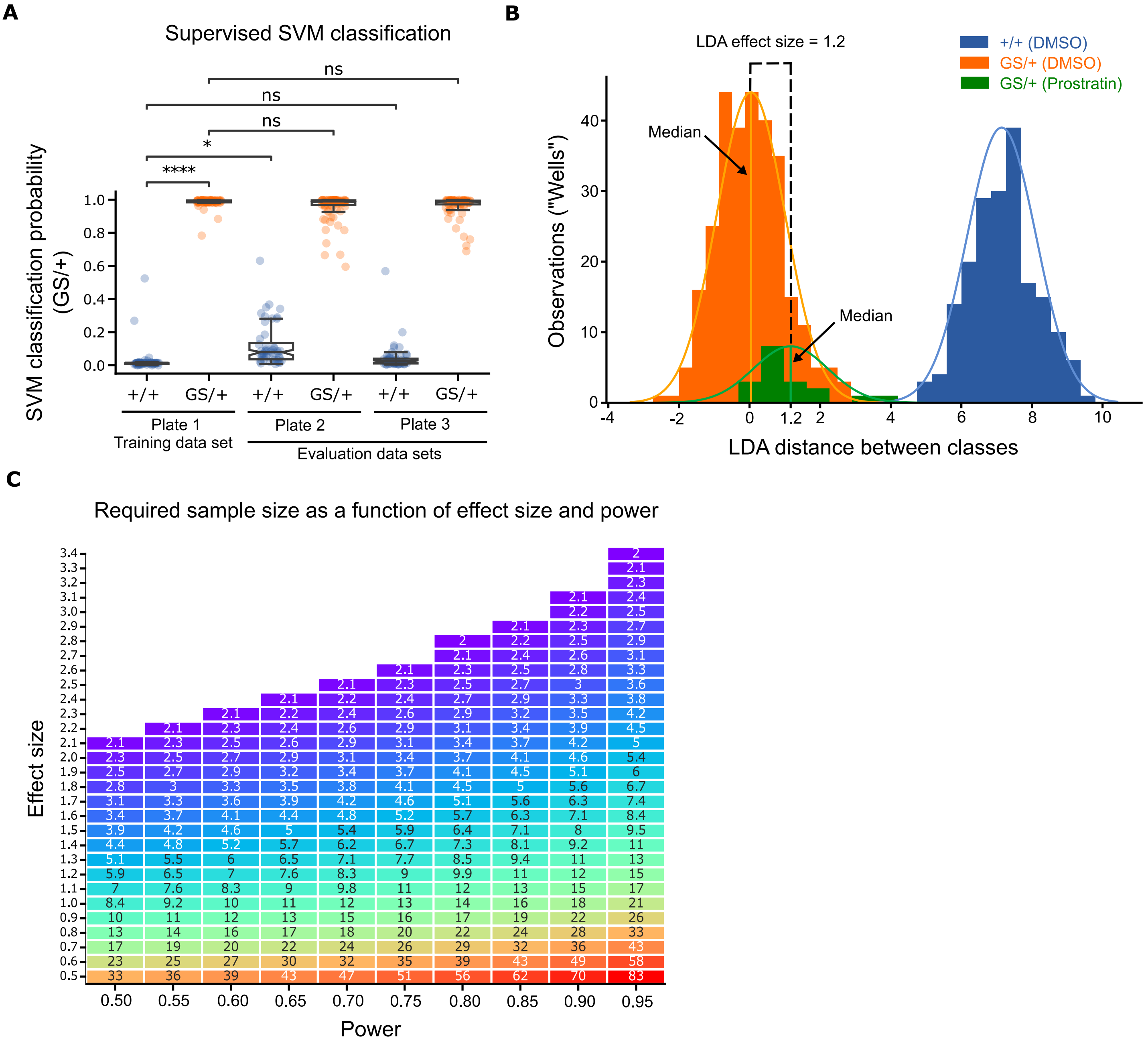

Figure S5 (referring to Figures 3-5): Parameter determination for neuronal image-based phenotyping. (**A**) A Support Vector Machine (SVM) model was trained to separate the two reference classes GS/+ and +/+ isogenic control mDANs originating from a single 384-well plate (Plate 1). Image features based on Hoechst, TH, α-synuclein and MAP2 staining were used. To evaluate the trained model, GS/+ and +/+ neuronal image features originating from two additional plates (Plate 2 and 3) where then mapped to the reference classes' feature space originating from Plate 1. (**B**) Distributions of LDA distances between experimental classes. The same data as in Figure 4 was used and Gaussian distributions were fitted. The broken lines indicate the effect size of 1.2 between the two treatment conditions GS/+ (DMSO) and GS/+ (Prostratin). (**C**) Required sample size (number of wells) as a function of the expected effect size and desired power to obtain at least one hit larger than the GS/+ (DMSO) median + 3x SD. The experimentally determined GS/+ (DMSO) distribution in (B) was used to calculate the median + 3x SDs window. Power can be defined as the probability that a test of significance will pick up on an effect that is present. Values >0.8 are typically desired.

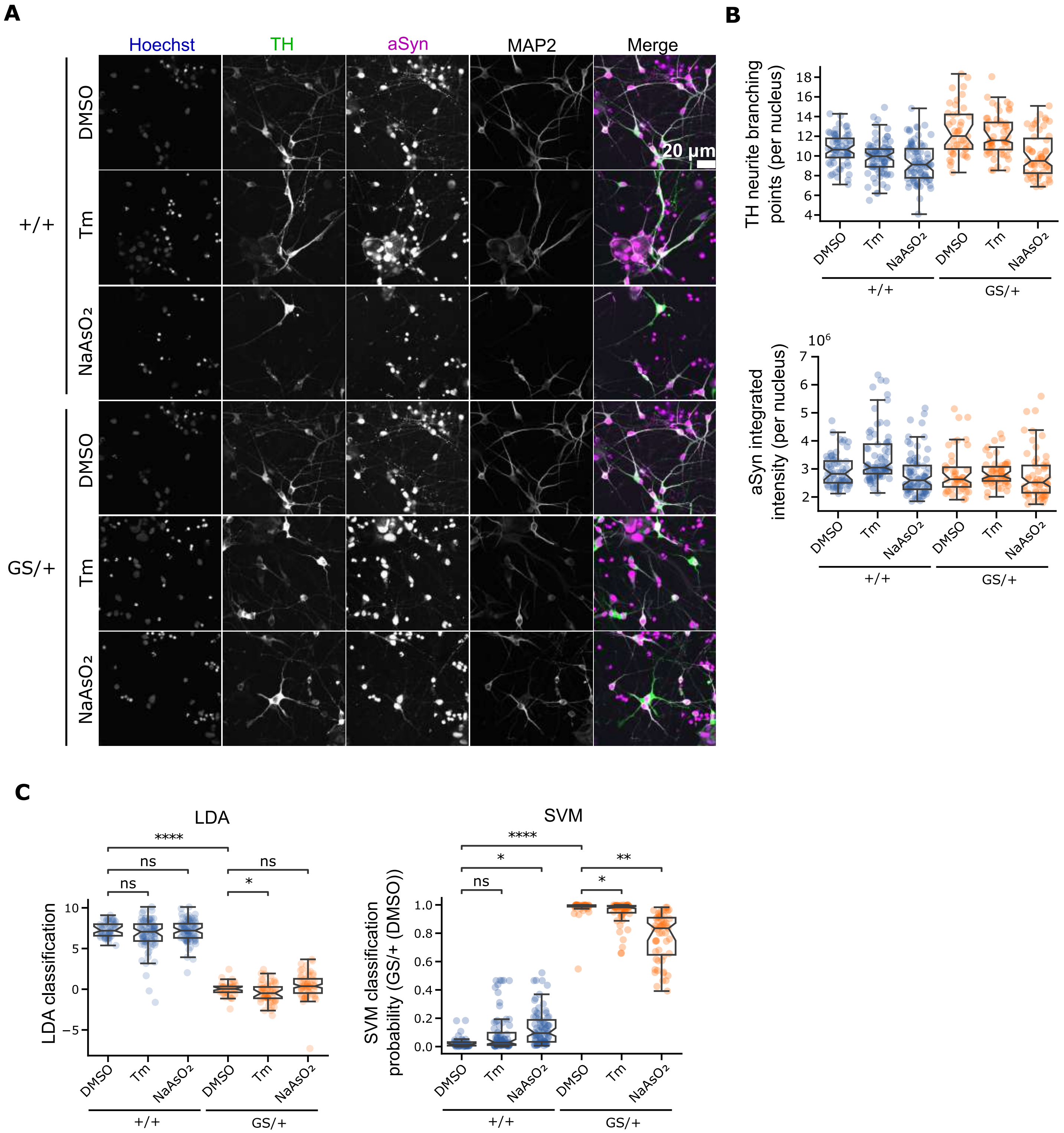

Figure S6 (referring to Figure 3): Multi-image feature guided detection of chemically stressed mDA neurons. (**A**) Representative images of mDA neurons treated for 3 hours with DMSO, 2µM Tunicamycin (Tm) or 50µM Sodium Arsenite ($NaAsO_2$). (**B**) Based on raw images, tyrosine hydroxylase (TH) neurite branching points and α-synuclein staining intensity was qunatified. **(C)** Linear Discriminant Analysis (LDA) and Support Vector Machine (SVM) classification of GS/+ and +/+ control mDANs based on cellular image features extracted from Hoechst, α-synuclein, TH, and MAP2 staining. Imaging data was generated in duplicate experiments with multiple technical replicates per plate. Each data point represents one well. Mann-Whitney U-testing was performed for significance testing. Notches in boxplots indicate the 95% confidence interval.

Table S1: Description of extracted image features after Hoechst/TH/α-synuclein/MAP2 staining and fluorescence channel segmentation.

	Feature name	Feature description
1	Cell_Correlation_MAP2_SNCA	Pearson correlation between MAP2 and SNCA channel
2	Cell_Correlation_TH_SNCA	Pearson correlation between TH and SNCA channel
3	Cell_Correlation_VanSteelselsMeanX_TH_SNCA	Van Steelsel's cross correlation between TH and SNCA channel, shift on x-axis
4	Cell_Correlation_VanSteelselsMeanY_TH_SNCA	Van Steelsel's cross correlation between TH and SNCA channel, shift on y-axis
5	Cell_Correlation_VanSteelselsSigmaX_TH_SNCA	SD of Van Steelsel's cross correlation between TH and SNCA channel, shift on x-axis
6	Cell_Correlation_VanSteelselsSigmaY_TH_SNCA	SD of Van Steelsel's cross correlation between TH and SNCA channel, shift on y-axis
7	Cell_Intensity_MeanIntensity_MAP2	Mean pixel intensity of MAP2 channel
8	Cell_Intensity_MeanIntensity_SNCA	Mean pixel intensity of SNCA channel
9	Cell_Intensity_MeanIntensity_TH	Mean pixel intensity of TH channel
10	Cell_Intensity_SumIntensityPerNuclei_SNCA	Integrated pixel intensity of SNCA channel normalized to number of nuclei
11	Cell_MAP2_SNCA_Intensity_MeanIntensity_SNCA	Mean pixel intensity of SNCA channel colocalized to MAP2 channel
12	Cell_Neurites_BranchingPointsPerNuclei_MAP2	Dendritic branching points of MAP2 channel normalized to number of nuclei
13	Cell_Neurites_BranchingPointsPerNuclei_TH	Dendritic branching points of TH channel normalized to number of nuclei
14	Cell_Neurites_LengthPerNuclei_MAP2	Dentritic network length of MAP2 channel normalized to number of nuclei
15	Cell_Neurites_LengthPerNuclei_TH	Dentritic network length of TH channel normalized to number of nuclei
16	Cell_Neurites_Length_MAP2	Dentritic network length of MAP2 channel
17	Cell_Neurites_Length_TH	Dentritic network length of TH channel
18	Cell_SurfacePerNuclei_MAP2	Surface pixels occupied by MAP2 channel, normalized to number of nuclei
19	Cell_SurfacePerNuclei_MAP2_SNCA	Surface pixels occupied by colocalized MAP2 and SNCA channel normalized to number of nuclei
20	Cell_SurfacePerNuclei_SNCA	Surface pixels occupied by SNCA channel, normalized to number of nuclei
21	Cell_SurfacePerNuclei_TH	Surface pixels occupied by TH channel, normalized to number of nuclei
22	Cell_SurfacePerNuclei_TH_SNCA	Surface pixels occupied by colocalized TH and SNCA channel normalized to number of nuclei
23	Cell_Surface_RatioSurface_TH_SNCA	Surface ratio occupied by colocalized TH and SNCA channel
24	Cell_Surface_TotalSurface_MAP2	Surface pixels occupied by MAP2 channel
25	Cell_Surface_TotalSurface_SNCA	Surface pixels occupied by SNCA channel
26	Cell_Surface_TotalSurface_SNCA_MAP2	Surface pixels occupied by colocalized SNCA and MAP2 channel
27	Cell_Surface_TotalSurface_TH	Surface pixels occupied by TH channel
28	Cell_Surface_TotalSurface_TH_SNCA	Surface pixels occupied by colocalized TH and SNCA channel
29	Cell_TH_SNCA_Intensity_MeanIntensity_SNCA	Mean pixel intensity of SNCA channel colocalized to TH channel
30	Cell_TH_SNCA_Intensity_MeanIntensity_TH	Mean pixel intensity of TH channel colocalized to SNCA channel
31	Cell_TH_SNCA_Intensity_SumIntensityPerNuclei_SNCA	Integrated pixel intensity of SNCA channel colocalized to TH channel normalized to number of nuclei
32	Cell_Texture_SNCA_AngularSecondMoment_000	Haralick uniformity of distribution of gray levels at 0 degree shift
33	Cell_Texture_SNCA_AngularSecondMoment_045	Haralick uniformity of distribution of gray levels at 45 degree shift
34	Cell_Texture_SNCA_AngularSecondMoment_090	Haralick uniformity of distribution of gray levels at 90 degree shift
35	Cell_Texture_SNCA_AngularSecondMoment_135	Haralick uniformity of distribution of gray levels at 135 degree shift
36	Cell_Texture_SNCA_Contrast_000	Haralick contrast of gray levels at 0 degree shift
37	Cell_Texture_SNCA_Contrast_045	Haralick contrast of gray levels at 45 degree shift
38	Cell_Texture_SNCA_Contrast_090	Haralick contrast of gray levels at 90 degree shift
39	Cell_Texture_SNCA_Contrast_135	Haralick contrast of gray levels at 135 degree shift
40	Cell_Texture_SNCA_Correlation_000	Haralick correlation of gray levels at 0 degree shift
41	Cell_Texture_SNCA_Correlation_045	Haralick correlation of gray levels at 45 degree shift
42	Cell_Texture_SNCA_Correlation_090	Haralick correlation of gray levels at 90 degree shift
43	Cell_Texture_SNCA_Correlation_135	Haralick correlation of gray levels at 135 degree shift
44	Cell_Texture_SNCA_DifferenceEntropy_000	Haralick difference of randomness of gray levels at 0 degree shift
45	Cell_Texture_SNCA_DifferenceEntropy_045	Haralick difference of randomness of gray levels at 45 degree shift
46	Cell_Texture_SNCA_DifferenceEntropy_090	Haralick difference of randomness of gray levels at 90 degree shift
47	Cell_Texture_SNCA_DifferenceEntropy_135	Haralick difference of randomness of gray levels at 135 degree shift
48	Cell_Texture_SNCA_DifferenceVariance_000	Haralick difference of variance of gray levels at 0 degree shift
49	Cell_Texture_SNCA_DifferenceVariance_045	Haralick difference of variance randomness of gray levels at 45 degree shift
50	Cell_Texture_SNCA_DifferenceVariance_090	Haralick difference of variance randomness of gray levels at 90 degree shift
51	Cell_Texture_SNCA_DifferenceVariance_135	Haralick difference of variance randomness of gray levels at 135 degree shift
52	Cell_Texture_SNCA_Entropy_000	Haralick randomness of gray levels at 0 degree shift
53	Cell_Texture_SNCA_Entropy_045	Haralick randomness of gray levels at 45 degree shift
54	Cell_Texture_SNCA_Entropy_090	Haralick randomness of gray levels at 90 degree shift
55	Cell_Texture_SNCA_Entropy_135	Haralick randomness of gray levels at 135 degree shift
56	Cell_Texture_SNCA_InfoMeasuresOfCorr1_000	Haralick information measure of correlation 1 of gray levels at 0 degree shift
57	Cell_Texture_SNCA_InfoMeasuresOfCorr1_045	Haralick information measure of correlation 1 of gray levels at 45 degree shift
58	Cell_Texture_SNCA_InfoMeasuresOfCorr1_090	Haralick information measure of correlation 1 of gray levels at 90 degree shift
59	Cell_Texture_SNCA_InfoMeasuresOfCorr1_135	Haralick information measure of correlation 1 of gray levels at 135 degree shift
60	Cell_Texture_SNCA_InfoMeasuresOfCorr2_000	Haralick information measure of correlation 2 of gray levels at 0 degree shift
61	Cell_Texture_SNCA_InfoMeasuresOfCorr2_045	Haralick information measure of correlation 2 of gray levels at 45 degree shift
62	Cell_Texture_SNCA_InfoMeasuresOfCorr2_090	Haralick information measure of correlation 2 of gray levels at 90 degree shift
63	Cell_Texture_SNCA_InfoMeasuresOfCorr2_135	Haralick information measure of correlation 2 of gray levels at 135 degree shift
64	Cell_Texture_SNCA_InverseDiffMoment_000	Haralick homogeneity of gray levels at 0 degree shift
65	Cell_Texture_SNCA_InverseDiffMoment_045	Haralick homogeneity of gray levels at 45 degree shift
66	Cell_Texture_SNCA_InverseDiffMoment_090	Haralick homogeneity of gray levels at 90 degree shift
67	Cell_Texture_SNCA_InverseDiffMoment_135	Haralick homogeneity of gray levels at 135 degree shift
68	Cell_Texture_SNCA_SumAverage_000	Haralick sum of averages of gray levels at 0 degree shift
69	Cell_Texture_SNCA_SumAverage_045	Haralick sum of averages of gray levels at 45 degree shift
70	Cell_Texture_SNCA_SumAverage_090	Haralick sum of averages of gray levels at 90 degree shift
71	Cell_Texture_SNCA_SumAverage_135	Haralick sum of averages of gray levels at 135 degree shift
72	Cell_Texture_SNCA_SumEntropy_000	Haralick sum of gray level randomness at 0 degree shift
73	Cell_Texture_SNCA_SumEntropy_045	Haralick sum of gray level randomness at 45 degree shift
74	Cell_Texture_SNCA_SumEntropy_090	Haralick sum of gray level randomness at 90 degree shift
75	Cell_Texture_SNCA_SumEntropy_135	Haralick sum of gray level randomness at 135 degree shift
76	Cell_Texture_SNCA_SumOfSquares_000	Haralick sum of square gray level variance at 0 degree shift
77	Cell_Texture_SNCA_SumOfSquares_045	Haralick sum of square gray level variance at 45 degree shift
78	Cell_Texture_SNCA_SumOfSquares_090	Haralick sum of square gray level variance at 90 degree shift
79	Cell_Texture_SNCA_SumOfSquares_135	Haralick sum of square gray level variance at 135 degree shift
80	Cell_Texture_SNCA_SumVariance_000	Haralick sum of gray level variance at 0 degree shift

Table S1 (continued)

81	Cell_Texture_SNCA_SumVariance_045	Haralick sum of gray level variance at 45 degree shift
82	Cell_Texture_SNCA_SumVariance_090	Haralick sum of gray level variance at 90 degree shift
83	Cell_Texture_SNCA_SumVariance_135	Haralick sum of gray level variance at 135 degree shift
84	Cytoplasm_Intensity_MeanIntensity_SNCA	Mean pixel intensity of cytoplasmic SNCA channel
85	Cytoplasm_MAP2_SNCA_Intensity_MeanIntensity_SNC	Mean pixel intensity of cytoplasmic SNCA channel colocalized to MAP2 channel
86	Cytoplasm_SurfacePerNuclei_SNCA	Surface pixels occupied by cytoplasmic SNCA channel normalized to number of nuclei
87	Cytoplasm_SurfacePerNuclei_TH_SNCA	Surface pixels occupied by colocalized cytoplasmic TH and SNCA channel normalized to number of nuclei
88	Cytoplasm_Surface_TotalSurface_SNCA	Surface pixels occupied by cytoplasmic SNCA channel
89	IndividualCell_Intensity_MeanIntensity_SNCA	Mean pixel intensity of SNCA channel based on all individually segmented cells
90	IndividualCell_Intensity_RadialProfile_InterceptFit_SNC	Fitted intercept of SNCA channel decay from center to edge
91	IndividualCell_Intensity_RadialProfile_MaxSlope_SNCA	Maximum steepness of SNCA channel decay from center to edge
92	IndividualCell_Intensity_RadialProfile_Maximum_SNCA	Maximum intensity of SNCA channel from center to edge
93	IndividualCell_Intensity_RadialProfile_MeanCoeffVar_SN	Mean SNCA channel dispersion from center to edge
94	IndividualCell_Intensity_RadialProfile_MeanGradient_SN	Mean shape of SNCA channel decay from center to edge
95	IndividualCell_Intensity_RadialProfile_Mean_SNCA	Mean intensity of SNCA channel from center to edge
96	IndividualCell_Intensity_RadialProfile_Median_SNCA	Median intensity of SNCA channel from center to edge
97	IndividualCell_Intensity_RadialProfile_Minimum_SNCA	Minimum intensity of SNCA channel from center to edge
98	IndividualCell_Intensity_RadialProfile_Q1_SNCA	First quartile intensity of SNCA channel from center to edge
99	IndividualCell_Intensity_RadialProfile_Q3_SNCA	Third quartile intensity of SNCA channel from center to edge
100	IndividualCell_Intensity_RadialProfile_SlopeFit_SNCA	Fitted slope of SNCA channel decay from center to edge
101	IndividualCell_Intensity_RadialProfile_StdCoeffVar_SNC	SD of SNCA channel dispersion from center to edge
102	IndividualCell_Intensity_RadialProfile_Std_SNCA	SD of SNCA channel intensity from center to edge
103	IndividualCell_Intensity_SumIntensity_SNCA	Integrated pixel intensity of SNCA channel based on all individually segmented cells
104	IndividualCell_Surface_MeanSurface_SNCA	Mean surface pixels occupied by SNCA channel based on all individually segmented cells
105	Membrane_Intensity_MeanIntensity_MAP2	Mean pixel intensity of MAP2 channel on cellular edge
106	Membrane_Intensity_MeanIntensity_SNCA	Mean pixel intensity of TH channel on cellular edge
107	Membrane_Intensity_MeanIntensity_TH	Mean pixel intensity of MAP2 channel on cellular edge
108	Membrane_Surface_SurfacePerNuclei_MAP2	Surface pixels on cellular edge occupied by MAP2 channel normalized to number of nuclei
109	Membrane_Surface_SurfacePerNuclei_SNCA	Surface pixels on cellular edge occupied by SNCA channel normalized to number of nuclei
110	Membrane_Surface_SurfacePerNuclei_TH	Surface pixels on cellular edge occupied by TH channel normalized to number of nuclei
111	Nuclei_Living_Ratio_MAP2	Ratio of MAP2 channel positive nuclei
112	Nuclei_Living_Ratio_MAP2_SNCA	Ratio of MAP2 and SNCA channel positive nuclei
113	Nuclei_Living_Ratio_SNCA	Ratio of SNCA channel positive nuclei
114	Nuclei_Living_Ratio_TH	Ratio of TH channel positive nuclei
115	Nuclei_Living_Ratio_TH_SNCA	Ratio of TH and SNCA channel positive nuclei
116	Nuclei_Number_Big	Number of large nuclei
117	Nuclei_Number_Dead	Number of condensed/bright nuclei
118	Nuclei_Number_Living	Number of nuclei based on Hoechst channel
119	Nuclei_Number_MAP2	Number of MAP2 channel positive nuclei
120	Nuclei_Number_MAP2_SNCA	Number of MAP2 and SNCA channel positive nuclei
121	Nuclei_Number_SNCA	Number of SNCA channel positive nuclei
122	Nuclei_Number_TH	Number of TH channel positive nuclei
123	Nuclei_Number_TH_SNCA	Number of TH and SNCA channel positive nuclei
124	Nuclei_Ratio_Dead	Ratio of condensed/bright nuclei
125	Nuclei_Ratio_Living	Ratio of nuclei not considered condensed/bright
126	Nuclei_Surface_MeanArea	Mean surface pixels of Hoechst channel

Table S2: Description of extracted image features after Hoechst/Calcein/TMRM staining and fluorescence channel segmentation.

	Feature name	Feature description
1	avg_compactness_all	Average Compactness = area * 4 * pi / major axis : all the components
2	avg_compactness_calcein	Average Compactness = area * 4 * pi / major axis : only the components in the calcein mask
3	avg_compactness_not_calcein	Average Compactness = area * 4 * pi / major axis : only the components not in the calcein mask
4	avg_eccentricities_all	Average Eccentricity = sqrt(1 - (minor axis/major axis)^2) : all the components
5	avg_eccentricities_not_calcein	Average Eccentricity = sqrt(1 - (minor axis/major axis)^2) : only the components not in the calcein mask
6	avg_form_factor_all	Average Form Factor = 4*pi*area/perimeter : all the components
7	avg_form_factor_calcein	Average Form Factor = 4*pi*area/perimeter : only the components in the calcein mask
8	avg_form_factor_not_calcein	Average Form Factor = 4*pi*area/perimeter : only the components not in the calcein mask
9	avg_intensity_calcein	Average intensity of the calcein channel
10	avg_intensity_tmrm	Average intensity of the TMRM channel
11	avg_major_axis_all	Average major axis : all the components
12	avg_major_axis_calcein	Average major axis : only the components in the calcein mask
13	avg_major_axis_not_calcein	Average major axis : only the components not in the calcein mask
14	avg_minor_axis_all	Average minor axis : all the components
15	avg_minor_axis_calcein	Average minor axis : only the components in the calcein mask
16	avg_minor_axis_not_calcein	Average minor axis : only the components not in the calcein mask
17	avg_nb_tmrm_calcein_per_nuclei	Average number of components tmrm in calcein mask normalized per nulcei TMRM positive
18	avg_nb_tmrm_per_nuclei	Total number of components tmrm normalized per nulcei TMRM positive
19	avg_perimeters_all	Average perimeter of components : all the components
20	avg_perimeters_calcein	Average perimeter of components : only the components in the calcein mask
21	avg_perimeters_not_calcein	Average perimeter of components : only the components not in the calcein mask
22	compo_avg_intensity_tmrm_calcein	Average TMRM intensity per component : only the components in the calcein mask
23	compo_avg_intensity_tmrm_not_calcein	Average TMRM intensity per component : only the components not in the calcein mask
24	compo_avg_sum_intensity_tmrm_calcein	Average sum of TMRM intensity per component : only the components in the calcein mask
25	compo_avg_sum_intensity_tmrm_not_calcein	Average sum of TMRM intensity per component : only the components not in the calcein mask
26	compo_avg_surface_tmrm_calcein	Average surface of TMRM components : only the components in the calcein mask
27	compo_avg_surface_tmrm_not_calcein	Average surface of TMRM components : only the components not in the calcein mask
28	dead_nuclei	Number of dead cells
29	living_nuclei	Number of living cells
30	nuclei_tot	Total number of cells
31	ratio_dead_nuclei	Ratio of dead cells
32	ratio_living_nuclei	Ratio of living cells
33	ratio_nb_tmrm_calcein	Ratio of TMRM components in the calcein mask
34	ratio_nb_tmrm_not_calcein	Ratio of TMRM components in the not calcein mask
35	sum_intensity_calcein_per_nuclei	Sum of intensities of calcein channel normalized by living cells
36	sum_intensity_calcein_tot	Sum of intensities of calcein channel
37	sum_intensity_calcein_tot_per_nuclei	Sum of intensities of calcein channel normalized by living cells
38	sum_intensity_tmrm_calcein	Sum of intensities of TMRM channel in the calcein mask
39	sum_intensity_tmrm_calcein_per_nuclei	Sum of intensities of TMRM channel in the calcein mask normalized by calcein cell positive cell number
40	sum_intensity_tmrm_not_calcein	Sum of intensities of TMRM channel not in the calcein mask
41	sum_intensity_tmrm_not_calcein_per_nuclei	Sum of intensities of TMRM channel not in the calcein mask normalized by calcein cell positive cell number
42	sum_intensity_tmrm_tot	Sum of intensities of TMRM channel
43	sum_intensity_tmrm_tot_per_nuclei	Sum of intensities of TMRM channel normalized by living cell number
44	surface_calcein	Surface of calcein channel above the threshold
45	surface_calcein_per_nuclei_calcein	Surface of calcein channel above the threshold normalized by calcein positive cell number
46	surface_tmrm_calcein	Total surface of TMRM in calcein mask.
47	surface_tmrm_per_nuclei_not_calcein	Surface of TMRM not in the calcein mask normalized by negative calcein cell number
48	surface_tmrm_per_nuclie_calcein	Surface of TMRM in the calcein mask normalized by positive calcein cell number
49	total_nb_tmrm	Total number of components TMRM
50	total_nb_tmrm_calcein	Total number of components TMRM in calcein mask
51	avg_eccentricities_calcein	Average Eccentricity = sqrt(1 - (minor axis/major axis)^2) : only the components in the calcein mask
52	avg_nb_tmrm_not_calcein_per_nuclei	Number of TMRM components not in calcein mask normalized by negative calcein cell number
53	surface_tmrm	Total surface of TMRM channel above the threshold
54	total_nb_tmrm_not_calcein	Total number of TMRM components not in the calcein mask

Table S3: Used cell lines, antibodies, primers, and key reagents.

**Cell lines**					
**Genotype**	**hPSCreg name**	**Donor source**	**Provider**	**Reprogramming method**	**Ref**
LRRK2 G2019S	STBCi004-B (GS/+)	Female, dermal fibroblasts	Distributor: EBiSC; Generator: StemBANCC	Non-integrating Sendai virus	(Morrison et al., 2015)
Corrected LRRK2 G2019S mutation in STBCi004-B	STBCi004-B-1 (+/+)	Female, dermal fibroblasts	Distributor: EBiSC; Generator: StemBANCC	Non-integrating Sendai virus	(Morrison et al., 2015)
SNCA triplication	EDi001-A (AST23)	Female, dermal fibroblasts	Distributor: EBiSC; Generator: University of Edinburgh	Integrating Retro virus	(Devine et al., 2011; Gwinn et al., 2011)
Corrected SNCA triplication in EDi001-A	EDi001-A-5 (AST23-2KO-8B)	Female, dermal fibroblasts	Distributor: EBiSC; Generator: University of Edinburgh	Integrating Retro virus	(Devine et al., 2011; Gwinn et al., 2011)
No known mutations	TMOi001-A (Gibco A18944)	Female, CD34+ cord blood	Distributor: EBiSC; Generator: ThermoFisher	Non-integrating Epstein-Barr virus	(Burridge et al., 2011)

**Antibodies**			
**Antibody**	**Assay**	**Dilution**	**Distributor**
TH	ICC (quality control)	1/1500, 5% BSA + PBS	Millipore, #AB152
FOXA2	ICC (quality control)	1/200 5% BSA + PBS	Biotechne, #AF2400
MAP2	ICC (quality control)	1/1000, 5% BSA + PBS	Sigma, #M4403
GFAP	ICC (quality control)	1/1500, 5% BSA + PBS	Merck, #AB5804
α-synuclein	WB	1/1000 in Invitrogen™ iBind™ Flex Solution Kit	Novus, #NBP1-05194
TUBB3/TUJ	WB	1/1000, 5% milk + TBS-T, 2h, RT	Cell Signaling, #5568
LRRK2	WB	1/500, 5% milk + TBS-T, 2h, RT	NeuroMab, #N241A/34
Actin	WB	1/1000, 5% milk + TBS-T, 2h, RT	Cell Signaling, #58169
GAPDH	WB	1/1000 in Invitrogen™ iBind™ Flex Solution Kit	Cell Signaling, #2118
Anti-Rabbit IgG HRP-linked	WB	1/1000, 5% milk + TBS-T, 1h, RT	Cell Signaling, #7074S
α-synuclein rabbit	ICC	1/500, 5% FBS + 0.1% Triton X-100 + PBS, overnight, 4°C	Abcam, #138501
TH	ICC	1/1000, 5% FBS + 0.1% Triton X-100 + PBS, overnight, 4°C	Merck, #T2928
MAP2	ICC	1/5000, 5% FBS + 0.1% Triton X-100 + PBS, overnight, 4°C	Novus, #NB300-213
pS129 α-synuclein	ICC	1/500, 5% FBS + 0.1% Triton X-100 + PBS, overnight, 4°C	Cell Signaling, #23706S
α-synuclein mouse	ICC	1/500, 5% FBS + 0.1% Triton X-100 + PBS, overnight, 4°C	BD Biosciences, #610787
Alexa Fluo 488, Anti-Mouse	ICC	1/1000, 5% FBS + 0.1% Triton X-100 + PBS, 1h, RT	ThermoFisher, #A11001
Alexa Fluor 647 Anti-Chicken	ICC	1/250, 5% FBS + 0.1% Triton X-100 + PBS, 1h, RT	Jackson Immuno Research, #703-605-155

Table S3 (continued)

Alexa Fluo 555, Anti-Rabbit	ICC	1/1000, 5% FBS + 0.1% Triton X-100 + PBS, 1h, RT	ThermoFisher, #A21429		
TMRM	ICC	25nM, Differentiation medium, 30 min, 37°C	ThermoFisher, #T668		
Calcein	ICC	1.25μM, Differentiation medium, 30 min, 37°C	ThermoFisher, #C3100MP		
Hoechst 33342	ICC	1/2000, 30 min, 37°C for live staining or 1/3000 1h, RT for ICC	Sigma, # 14533		
**Primers**					
**LRRK2 Primer**	**Melting temp (˚C)**	**Annealing temp (˚C)**	**GC content (%)**	**Sequence**	**Product size (bp)**
FWD	58.98	55.98	50	GCTTGTTGTTGGACAGCTGA	Genomic: 1262
REV	58.97	55.97	50	GCTTGTTGTTGGACAGCTGA	mRNA: 224
**Media**					
**Reagents**	**Stock**	**Dilution**	**Distributor**		
Basal medium					
Neurobasal medium		1	Gibco, #21103-049		
GlutaMAX	100x	1:100	Gibco, #25030-081		
Pen/Strep	100x	1:100	Gibco, #15070-063		
B27	50x	1:50	Gibco, #12587-010		
Differentiation medium					
Basal medium		1			
BDNF	10 μg/mL	1:500	Cell Guidance Sys., #GFH1-10		
GDNF	10 μg/mL	1:500	Cell Guidance Sys., #GFH2-10		
LAAP	221 mM	1:1000	Sigma, #A8960-5G		
DAPT	10 mM	1:1000	Axon Medchem, #1484		
TGF-β3	1 μg/mL	1:1000	Peprotech, #100-36E		
dbcAMP	100 mM	1:200	Enzo, #BML-CN125-0100		

Table S4: Machine learning (ML) summary table as per data, optimization, model and evaluation (DOME) recommendations (Walsh et al., 2021).

**Data**	Provenance	Source of all used data where the experiments and image analysis described in this paper. **Table S5** contains information on all generated datasets.
	Data splits	Available data was split into 80% training data and 20% testing data. No separate validation set was used due to the use of k-fold cross validation.
	Redundancy between data splits	Train and test sets were generated using scikitlearn's train_test_split function with enabled stratification and random seeding to ensure that relative class frequencies were approximately preserved in each train and test set.
	Availability of data	All data is available in the **Supporting Material**. Data splits can be reproduced using the provided Jupyter Notebooks (link to GitHub in online version of paper).
**Optimization**	Algorithm	Established supervised binary classification algorithms were used. LDA was used for feature reduction and initial classification. Non-linear SVM proved to be more accurate and was subsequently used for classification tasks followed by leave-one-out (LOO) analysis to determine feature contributions. Tree-based LightGBM was used to confirm feature contributions determined by LOO.
	Meta-prediction	LDA was used during the feature selection process. See also "Features" section.
	Data encoding	Outliers were removed by applying a 3xSD window around each feature's median. Data was transformed on the same scale using the following formula: $(x_{Feature\ N} – median_{Feature\ N})/SD$.
	Parameters	SVM parameters were systematically identified using scikitlearn's GridSearchCV function. The parameters of all other models were determined empirically.<br>LDA: solver='eigen', n_components=1, shrinkage='auto'<br>SVM: probability=True, kernel='rbf', C=0.1-0.5 (depends on dataset, see provided Jupyter notebooks), gamma='scale'<br>LightGBM: boosting_type='goss', n_estimators=10000, class_weight = 'balanced'
	Features	**Table S5** contains information on all generated datasets including the total number of features. For feature reduction, Pearson correlation was used to exclude strongly correlated image features. For each dataset, LDA was used to determine a Pearson correlation cut-off value which maximized the accuracy of the classification and the Z factor between the two reference classes. The selected number and type of features are detailed per data set in the provided Jupyter notebooks.
	Fitting	Since the number of total features was high (>50) we used several approaches to prevent overfitting. GridSearchCV was used to optimize the SVM regularization parameter C. For LDA shrinkage was set to 'auto'. Additionally, we used k-fold cross validation to use a maximum of data for training and to prevent data leaking into the test set.
	Regularization	GridSearchCV was used to optimize the SVM regularization parameter C. The LDA parameter shrinkage was set to 'auto'.
	Availability of configuration	All configurations of all models for all data sets are reported in the provided Jupyter Notebooks (link to GitHub in online version of paper).
**Model**	Interpretability	Due to its non-linear nature the used SVM algorithm is not fully interpretable, but we performed LOO analysis to extract the approximate contribution of each used feature to the prediction.
	Output	SVM provides the classification probability to belong to one of the two used classes.
	Execution time	The entire workflow completes in ca. 80 seconds on a standard desktop computer.
	Availability of software	Jupyter Notebooks for each used data set are provided on GitHub. All dependencies are Python-based and freely available.
**Evaluation**	Evaluation method	25-fold cross-validation was used to validate the SVM and LDA models for all datasets.
	Performance measures	F1 score, recall and accuracy are reported.
	Comparison	We did not benchmark our models with previously existing data, since appropriate mDA neuron data is not available.
	Confidence	F1 score, recall and accuracy are reported together with confidence intervals.
	Availability of evaluation	Model evaluation can be reproduced using the Jupyter Notebooks for each used data set provided on GitHub.

Table S5: Overview of generated data sets for ML analysis.

Dataset	Figure in study	Cellular staining for image feature calculation	Dataset composition
1	Figure 3B-C	Hoechst/TH/aSyn/MAP2	N (imaged wells) = 504<br>N (total image features) = 126
2	Figure 3D	Hoechst/TH/aSyn/MAP2	N (imaged wells) = 502<br>N (total image features) = 126
3	Figure 3E	Hoechst/Calcein/TMRM	N (imaged wells) = 308<br>N (total image features) = 96
4	Figure 4D-E & Figure S5	Hoechst/TH/aSyn/MAP2	N (imaged wells) = 716<br>N (total image features) = 126
5	Figure 5C-E	Hoechst/TH/aSyn/MAP2	N (imaged wells) = 521<br>N (total image features) = 126
6	Figure S4	Hoechst/TH/aSyn/MAP2	N (imaged wells) = 560<br>N (total image features) = 126
7	Figure 6C-E	Hoechst/TH/aSyn/MAP2	N (imaged wells) = 1064<br>N (total image features) = 126
8	Figure 4F-G	Hoechst/LAMP2/aSyn/MAP2	N (imaged wells) = 196<br>N (total image features) = 57
9	Figure S6	Hoechst/TH/aSyn/MAP2	N (imaged wells) = 378<br>N (total image features) = 126

Supplemental experimental procedures

Western blotting
Samples were extracted from two differentiation batches of GS/+ and +/+ mDA neurons. Cell lysis was performed in RIPA buffer. Human cortex lysate served as control. Samples were loaded in NuPAGE LDS Sample Buffer (4x) (ThermoFisher, #NP0007) and ran on NUPAGE Novex 3-8% Tris-acetate gels (ThermoFisher, #EA03752BOX) immersed in NUPAGE Tris-acetate SDS running buffer (1x) (ThermoFisher, #LA0041) for 1 hour at 150V. Gels were transferred to PVDF membranes (Biorad, #1620177) in transfer buffer (25mM Tris, 192mM Glycine, 10% Methanol (pre-cooled)) overnight at 60V and room temperature. Membrane blocking was performed in 5% skim milk powder (ThermoFisher, #LP0031B) in TBS-T for 1 hour at room temperature. Used primary and secondary antibodies are summarized in **Table S3**. The membrane was developed using Super Signal® West Femto Maximum Sensitivity Substrate (ThermoFisher, #34095).

RT-qPCR
Samples were extracted from two differentiation batches of GS/+ and +/+ mDA neurons. Sample extraction was performed using a Maxwell 16 total RNA purification kit and the Maxwell RSC instrument. RNA concentration was determined using a NanoDrop ™. Quality control was performed using an Agilent 2100 Bioanalyzer. All samples had a RNA integrity number (RIN) value of 10. cDNA synthesis was performed using the qScript cDNA synthesis kit (Quantabio, #95047) on 500 ng RNA. The cDNA was diluted 1:5 in ddH_20 and GoTaq SYBR green 2x Super Mix (Promega) was used on a ViiA 7 Real-Time PCR System (ThermoFisher) using the primers indicated in Table S3.

Multi-electrode array (MEA)
Cryopreserved 30 DIV (days in vitro) old neurons were thawed in a water bath and centrifuged (400g, 5 min, RT) in basal medium (Table S3) supplemented with ROCK inhibitor (Tocris, #1254). Cell pellets were resuspended in differentiation medium (Table S3) supplemented with ROCK inhibitor. 48-well CytoView MEA plates (Axion Biosystems, #M768-tMEA-48W) were coated with 15 µg/ml Polyethyleneimine solution (Sigma, #P3143) for 1 hour at 37°C followed by 10µg/ml Laminin coating for 2 hours at 37°C. Using Tryphan Blue (Sigma, # T8154-20ML) and a Countess automated cell counter (Invitrogen), 80×10^3 cells/well were seeded in the CytoView plate. Cells were incubated at 37°C and 5% CO_2 for 31 days until 61 DIV with differentiation medium changes twice a week. Once a week, 1µg/ml Laminin was added to the fresh media to maintain cell attachment. Before each media change, the electrical activity was recorded on the Maestro Pro multiwell MEA (Axion Biosystems) using the AxIS Navigator software (version 3.5.1, Axion Biosystems). Electrical activity was recorded for 5 minutes at a 12.5 kHz sampling rate and a 5.5 standard deviation threshold level for action potential detection. Before plate loading the device was allowed to equilibrate for approximately 30 minutes to 37°C and 5% CO_2. Data analyses were performed using the Neural Metric Tool (version 3.1.7, Axion Biosystems). Activity is expressed as the mean firing rate across electrodes in a well. The activity represents the action potentials, defining the neuron function. Synchrony is expressed via the area under the well pooled inter-electrode cross-correlation normalized to the auto-correlations. Synchrony reflects the strength of synaptic connections, and thus how likely neurons are to generate action potentials simultaneously.

Dimensionality reduction
For dimensionality reduction we used the scikit-learn package’s KernelPCA algorithm and PaCMAP (https://github.com/YingfanWang/PaCMAP). For PCA the following parameters were empirically chosen: n_components=2 and kernel='sigmoid'. For PaCMAP we performed a systematic parameter search and identified n_neighbors=25, MN_ratio=0.25, FP_ratio=0.5, and num_iters=5000 as ideal parameters to resolve the data structure. The raw data can be found in the Supplemental Information and the pipeline used for dimensionality reduction is available as a Jupyter notebook on GitHub (https://github.com/johanneswilbertz/mDA-neuron-classification).
