# Supplementary material for: High-content phenotyping of Parkinson's disease patient stem cell-derived midbrain dopaminergic neurons using machine learning classification
Source: Stem Cell Reports. 2022 Sep 29;17(10):2349–64. doi: 10.1016/j.stemcr.2022.09.001 (PMC9561636; doi:10.1016/j.stemcr.2022.09.001)
Supplement: Document S2. Article plus Supplemental Information [file mmc2.pdf]

Stem Cell Reports
Resource
OPEN ACCESS

High-content phenotyping of Parkinson's disease patient stem cell-derived midbrain dopaminergic neurons using machine learning classification

Aurore Vuidel,[1,6] Loïc Cousin,[1,6] Beatrice Weykopf,[2,3,4] Simone Haupt,[3,5] Zahra Hanifehlou,[1] Nicolas Wiest-Daesslé,[1] Michaela Segschneider,[2] Joohyun Lee,[1] Yong-Jun Kwon,[1] Michael Peitz,[2] Arnaud Ogier,[1] Laurent Brino,[1] Oliver Brüstle,[2,3] Peter Sommer,[1] and Johannes H. Wilbertz[1,*]

[1]Ksilink, Strasbourg, France
[2]Institute of Reconstructive Neurobiology, University of Bonn Medical Faculty & University Hospital Bonn, Bonn, Germany
[3]LIFE & BRAIN GmbH, Bonn, Germany
[4]Present address: Harvard Medical School and Brigham & Women's Hospital, Precision Neurology Program & APDA Center for Advanced Parkinson Research, Boston, MA, USA
[5]Present address: University of Cologne, Cologne, Germany
[6]Co-senior author
*Correspondence: johannes.wilbertz@ksilink.com
https://doi.org/10.1016/j.stemcr.2022.09.001

SUMMARY

Combining multiple Parkinson's disease (PD) relevant cellular phenotypes might increase the accuracy of midbrain dopaminergic neuron (mDAN) *in vitro* models. We differentiated patient-derived induced pluripotent stem cells (iPSCs) with a LRRK2 G2019S mutation, isogenic control, and genetically unrelated iPSCs into mDANs. Using automated fluorescence microscopy in 384-well-plate format, we identified elevated levels of α-synuclein (αSyn) and serine 129 phosphorylation, reduced dendritic complexity, and mitochondrial dysfunction. Next, we measured additional image-based phenotypes and used machine learning (ML) to accurately classify mDANs according to their genotype. Additionally, we show that chemical compound treatments, targeting LRRK2 kinase activity or αSyn levels, are detectable when using ML classification based on multiple image-based phenotypes. We validated our approach using a second isogenic patient-derived *SNCA* gene triplication mDAN model which overexpresses αSyn. This phenotyping and classification strategy improves the practical exploitability of mDANs for disease modeling and the identification of novel LRRK2-associated drug targets.

INTRODUCTION

Parkinson's disease (PD) is a heterogeneous movement disorder with a combination of motor and non-motor features caused by environmental and genetic risk factors or mutations in specific genes. Pathological characteristics of PD include the progressive loss of midbrain dopaminergic neurons (mDANs) and often the appearance of Lewy bodies, cytoplasmic inclusions containing aggregated α-synuclein (αSyn) protein (Blesa et al., 2022; Poewe et al., 2017).

Mutations in the leucine-rich repeat kinase 2 gene (*LRRK2*) have been associated with PD. The glycine to serine substitution at position 2019 (G2019S) in the LRRK2 kinase domain increases its activity and is assumed to be one reason for mDAN loss (Smith et al., 2006; West et al., 2005; Weykopf et al., 2019). One hypothesis is that LRRK2 G2019S causes defects in mitochondrial biology. Increased autophagy markers, but also PINK1/Parkin-, and Miro1-related defects support the idea that specifically mitophagy-linked processes are disturbed in LRRK2 G2019S neurons (Bonello et al., 2019; Hsieh et al., 2016; Schwab et al., 2017). Additionally, LRRK2 G2019S could induce mDAN loss by increasing the levels of phosphorylated αSyn, leading to its aggregation, since LRRK2 kinase inhibition can prevent phosphorylated αSyn from forming protein inclusions (Daher et al., 2014; Longo et al., 2017; Obergasteiger et al., 2020; Volpicelli-Daley et al., 2016; Xiong et al., 2017).

An emerging picture of PD is therefore that multiple disease mechanisms act together or can even exacerbate each other. Human patient induced pluripotent stem cell (iPSC)-derived mDANs expressing LRRK2 G2019S constitute a valuable *in vitro* model to understand PD. Despite the apparent value of neuronal models, important challenges remain: individual *in vitro* PD pathological features are often subtle or variable when examined across different differentiation batches or genotypes. Furthermore, single isolated PD phenotypes do not capture the multifactorial complexity of PD. Additionally, and despite their relevance physiologically, iPSC neuronal models are rarely used for PD-related drug discovery due to throughput feasibility concerns based on technical complexity as well as genetic variability (Cobb et al., 2018; Elitt et al., 2018). The goal of this study was therefore to develop a robust methodology able to detect multiple cellular PD-related pathophysiological phenotypes in a physiologically relevant human mDAN model system. We aimed for sufficient sensitivity to detect phenotypic variations based on genetic, but also chemical compound-induced phenotypic changes.

We demonstrate that multiple PD-relevant cellular phenotypes can be detected in microscopic images obtained from human patient LRRK2 G2019S iPSC-derived mDANs

This is an open access article under the CC BY license (http://creativecommons.org/licenses/by/4.0/).

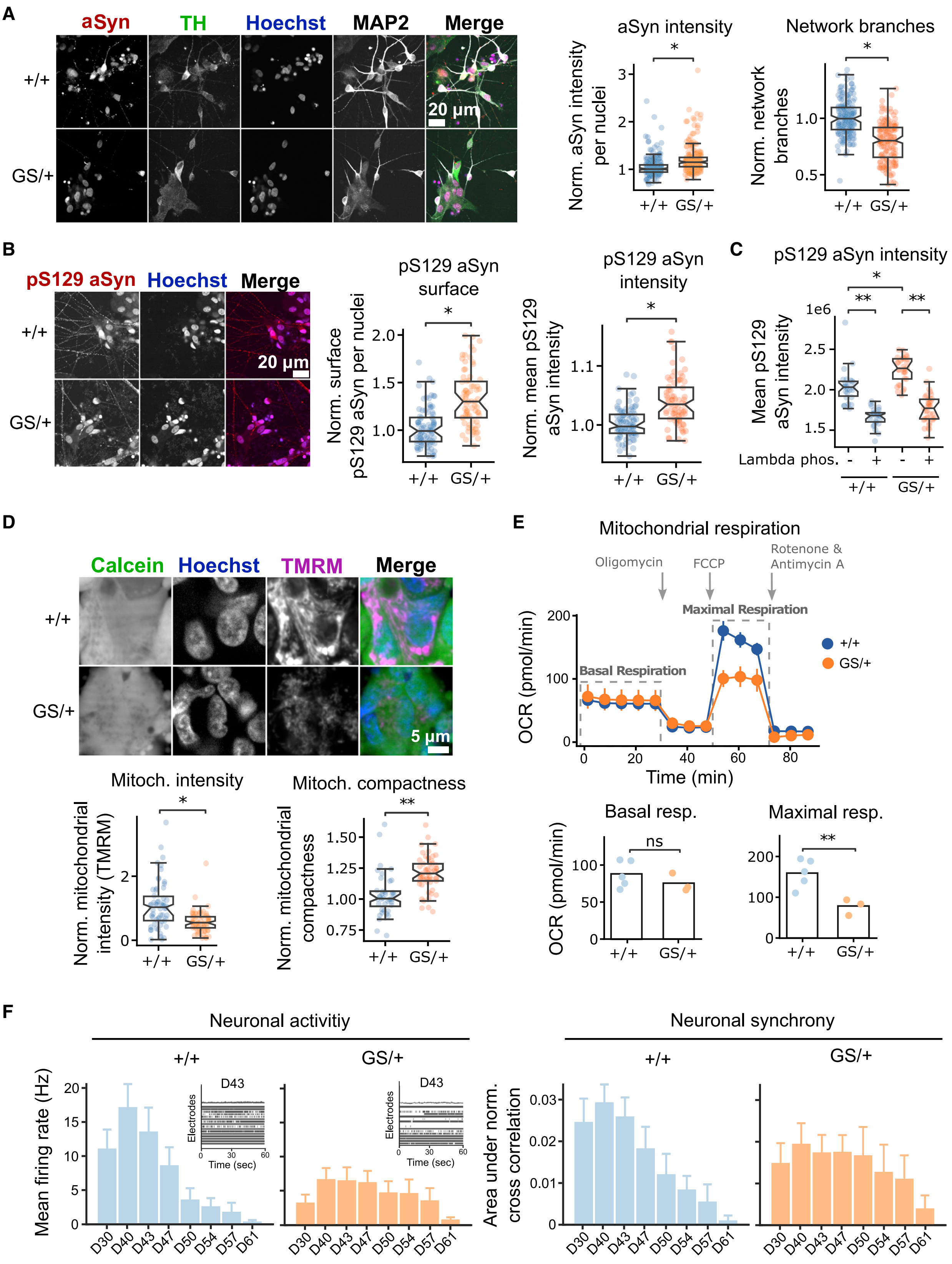

(legend on next page)

in 384-well-plate format. We show that machine learning (ML) can be used to distinctively classify PD from control mDANs based on multiple image-derived phenotypes. Finally, we demonstrate that our multi-phenotype classification approach is sensitive enough to detect different small molecules with a PD-relevant mode of action. Our work outlines a novel strategy to use iPSC-derived mDANs from LRRK2 or SNCA mutation carriers for imaging-based disease modeling by computationally combining multiple disease-relevant phenotypes.

RESULTS

LRRK2 G2019S mDANs display multiple hallmarks of PD

iPSC lines from a patient with a confirmed LRRK2 G2019S mutation and an isogenic control line (GS/+ and +/+, respectively) were differentiated into mDANs expressing neuronal markers (TUBB3 and MAP2) and dopaminergic neuron markers, including tyrosine hydroxylase (TH) in combination with expression of FOXA2, while the glial marker glial fibrillary acidic protein was only weakly expressed (Figures S1A and S1B). Immunostaining showed similar percentages of TH- and MAP2-expressing isogenic control +/+ and GS/+ neurons indicating comparable differentiation potentials in both genotypes (Figure S1C).

To detect image-based hallmarks of PD, we designed an immunofluorescence-based workflow in 384-well-plate format. Cryopreserved 30-day-old mDANs were seeded in 384-well plates, cultured for 7 days, fixed, and stained. Automated microscopy and image segmentation was used to extract multiple quantitative image features. First, GS/+ and +/+ mDANs were stained with antibodies against αSyn, TH, and MAP2. In the TH-positive GS/+ neuronal population, αSyn levels were increased by 15% (Figure 1A). Western blotting with a different antibody confirmed the increase in αSyn levels across multiple differentiation batches (Figures S1F–S1I). In addition, MAP2 staining indicated that fewer dendritic branches were present in GS/+ neurons (Figure 1A). Staining with a pS129 αSyn antibody showed that the surface area occupied by pS129 αSyn and its fluorescence intensity were increased (Figure 1B). Together, both observations indicate that accumulation as well as increased phosphorylation are occurring. To exclude signal originating from non-phosphorylated forms of αSyn, we treated the fixed cells with lambda phosphatase. Lambda phosphatase treatment strongly reduced pS129 αSyn signal intensity in both GS/+ and +/+ neurons, indicating that pS129 αSyn levels are indeed increased in GS/+ mDANs (Figure 1C).

Staining with the live cell dye calcein and the mitochondrial membrane potential-sensitive dye tetramethylrhodamine (TMRM) indicated that the overall TMRM fluorescence in living cells was decreased by 33% in GS/+ mDANs suggesting that the intactness of the mitochondrial membrane is compromised in GS/+ mDANs. Additionally, mitochondria in GS/+ mDANs were more compact (Figure 1D). We used the mitochondria-targeting toxin rotenone to validate the TMRM staining (Figure S2). Additionally, we measured the oxygen consumption rate. The basal respiration rate did not differ between both genotypes, while the maximal respiration rate after carbonyl cyanide p-(tri-flurometho xy)phenyl-hydrazone (FCCP) treatment was 2-fold increased in +/+ controls (Figure 1E). To test the mDANs electrophysiological activity, we generated multi-electrode array (MEA) recordings from differentiation D30 to D61. We found that the mean action potential firing rate in GS/+ neurons was less than half compared with +/+ neurons. Additionally, inter-neuronal synchrony was less pronounced in GS/+ neurons, indicating potential synaptic defects (Figure 1F). Taken together, we found that LRRK2 G2019S mDA neurons show multiple hallmarks of PD rendering them useful as a disease model.

Figure 1. LRRK2 G2019S mDANs overexpress αSyn and display mitochondrial dysfunction

(A) iPSC-derived LRRK2 G2019S mDANs were immunostained against αSyn, TH, and MAP2 and αSyn intensity in TH-positive GS/+ neurons as well as a neuronal network complexity was quantified in microscopic images.

(B) Immunofluorescence staining against pS129 αSyn, αSyn, and MAP2 and quantification of pS129 αSyn in neurites as well as overall pS129 αSyn fluorescence intensity.

(C) mDANs were treated with lambda phosphatase before staining with a pS129 αSyn antibody.

(D) Staining with the live cell dye calcein and mitochondrial membrane potential-sensitive dye TMRM and quantification of TMRM intensity and mitochondrial (TMRM) compactness.

(E) Assessment of mitochondrial respiration using the Seahorse XF analyzer. Seahorse experiments were performed in triplicate, and means ± SEMs are shown. Imaging experiments shown in panels (B)–(D) were performed at least in duplicate with multiple technical replicates. Each data point represents one well. All data have been median normalized to the respective +/+ condition per plate. Welch's unequal variances t test was used for significance testing. Notches in boxplots indicate the 95% confidence interval.

(F) Multi-electrode array (MEA) recordings of +/+ and GS/+ neuronal population between differentiation D30 and D61. Neuronal activity was determined by measuring action potentials per second on single electrodes. Inserts show sample D43 activity traces from multiple electrodes in one well (left panel). Synchrony between neurons was determined by measuring simultaneous action potentials across multiple electrodes (right panel).

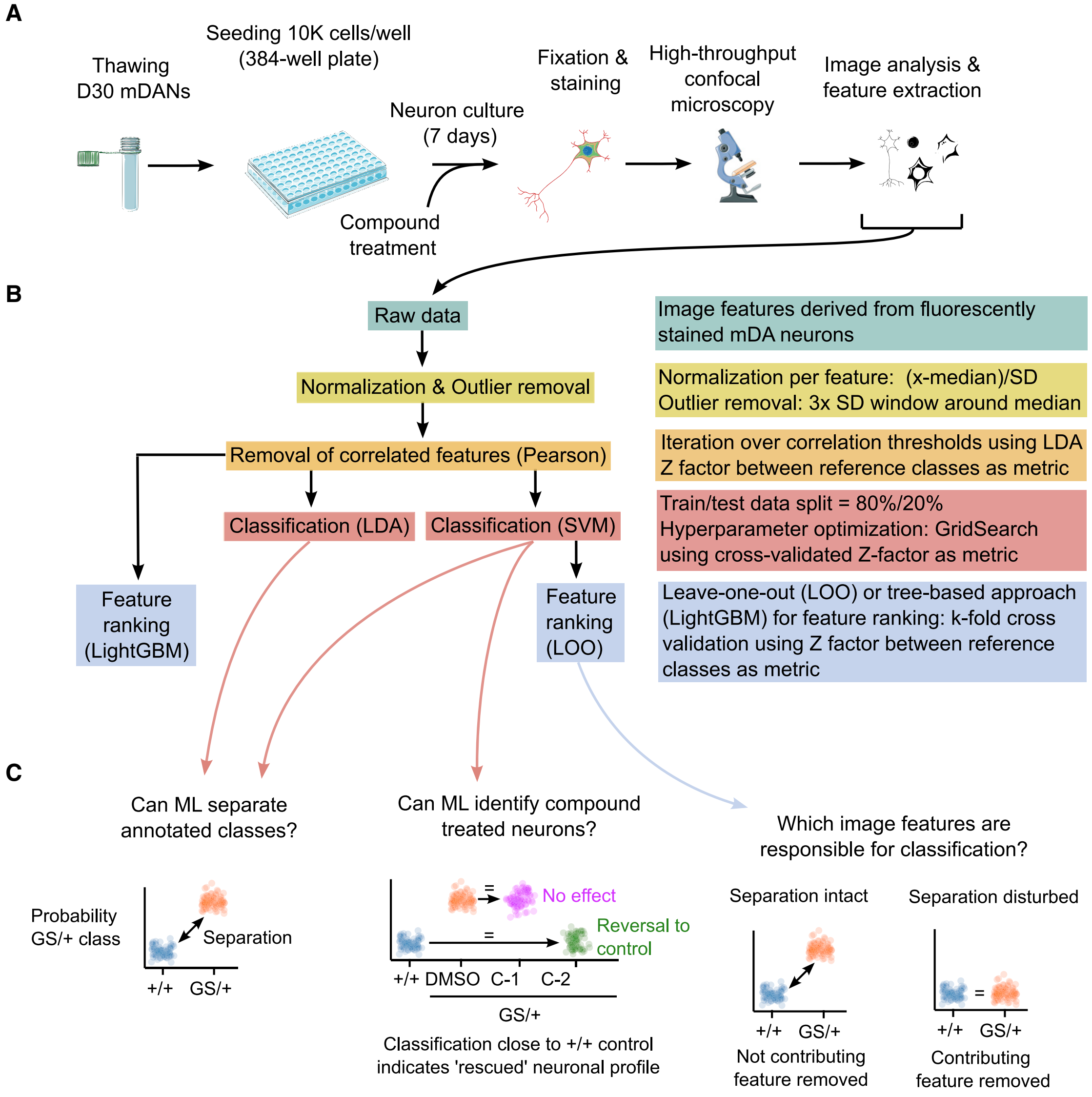

Figure 2. Machine learning (ML) strategy to classify neurons based on image-derived cellular features

(A) Schematic depiction of the generation of image-derived cellular feature data.

(B) Overview of the data processing steps and ML methodology.

(C) Schematic depiction of how ML classification was used to separate different neuronal cell lines (left panel), identify bioactive chemical compounds (middle panel), and how "leave-one-out" analysis can identify the contribution of individual image-derived cellular features to ML classification (right panel).

ML classification can distinguish neuronal genotypes based on image-derived cellular features

We hypothesized that the combination of multiple image-based phenotypes would give rise to a "neuronal fingerprint" or "profile" and allow the accurate and robust identification of different cell lines or treatment conditions, thereby making it a useful tool for iPSC-based disease modeling or compound screening. We applied different ML algorithms termed "classifiers" to achieve this task. Specifically, we used linear discriminant analysis (LDA) (Fisher, 1936), support vector machine (SVM) (Cortes and Vapnik, 1995), and light gradient boosting machine (LightGBM) (Ke et al., 2017) algorithms (Figure 2). Quantitative image-derived features were used as input data, and the ML classifiers were trained to separate two classes from each other (Tables S1 and S2). Next, additional classes could be mapped to the pre-trained reference classes. For example, to estimate the effect of a chemical compound treatment, compound-treated cells can be classified in comparison to DMSO-treated mutant and wild-type cells. An increased

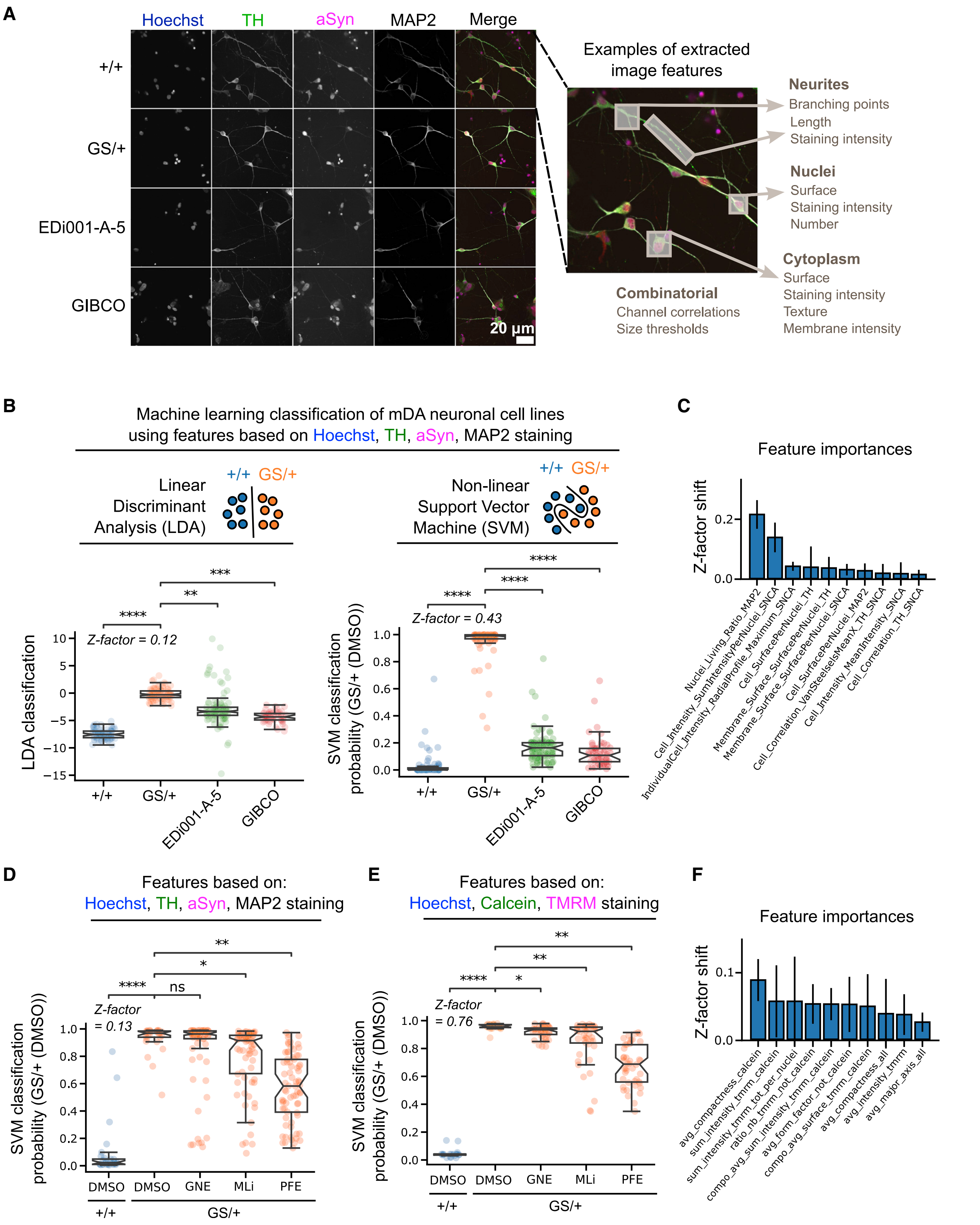

(legend on next page)

classification proximity indicates a higher phenotypic similarity (Figure 2C).

Using these ML classifiers, we tested whether PD-specific phenotypic neuronal fingerprints might exist. To evaluate the specificity of a GS/+ phenotypic fingerprint compared with the +/+ isogenic control line, we differentiated two additional and genetically unrelated control iPSC lines (EDi001-A-5 and GIBCO) into mDANs (Figure S3). All four mDAN cell lines were then stained with Hoechst and antibodies against αSyn, TH, and MAP2. We derived a total of 126 quantitative features from the images (Figure 3A, Table S1). 54 image features were TH+ cell type specific (43%) and specifically represented mDAN biology. We hypothesized that a weighted combination of all 126 cellular image features might allow the generation of a unique phenotypic fingerprint per cell line. Secondly, we hypothesized that the generated phenotypic fingerprint of GS/+ neurons would be significantly different from all control lines.

To test our hypotheses, we evaluated the two supervised ML classifiers LDA and SVM. In a first step, we determined the Pearson correlations of all image features to remove strongly correlated image features. Both LDA and SVM algorithms were then trained repeatedly on shuffled sets of 80% of the imaging data and tested on 20% of the imaging data. In total, training and testing were repeated 25 times on shuffled slices of the dataset in a process referred to as cross-validation (CV) (Figure 2B). CV is useful to detect and prevent overfitting and to increase robustness since the ML models are trained on multiple slightly different datasets. We observed that training variability over all cycles was generally low, indicating that sufficient training data were provided to both the LDA and SVM algorithms. Alternatively, we used data from one plate for model training, evaluated the model on two unseen plates, and obtained similar results (Figure S5A).

Although by eye the four mDAN lines appeared similar (Figure 3A), both LDA and SVM classification algorithms distinguished GS/+ neurons from control cell lines. Overall, control cell lines appeared more similar compared to each other than the GS/+ neurons. Next, we calculated the Z-factor between the GS/+ and +/+ neuronal classifications (Zhang et al., 1999). The SVM classification Z-factor was superior to the LDA Z-factor (0.12 versus 0.43) (Figure 3B). Based on these results, we focused mainly on SVM classification. To obtain a biological meaningful explanation of the classification results, we applied leave-one-out cross-validation (LOOCV). During LOOCV, each image feature is left out once, classification is performed repeatedly on the remaining image features, and the resulting Z-factor is calculated (Figure 2C). LOOCV demonstrated that our SVM results can most likely be explained by cell line differences concerning the ratio of MAP2-positive neurons and the level of αSyn (Figure 3C). To test whether strong, but more general cellular stress responses such as protein folding or reactive oxygen species induced stress could mimic the effects of a G2019S mutation, neurons were treated with tunicamycin or sodium arsenite ($NaAsO_2$). Despite the presence of the stressors, LDA and SVM analysis did not detect any change in +/+ control neuron classification (Figure S6). Together these findings demonstrate that iPSC-derived mDANs can be classified based on a genetic mutation and image-extracted phenotypes.

ML classification identifies LRRK2 inhibitor-treated neurons based on image-derived cellular features

Next, we asked whether SVM-driven analysis can detect chemical compound-induced phenotypic changes. We hypothesized that LRRK2 inhibitor treatment might partially rescue the previously observed combined feature phenotype (Figure 3B). Cryopreserved D30 mDANs were seeded in 384-well plates. 6 days after seeding the LRRK2 inhibitors GNE-7915, PFE-360, and MLi-2 were added for 24 h, and the neurons were fixed and stained using Hoechst, αSyn, TH, and MAP2 antibodies. Image-based feature extraction, data processing, and SVM model training were performed.

Figure 3. Machine learning (ML) classification can identify genotype-related and chemical compound-induced phenotypic differences based on image-derived cellular features in mDANs

(A) Representative images of neurons stained with Hoechst and antibodies against TH, αSyn, and MAP2. Image-derived cellular features were extracted from such images.

(B) The two supervised ML classification algorithms linear discriminant analysis (LDA) and support vector machine (SVM) were trained to separate the two reference classes GS/+ and +/+ isogenic control mDANs. The additional mDAN control lines were then mapped to the reference classes' feature space.

(C) Leave-one-out cross-validation (LOOCV) to identify individual feature contributions to SVM classification of multiple cell lines in (B).

(D) SVM classification of GS/+ and +/+ isogenic control mDANs and mapping of neurons treated with the LRRK2 inhibitors GNE-7915, MLi-2, and PFE-360 to the reference classes' feature space.

(E) Same experiment as in (D) but instead neurons were stained with Hoechst, tetramethylrhodamine (TMRM), and calcein.

(F) LOOCV to identify individual feature contributions to SVM classification in (E). All imaging data were generated in duplicate experiments with multiple technical replicates. Each data point represents one well. Mann-Whitney U-testing was performed for significance testing. Notches in boxplots indicate the 95% confidence interval.

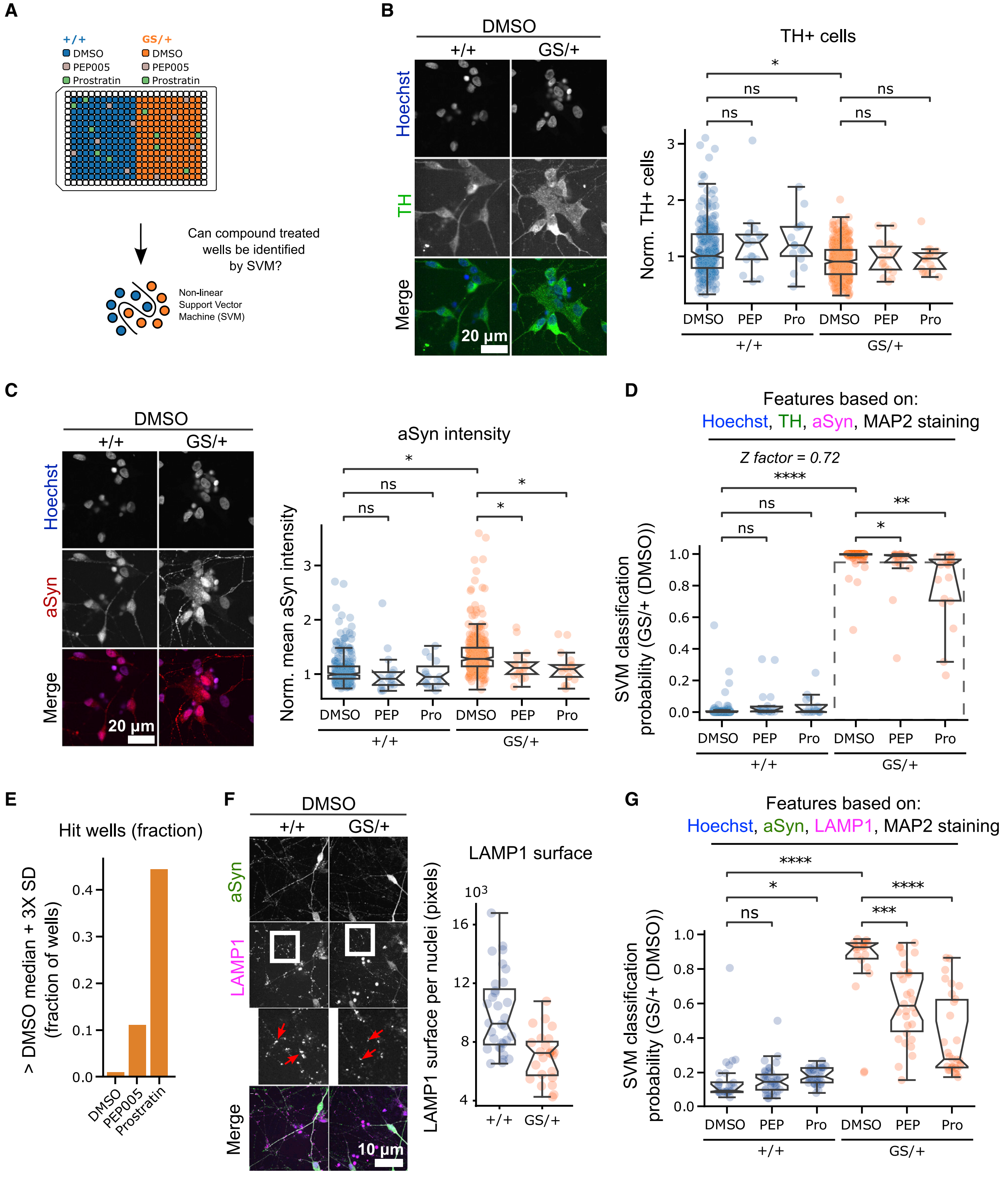

Figure 4. Machine learning (ML) can identify PKC agonist-treated LRRK2 G2019S mDANs in a simulated screening setup

(A) Schematic depiction of experimental design. Single wells spiked with PEP005 or prostratin were randomly distributed over the plate. Support vector machine (SVM) classification was applied to identify these wells.

(B) and (C) Representative images illustrate PEP005 and prostratin effects on the number of TH-positive cells and αSyn staining intensity.

(legend continued on next page)

The SVM classifier successfully distinguished +/+ and GS/+ mDANs treated with DMSO with a Z-factor of 0.13 (Figure 3D). Next, LRRK2 inhibitor-treated GS/+ mDANs were classified relative to the DMSO controls. GNE-7915 did not lead to phenotypic changes detectable in our assays and resembled the DMSO control classification. PFE-360 and MLi-2 induced subtle phenotypic differences detected by Hoechst/αSyn/TH/MAP2 staining and were classified as significantly different from DMSO-treated neurons. The shift toward the +/+ isogenic control was strongest for the PFE-360 treated GS/+ mDANs (Figure 3D). Additionally, we tested all three inhibitors in a second patient-derived PD mDAN model containing an SNCA gene triplication (Devine et al., 2011; Gwinn et al., 2011). We observed only modest GNE-7915 phenotypic effects and no detectable response to PFE-360 or MLi-2 (Figure S4). LRRK2 kinase inhibitors therefore seem to induce stronger phenotypic changes in LRRK2 mutated neurons than in SNCA triplication neurons.

Next, we tested whether LRRK2 inhibitor treatment would also lead to SVM-detectable changes on the mitochondrial level. Neurons were cultured and treated as before and stained with Hoechst, the live cell dye calcein, and the mitochondria-specific dye TMRM. 96 image features were calculated based on these three stainings (Table S2), and an SVM model was trained to distinguish +/+ from GS/+ mDANs. We then applied the SVM model to sets of mitochondrial image features from LRRK2 inhibitor-treated mDANs. Similar to the previous results obtained with the Hoechst/αSyn/TH/MAP2 staining, we detected only a weak effect of GNE-7915 on the measured mitochondrial phenotypes, while PFE-360 and MLi-2 treatment of GS/+ mDANs led to a classification shift toward +/+ control mDANs (Figure 3E). To identify the mitochondrial features most responsible for the observed classification result, we performed LOOCV analysis. We found that mitochondrial shape (i.e., compactness and form factor) as well as TMRM intensity contributed the most to the classification result (Figure 3F).

Detection protein kinase C (PKC) agonist-treated single wells using multiple image-derived cellular features in LRRK2 G2019S neurons

Recently, Laperle et al. demonstrated that lysosomal activation by phorbol esters, such as PEP005 and prostratin, reduced αSyn levels in iPSC-derived mDANs (Laperle et al., 2020). Given the established connection between LRRK2 and lysosomal biology, we hypothesized that PEP005 and prostratin might also be able to lower the elevated αSyn levels in our LRRK2 G2019S model and thereby shift multiple cellular phenotypes toward a control phenotype (Hockey et al., 2015; Obergasteiger et al., 2020). To demonstrate that ML classification can detect a chemical modulation in mDANs, we treated six randomly selected wells per plate with PEP005 or prostratin for 72 h (Figure 4A).

Next, cells were fixed and stained with Hoechst and αSyn, TH, and MAP2 antibodies, and 126 image features were extracted (Table S1). Verification of individual image features, such as the number of TH+ cells, showed that PEP005 and prostratin compound treatments were not toxic for either GS/+ nor +/+ neurons (Figure 4B). PEP005 and prostratin treatments led to a decrease in αSyn levels, specifically in GS/+ neurons, but not control +/+ neurons, confirming the initial results of Laperle et al. obtained in different PD mDAN lines (Figure 4C). Next, we trained an SVM model to distinguish +/+ from GS/+ mDANs using image-based features as input. Consistent with our previous results, SVM was able to separate both DMSO-treated control classes +/+ and GS/+ with high accuracy (0.98 ± SEM 0.02) and a Z-factor of 0.72 (Figure 4D). We then applied the SVM model to sets of image features originating from PEP005- and prostratin-treated wells. Compound-treated GS/+ neurons classified differently than the DMSO-treated GS/+ neurons. Although this effect was small for PEP005, most prostratin-treated wells shifted toward the +/+ isogenic control neurons. Additionally, we observed that +/+ control neurons responded less to PEP005 and prostratin treatment (Figure 4D).

To assess whether single PEP005- or prostratin-treated wells could be detected in a typical screen setup using only a small number of replicates, we determined a 3x standard deviation (SD) threshold around the median of the DMSO-treated GS/+ neurons. We calculated the percentage of compound-treated wells beyond the threshold that could be regarded as a hit. For GS/+ neurons treated with DMSO, less than 1% of wells were more than 3 SDs away from the median, while this was 11% of PEP005- and 43% of prostratin-treated wells (Figure 4E). To estimate

(D) SVM classification of GS/+ and +/+ isogenic control mDANs based on cellular image features extracted from Hoechst, αSyn, TH, and MAP2 staining. PEP005- and prostratin-treated wells were then mapped to the reference classes' feature space. The broken square includes datapoints (wells) that are at a distance of more than three standard deviations (SDs) from the GS/+ DMSO-treated median.
(E) Quantification of the fraction of wells more than three SDs from the GS/+ DMSO-treated class median.
(F) Representative images illustrate LAMP1 staining and differences in surface area.
(G) SVM classification of treated and untreated GS/+ and +/+ isogenic control mDANs based on cellular image features extracted from Hoechst, αSyn, LAMP1, and MAP2 staining. All imaging data were generated in triplicate experiments with multiple technical replicates. Each data point represents one well. Mann-Whitney U-testing was performed for significance testing. Notches in boxplots indicate the 95% confidence interval.

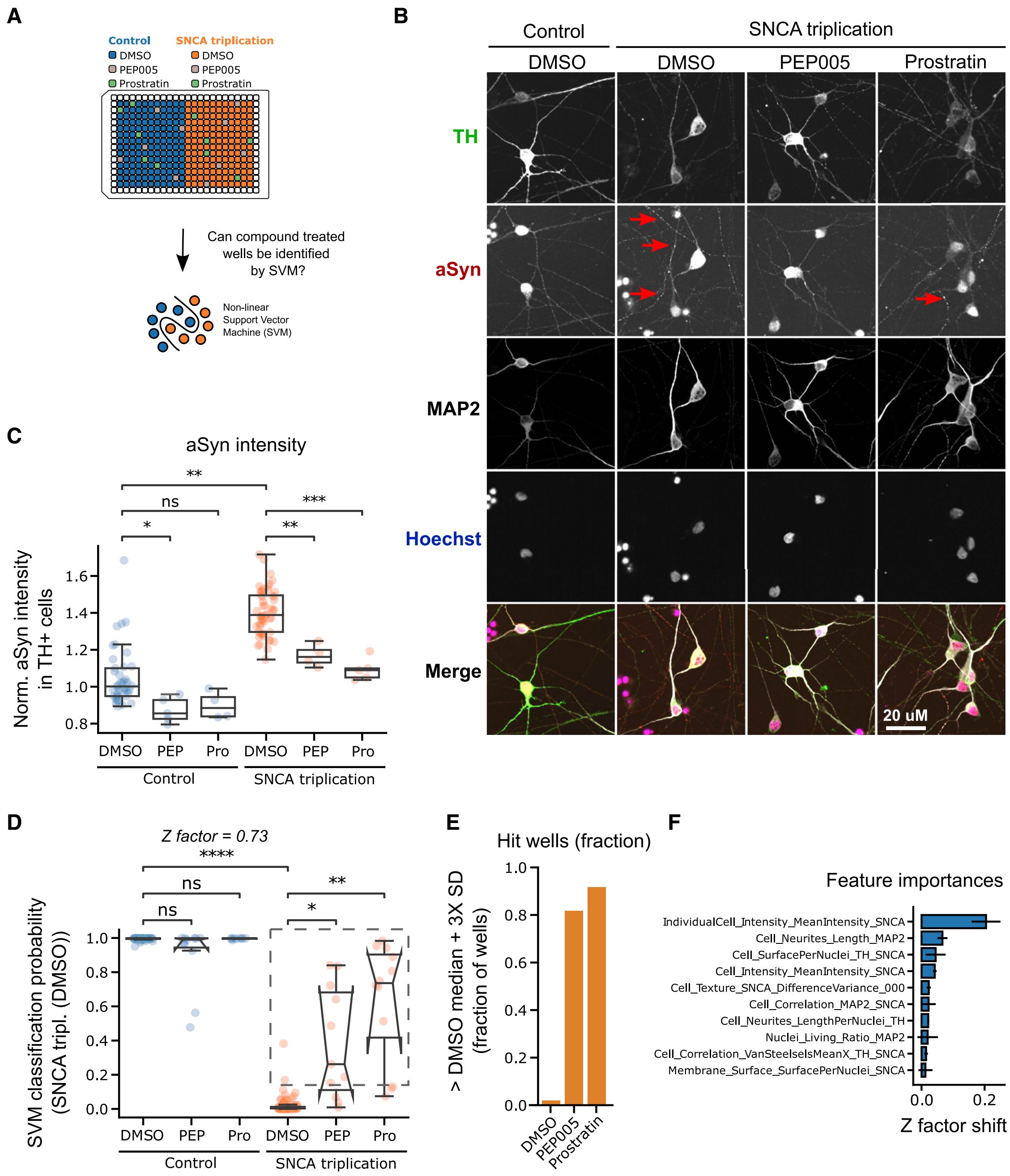

Figure 5. Machine learning (ML) can identify protein kinase C (PKC) agonist-treated SNCA triplication mDANs in a simulated screening setup

(A) Schematic depiction of experimental design. Single wells spiked with PEP005 or prostratin were randomly distributed over the plate. Support vector machine (SVM) classification was applied to identify these wells.

(B) Representative images of neurons stained with Hoechst and TH, αSyn, and MAP2 antibodies after 37 days of differentiation and treated with either DMSO, PEP005, or prostratin. Red arrows indicate αSyn staining in neurites.

(C) Quantification of αSyn staining intensity across all treatment conditions.

(D) SVM classification of SNCA triplication and isogenic control mDANs based on cellular image features extracted from Hoechst, αSyn, TH, and MAP2 staining. PEP005- and prostratin-treated wells were then mapped to the reference classes' feature space. The broken square includes datapoints (wells) that are at a distance of more than three standard deviations (SDs) from the SNCA triplication DMSO-treated median.

(legend continued on next page)

the minimum number of wells required to determine whether a compound is active in a screening setup using neuronal profiles, we performed a calculation based on a typical GS/+ (DMSO) data distribution and measured how many replicate wells would be needed to detect at least one hit at least 3 SDs from the median. The calculation was repeated with different anticipated effect sizes and desired statistical power thresholds (Figures S5B–S5C). For example, given an effect size of 2 and a desired power of 0.85 to detect a deviation from the null hypothesis requires at least four replicates.

Since both LRRK2 as well as PEP005 and prostratin have been linked to lysosomal biology, we additionally investigated whether our assay would show improved compound detection using a lysosome-specific stain. A lysosome-associated membrane protein 1 (LAMP1) antibody was used to detect lysosomal image features. Several were altered in GS/+ neurons, such as decreased LAMP1 signal surface (Figure 4F). Treatment with PEP005 and prostratin and using multiple LAMP1 image features during SVM classification resulted in a larger shift of compound-treated GS/+ neuron-containing wells toward +/+ control neurons compared with lysosome-unspecific staining (Figures 4D and 4G).

Detection PKC agonist-treated single wells using multiple image-derived cellular features in SNCA triplication neurons

To generalize our neuronal profiling approach, we established a second PD mDAN model based on SNCA gene triplication-carrying donor iPSCs expressing four copies of SNCA and an isogenic control (Figure S3). Using both cell lines, we performed a similar experiment as described in Figure 4A with the aim to detect individual wells treated with PEP005 or prostratin using SVM classification (Figure 5A). SNCA triplication mDANs showed signs of αSyn accumulation in dendrites and a reduced dendritic network (Figure 5B). Image feature quantification confirmed that indeed αSyn levels were increased in SNCA triplication mDANs. Additionally, we observed αSyn lowering of 15% by PEP005 and 25% by prostratin (Figure 5C). Next, we trained an SVM classifier to separate isogenic control from SNCA triplication mDANs. Similar to the LRRK2 model, the SVM algorithm was able to separate isogenic control from SNCA triplication mDANs with high accuracy (0.97 ± SEM 0.03) resulting in a Z-factor of 0.73 (Figure 5D). SVM classification of SNCA triplication mDANs treated with PEP005 or prostratin showed a shift toward isogenic control mDANs. Isogenic control neurons treated with both compounds had a similar image feature-based profile and were statistically indistinguishable from DMSO-treated control neurons, suggesting a specific effect of PEP005 and prostratin in SNCA triplication neurons (Figure 5D).

In SNCA triplication neurons treated with DMSO, less than 1% of wells were more than 3 SDs away from the median, while this was 81% of PEP005- and 91% of prostratin-treated wells (Figure 5E). The single most important image feature distinguishing SNCA cell lines was the αSyn staining intensity, a proxy for cellular αSyn content explaining 0.2 points of the observed 0.73 Z-factor (Figure 5F). We confirmed the contribution of αSyn content and other features by using LightGBM, a different classification algorithm. These findings in a second PD-relevant disease model indicate that bioactive molecules such as PEP005 and prostratin can be detected using neuronal profiles and a small number of technical replicates.

mDAN characterization at differentiation D50 reveals altered image feature profiles compared with D37 neurons

To test whether multi-feature neuronal profiling could be applied to more mature neurons, we repeated classification experiments with 50-day-old SNCA and LRRK2 neurons and compared the results to 37-day-old neurons (Figures 6A and 6B). Levels of αSyn or TH were increased in D50 neurons, indicating increased maturity (Figure 6C). To test whether neuronal profiles were different between both time points, we classified D50 data using an SVM classification model developed with D37 data and found no significant differences, although some shifts were visible (Figure 6D). Unsupervised classification using principal component analysis (PCA) or PaCMAP (Wang et al., 2021) was able to separate all timepoints and genotypes from each other highlighting that detectable phenotypic alterations of neuronal profiles exist after prolonged maturation (Figure 6E).

DISCUSSION

In this study, we demonstrate that image-derived phenotypes in human iPSC-derived mDANs can be used for cell line stratification and the identification of chemical compound-treated neurons by ML classification approaches. iPSC-derived neurons are only rarely used in drug discovery

(E) Quantification of the fraction of wells at a distance of more than three SDs from the SNCA triplication DMSO-treated class median.
(F) Leave-one-out cross-validation (LOOCV) to identify individual feature contributions to SVM classification in (D). All imaging data were generated in triplicate experiments with multiple technical replicates. Each data point represents one well. Mann-Whitney U-testing was performed for significance testing. Notches in boxplots indicate the 95% confidence interval.

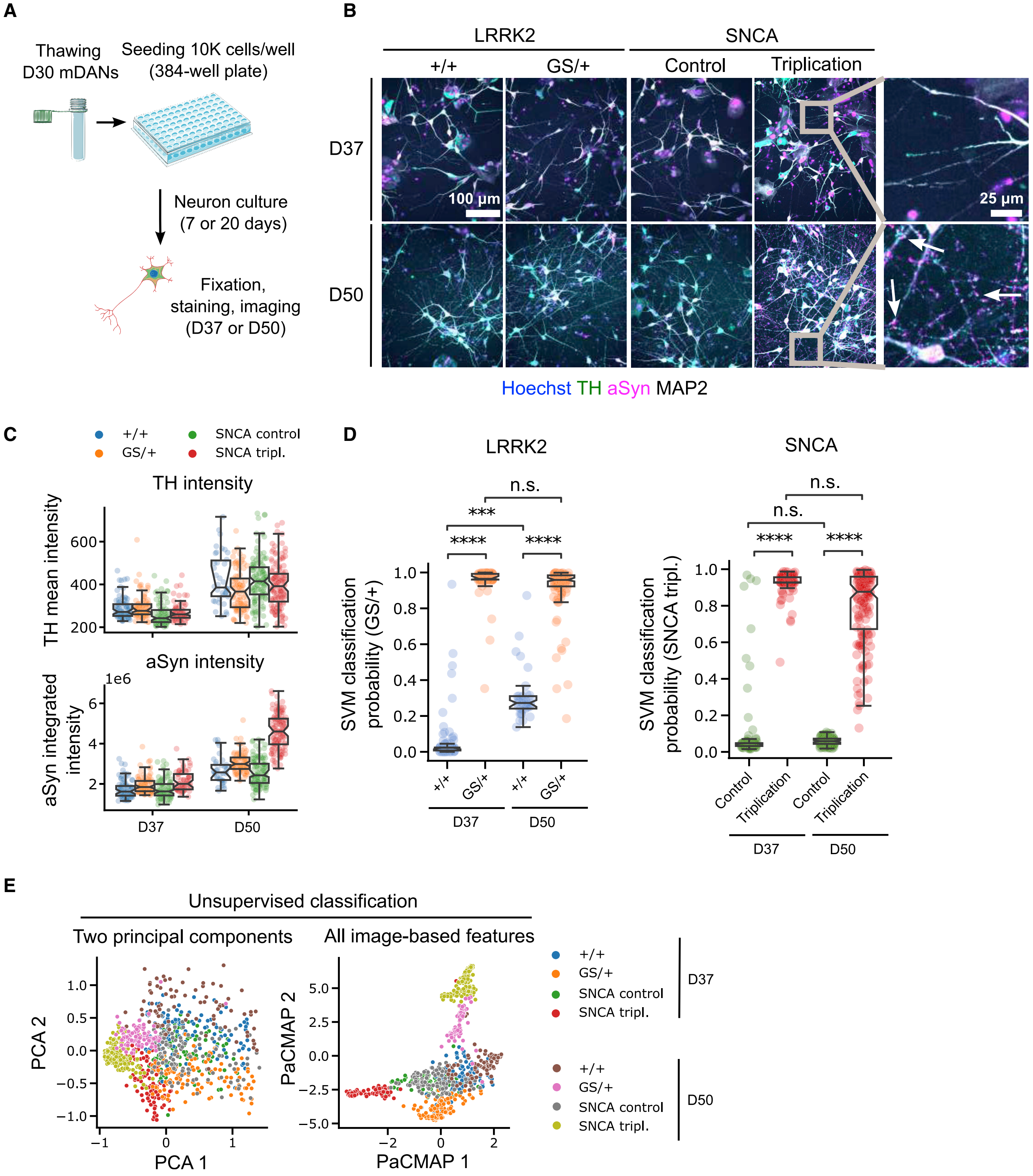

Figure 6. Imaging-based LRRK2 G2019S and SNCA triplication mDAN characterization at differentiation D50 reveals altered image feature profiles compared with D37 neurons

(A) Schematic depiction of experimental design.

(B) Representative images of neuronal cultures at differentiation D37 and D50. Arrows indicate clusters of αSyn staining signal.

(C) Quantification of the TH and αSyn staining signal at D37 and D50.

(legend continued on next page)

due to complex cell culture protocols, long culture duration, and genetic or clonal heterogeneity (Cobb et al., 2018; Elitt et al., 2018). We applied multiple strategies to improve the reproducibility of our iPSC-derived neuron models. First, we worked with large, cryopreserved batches to reduce the number of required differentiations and used isogenic controls to reduce sources of inter-donor genetic variability. Additionally, we developed a compact and automated 7-day experimental protocol in 384-well-plate format to reduce intervention steps and technical variability.

The functions of LRRK2 are not fully understood, but it has become clear that LRRK2 can trigger autophosphorylation at Ser1292 and phosphorylate a subset of Rab small GTPases (Rab8A and Rab10) (Rocha et al., 2022; Sheng et al., 2012; Steger et al., 2016). A direct readout of these targets was not present in our panel of stains. This is likely the reason why one of the three tested LRRK2 inhibitors showed only little effects in our experimental setup. Similarly, the used phorbol esters PEP005 and prostratin have specific phosphorylation-inducing effects on PKC subunits α and δ (Hampson et al., 2005; Laperle et al., 2020; Mischak et al., 1993), which we did not examine directly in our phenotypic characterization. We observed PEP005 and prostratin effects in both the LRRK2, but especially the SNCA triplication model, likely because both molecules have αSyn-lowering capabilities in mDANs (Laperle et al., 2020). Additionally, we show that both compounds can be identified with higher confidence if a LAMP1 stain is used to extract lysosome-related image features. Using a multi-phenotype strategy to characterize or detect chemical compounds therefore requires a compromise between the identification and reversal of general PD hallmarks or the focus on a specific mode of action related to a class of compounds.

Two previous studies described compound screening in iPSC-derived mDANs using resistance to rotenone-induced apoptosis and neurite outgrowth (Tabata et al., 2018) or resistance to carbonyl cyanide *m*-chlorophenylhydrazone-induced apoptosis and rescued mitophagy as readouts (Yamaguchi et al., 2020). In contrast to previous work, we used ML classification to bundle multiple phenotypes, which offers certain advantages: the used cellular stainings allow the extraction of many PD-relevant image features and thereby create a more biologically diverse representation of mDANs amendable to chemical interventions. Second, the combination of multiple, including subtle, phenotypes is statistically more robust than single phenotypic approaches. Additionally, our ML classification approach allows us to determine which phenotypic features contributed to the overall phenotypic differences between healthy and disease mDANs and might therefore aid the target deconvolution process.

Future research will need to evaluate whether only effects of monogenetic alterations, such as the mutations in LRRK2 or SNCA genes tested here, lead to distinguishable phenotypes or whether also idiopathic forms of PD, in which no single disease cause is known, have unique phenotypic profiles. Recent work using fibroblasts from 91 PD patients among which were 32 idiopathic PD cases indeed point into the direction that global phenotypic PD profiles might exist (Schiff et al., 2022). We anticipate that image-based multidimensional readouts capturing multiple PD-relevant phenotypes might increase the chance to detect active chemical compounds that rescue not only an isolated phenotype, but an ensemble of disease-relevant phenotypes.

EXPERIMENTAL PROCEDURES

Generation of iPSC lines and differentiation into mDANs

All iPSC lines were generated by third parties and are deposited in the European Bank for Induced Pluripotent Stem Cells (EBiSC, https://cells.ebisc.org/) and listed in the Human Pluripotent Stem Cell Registry (hPSCreg, https://hpscreg.eu/) (Table S3). The original generators have obtained the informed consent from the donors. iPSCs were cultivated on Geltrex-coated (Thermo Fisher Scientific) dishes in StemMACS iPS-Brew XF (Miltenyi Biotech). The medium was changed daily, and cells were passaged twice a week using 0.5 mM EDTA in PBS (Thermo Fisher Scientific). Mycoplasma testing was performed twice per month.

mDANs were differentiated using a modified protocol based on Kriks et al. (Kriks et al., 2011; Ryan et al., 2013; Weykopf et al., 2019). Briefly, iPSCs were seeded onto Geltrex-coated six-well plates or T75 flasks at a density of 2×10^5 cells/cm^2 in StemMACS iPS-Brew XF containing 10 μM Y-27632 (Hiss). The next day, medium was switched to KnockOut DMEM medium containing KnockOut serum replacement (both Thermo Fisher Scientific) supplemented with 200 nM LDN19318 (Axon Medchem) and 10 μM SB431542 (Biozol) for dual SMAD-inhibition. On day 2, also 100 ng/mL Shh C24II (Miltenyi Biotech), 2 μM Purmorphamine (Miltenyi Biotec), 100 ng/mL FGF8 (Peprotech), and 3 μM

(D) Support vector machine (SVM) classification of SNCA triplication and isogenic control mDANs based on cellular image features extracted from Hoechst, αSyn, TH, and MAP2 staining.
(E) Unsupervised classification of neuronal image feature profiles by principal component analysis (PCA) and pairwise controlled manifold approximation (PaCMAP). Imaging data were generated in triplicate experiments with multiple technical replicates per plate. Each data point represents one well. Mann-Whitney U-testing was performed for significance testing. Notches in boxplots indicate the 95% confidence interval.

CHIR99021 (Miltenyi Biotec) were added to the medium. After 5 days, medium was gradually shifted to Neurobasal medium (Thermo Fisher Scientific), and SB431542 was omitted from the medium. Starting at day 7, cells were grown only in the presence of LDN19318 and CHIR99021. On day 11, cells were switched to Neurobasal/B27/L-glutamine medium supplemented with CHIR99021 only. On day 13, cells were replated onto Geltrex-coated dishes in Neurobasal/B27/L-glutamine medium supplemented with 20 ng/mL BDNF, 20 ng/mL GDNF (both Cell Guidance Sys.), 221 μM L-ascorbic-acid (Sigma-Aldrich), 10 μM DAPT (Axon Medchem), 1 ng/mL TGF-βIII (Peprotech), 0.5 mM dibutyryl-cAMP (Enzo Life Sciences), and 10 μM Y-27632 (Hiss). Cells were maintained in the same medium but without Y-27632. Around day 23–25, cells were dissociated using StemPro Accutase (Thermo Fisher Scientific) and plated at a density of 1.4 x 105 cells/cm^2 onto Geltrex-coated dishes. To eliminate non-neuronal cells, cultures were treated with 1 μg/mL Mitomycin C for 2 h on day 26. At day 30, neuronal cultures were dissociated using StemPro Accutase supplemented with 10 μM Y-27632 and singularized. Cells were counted and cryopreserved at 2.5 × 10^6 cells/vial in CryoStor CS 10 (Sigma-Aldrich).

Neuronal culture and compound treatment

30 DIV (days *in vitro*)-old neurons were thawed and centrifuged (400 *g*, 5 min, RT) in basal medium (Table S3) supplemented with ROCK inhibitor (Tocris #1254). Cell pellets were resuspended in differentiation medium (Table S3) supplemented with ROCK inhibitor. 384-well plates (Perkin Elmer, #6007558) were coated with 15 μg/mL poly-L-ornithine for 1 h at 37°C followed by 10 μg/ml laminin overnight at 4°C. Using trypan blue (Sigma, #T8154-20ML) and a Countess automated cell counter (Invitrogen), 10 × 10^3 cells/well were seeded in 384-well plates. Edge wells were avoided for seeding and filled with PBS. Typically, thawed cells were incubated at 37°C and 5% CO_2 for 7 days until 37 DIV with differentiation medium changes every other day. Plate coating, cell seeding, and medium changes were automated using an Agilent Bravo pipetting robot (Agilent) and EL406 plate washer and dispenser (Biotek). Compound treatment with 1 μM PEP005 (Tocris, #4054) and 5 μM prostratin (Tocris, # 5739) was performed at 34 DIV for 72 h until 37 DIV. Compound treatment with 0.1 μM GNE-7915 (MedChemExpress, #HY-18163), 0.1 μM MLi-2 (MedChemExpress, #HY-100411), 0.1 μM PFE-360 (MedChemExpress, #HY-120085), and 0.1 μM rotenone (Sigma, #R8875) was performed at 36 DIV for 24 h until 37 DIV. Treatment with 2 μM tunicamycin (MedChemExpress, #HY-A0098) and 50 μM sodium arsenite (Sigma, #1062771000) was performed for 3 h on DIV 37. For western blotting experiments, 5 μM AraC (Sigma, #C6645) was added for 24 h before cell lysis on DIV 37 or 44.

In situ cytochemistry

Fixation was performed in 4% PFA (EMS Euromedex, #15710) for 20 min, followed by two PBS (Gibco, #14190) washes and permeabilization and blocking with 10% FBS (Gibco, #10270-106) and 0.1% Triton X-100 (Sigma, #T9284) dissolved in PBS for 1 h. Primary antibodies (Table S3) were prepared in antibody dilution buffer (PBS supplemented with 5% FBS and 0.1% Triton X-100) and incubated with the cells overnight at 4°C, followed by three PBS washes. Secondary antibodies and Hoechst (Table S3) in antibody dilution buffer were added to the cells for 1 h at RT, followed by three PBS washes. Mitochondrial imaging was performed in live cells. All dyes (Table S3) were prepared in differentiation medium and incubated with the cells for 30 min at 37°C and 5% CO_2, followed by a wash with differentiation medium. Cells were imaged in a preheated microscope chamber at 37°C and 5% CO_2. *In situ* cytochemistry was automated using an Agilent Bravo pipetting robot (Agilent) and EL406 plate washer and dispenser (Biotek).

Imaging and image analysis

Imaging was performed on a Yokogawa CV7000 microscope in scanning confocal mode using a dual Nipkow disk. 384-well plates (Perkin Elmer, #6007558) were mounted on a motorized stage and images were acquired in a row-wise “zigzag” fashion at RT for fixed cells and 37°C and 5% CO_2 for living cells. The system’s CellVoyager software and 405/488/561/640-nm solid laser lines were used to acquire single Z-plane 16-bit TIFF images through a dry 40× objective lens using a cooled sCMOS camera with 2,560 × 2,160 pixels and a pixel size of 6.5 μm without pixel binning. Nine images in a 3 × 3 orientation were acquired from the center of each well. Image segmentation and feature extraction was performed with an in-house software written in C++. Except for the detection of mitochondrial structures, image segmentation was performed on illumination-corrected raw images based on fluorescent channel intensity thresholds empirically determined per plate. Multiple quantitative image features were calculated (Tables S1 and S2). Mitochondrial structures and features were detected in rolling-ball background-subtracted and top-hat filtered images similar to a protocol described previously (Iannetti et al., 2016).

ML analysis

To support the reproducibility of the ML method of this study, the ML summary table is included in the supplemental information per data, optimization, model, and evaluation (DOME) recommendations (Walsh et al., 2021) (Table S4). Multiple datasets were generated differing in terms of the used mDANs, chemical compound treatment, and fluorescent staining (Table S5). Input data were normalized, outliers were removed, and the number of input features was reduced by removing strongly correlated features (Figure 2, Table S4). We applied the Python-written ML library scikit-learn to train and test all models (Pedregosa et al., 2011). We primarily used supervised binary classification algorithms. Figure 2B summarizes the overall ML workflow. All models’ hyperparameters were optimized using scikit-learn’s GridSearchCV module and evaluated using k-fold cross-validation. Performance was checked using accuracy. All raw data are listed in Table S5 and are available together with the corresponding Jupyter notebook ML pipelines on GitHub (https://github.com/johanneswilbertz/mDA-neuron-classification).

Statistics

All data were generated at least in duplicate with neurons from a single differentiation batch. All data are represented as boxplots. The notches of the box represent the 95% confidence interval of the median obtained by bootstrapping with parameter value 1,000. Each data point represents the mean of a single well of a

384-well plate comprised of nine images. Data from different plates were median normalized to allow comparison across plates acquired on different days. Data processing and plotting were carried out with Python packages Pandas (McKinney, 2010), Matplotlib (Hunter, 2007), and Seaborn (Waskom, 2021). Null hypothesis significance testing was performed with the freely available Python package Statannot (Weber, 2022). For data not displaying a normal distribution, the non-parametrical Mann-Whitney U-test was performed. For normally distributed data, Welch's t test was applied. Statistical significance is presented in the figures as $*p < 0.05$, $**p < 0.01$, $***p < 0.001$, $****p < 0.0001$, and not significant (ns = $p > 0.05$).

SUPPLEMENTAL INFORMATION

Supplemental information can be found online at https://doi.org/10.1016/j.stemcr.2022.09.001.

AUTHOR CONTRIBUTIONS

A.V. performed profiling experiments; L.C. developed and performed image analysis and data processing; Z.H. developed the ML pipeline; N.W.D. developed the image analysis software; B.W., M.S., and S.H. performed neuronal differentiation and quality controls; J.L. designed neuronal stainings; Y.J.K. initiated the study; M.P. and O.B. supervised differentiation experiments; A.O. supervised image analysis procedures; P.S. and L.B. co-supervised the study and designed experiments; J.H.W. co-supervised the study, designed experiments, conceptualized and performed image analysis and data processing, and wrote the manuscript with input from all authors.

ACKNOWLEDGMENTS

The authors thank Bruno Dos Santos (Luxembourg Center for Systems Biomedicine) for help with Seahorse experiments and the entire Ksilink team for input during project discussions. Figures were partly generated using Servier Medical Art licensed under a Creative Commons Attribution 3.0 license.

CONFLICT OF INTERESTS

O.B. is a co-founder and shareholder of LIFE & BRAIN GmbH and a member of Ksilink's board of directors.

Received: March 4, 2022
Revised: August 31, 2022
Accepted: September 1, 2022
Published: September 29, 2022

REFERENCES

Blesa, J., Foffani, G., Dehay, B., Bezard, E., and Obeso, J.A. (2022). Motor and non-motor circuit disturbances in early Parkinson disease: which happens first? Nat. Rev. Neurosci. *23*, 115–128. https://doi.org/10.1038/s41583-021-00542-9.

Bonello, F., Hassoun, S.-M., Mouton-Liger, F., Shin, Y.S., Muscat, A., Tesson, C., Lesage, S., Beart, P.M., Brice, A., Krupp, J., et al. (2019). LRRK2 impairs PINK1/Parkin-dependent mitophagy via its kinase activity: pathologic insights into Parkinson's disease. Hum. Mol. Genet. *28*, 1645–1660. https://doi.org/10.1093/hmg/ddz004.

Cobb, M.M., Ravisankar, A., Skibinski, G., and Finkbeiner, S. (2018). iPS cells in the study of PD molecular pathogenesis. Cell Tissue Res. *373*, 61–77. https://doi.org/10.1007/s00441-017-2749-y.

Cortes, C., and Vapnik, V. (1995). Support-vector networks. Mach. Learn. *20*, 273–297. https://doi.org/10.1007/BF00994018.

Daher, J.P.L., Volpicelli-Daley, L.A., Blackburn, J.P., Moehle, M.S., and West, A.B. (2014). Abrogation of α-synuclein–mediated dopaminergic neurodegeneration in LRRK2-deficient rats. Proc. Natl. Acad. Sci. USA *111*, 9289–9294. https://doi.org/10.1073/pnas.1403215111.

Devine, M.J., Ryten, M., Vodicka, P., Thomson, A.J., Burdon, T., Houlden, H., Cavaleri, F., Nagano, M., Drummond, N.J., Taanman, J.-W., et al. (2011). Parkinson's disease induced pluripotent stem cells with triplication of the α-synuclein locus. Nat. Commun. *2*, 440. https://doi.org/10.1038/ncomms1453.

Elitt, M.S., Barbar, L., and Tesar, P.J. (2018). Drug screening for human genetic diseases using iPSC models. Hum. Mol. Genet. *27*, R89–R98. https://doi.org/10.1093/hmg/ddy186.

Fisher, R.A. (1936). The use of multiple measurements in taxonomic problems. Ann. Eugen *7*, 179–188. https://doi.org/10.1111/j.1469-1809.1936.tb02137.x.

Gwinn, K., Devine, M.J., Jin, L.-W., Johnson, J., Bird, T., Muenter, M., Waters, C., Adler, C.H., Caselli, R., Houlden, H., et al. (2011). Clinical features, with video documentation, of the original familial lewy body parkinsonism caused by α-synuclein triplication (Iowa kindred). Mov. Disord. *26*, 2134–2136. https://doi.org/10.1002/mds.23776.

Hampson, P., Chahal, H., Khanim, F., Hayden, R., Mulder, A., Assi, L.K., Bunce, C.M., and Lord, J.M. (2005). PEP005, a selective small-molecule activator of protein kinase C, has potent antileukemic activity mediated via the delta isoform of PKC. Blood *106*, 1362–1368. https://doi.org/10.1182/blood-2004-10-4117.

Hockey, L.N., Kilpatrick, B.S., Eden, E.R., Lin-Moshier, Y., Brailoiu, G.C., Brailoiu, E., Futter, C.E., Schapira, A.H., Marchant, J.S., and Patel, S. (2015). Dysregulation of lysosomal morphology by pathogenic LRRK2 is corrected by TPC2 inhibition. J. Cell Sci. *128*, 232–238. https://doi.org/10.1242/jcs.164152.

Hsieh, C.-H., Shaltouki, A., Gonzalez, A.E., Bettencourt da Cruz, A., Burbulla, L.F., S Lawrence, E., Schüle, B., Krainc, D., Palmer, T.D., and Wang, X. (2016). Functional impairment in Miro degradation and mitophagy is a shared feature in familial and sporadic Parkinson's disease. Cell Stem Cell *19*, 709–724. https://doi.org/10.1016/j.stem.2016.08.002.

Hunter, J.D. (2007). Matplotlib: a 2D graphics environment. Comput. Sci. Eng. *9*, 90–95. https://doi.org/10.1109/MCSE.2007.55.

Iannetti, E.F., Smeitink, J.A.M., Beyrath, J., Willems, P.H.G.M., and Koopman, W.J.H. (2016). Multiplexed high-content analysis of mitochondrial morphofunction using live-cell microscopy. Nat. Protoc. *11*, 1693–1710. https://doi.org/10.1038/nprot.2016.094.

Ke, G., Meng, Q., Finley, T., Wang, T., Chen, W., Ma, W., Ye, Q., and Liu, T.-Y. (2017). LightGBM: a highly efficient gradient boosting decision tree. Adv. Neural Inf. Process. Syst. *30*.

Kriks, S., Shim, J.-W., Piao, J., Ganat, Y.M., Wakeman, D.R., Xie, Z., Carrillo-Reid, L., Auyeung, G., Antonacci, C., Buch, A., et al. (2011). Dopamine neurons derived from human ES cells efficiently engraft in animal models of Parkinson's disease. Nature *480*, 547–551. https://doi.org/10.1038/nature10648.

Laperle, A.H., Sances, S., Yucer, N., Dardov, V.J., Garcia, V.J., Ho, R., Fulton, A.N., Jones, M.R., Roxas, K.M., Avalos, P., et al. (2020). iPSC modeling of young-onset Parkinson's disease reveals a molecular signature of disease and novel therapeutic candidates. Nat. Med. *26*, 289–299. https://doi.org/10.1038/s41591-019-0739-1.

Longo, F., Mercatelli, D., Novello, S., Arcuri, L., Brugnoli, A., Vincenzi, F., Russo, I., Berti, G., Mabrouk, O.S., Kennedy, R.T., et al. (2017). Age-dependent dopamine transporter dysfunction and Serine129 phospho-α-synuclein overload in G2019S LRRK2 mice. Acta Neuropathol. Commun. *5*, 22. https://doi.org/10.1186/s40478-017-0426-8.

McKinney, W. (2010). Data structures for statistical computing in Python. Proc. 9th Python Sci. Conf., 56–61. https://doi.org/10.25080/Majora-92bf1922-00a.

Mischak, H., Pierce, J.H., Goodnight, J., Kazanietz, M.G., Blumberg, P.M., and Mushinski, J.F. (1993). Phorbol ester-induced myeloid differentiation is mediated by protein kinase C-alpha and -delta and not by protein kinase C-beta II, -epsilon, -zeta, and -eta. J. Biol. Chem. *268*, 20110–20115. https://doi.org/10.1016/S0021-9258(20)80701-7.

Obergasteiger, J., Frapporti, G., Lamonaca, G., Pizzi, S., Picard, A., Lavdas, A.A., Pischedda, F., Piccoli, G., Hilfiker, S., Lobbestael, E., et al. (2020). Kinase inhibition of G2019S-LRRK2 enhances autolysosome formation and function to reduce endogenous alpha-synuclein intracellular inclusions. Cell Death Discov. *6*, 1–13. https://doi.org/10.1038/s41420-020-0279-y.

Pedregosa, F., Varoquaux, G., Gramfort, A., Michel, V., Thirion, B., Grisel, O., Blondel, M., Prettenhofer, P., Weiss, R., Dubourg, V., et al. (2011). Scikit-learn: machine learning in Python. J. Mach. Learn. Res. *12*, 2825–2830.

Poewe, W., Seppi, K., Tanner, C.M., Halliday, G.M., Brundin, P., Volkmann, J., Schrag, A.-E., and Lang, A.E. (2017). Parkinson disease. Nat. Rev. Dis. Primer *3*, 1–21. https://doi.org/10.1038/nrdp.2017.13.

Rocha, E.M., Keeney, M.T., Maio, R.D., Miranda, B.R.D., and Greenamyre, J.T. (2022). LRRK2 and idiopathic Parkinson's disease. Trends Neurosci. *45*, 224–236. https://doi.org/10.1016/j.tins.2021.12.002.

Ryan, S.D., Dolatabadi, N., Chan, S.F., Zhang, X., Akhtar, M.W., Parker, J., Soldner, F., Sunico, C.R., Nagar, S., Talantova, M., et al. (2013). Isogenic human iPSC Parkinson's model shows nitrosative stress-induced dysfunction in MEF2-PGC1α transcription. Cell *155*, 1351–1364. https://doi.org/10.1016/j.cell.2013.11.009.

Schiff, L., Migliori, B., Chen, Y., Carter, D., Bonilla, C., Hall, J., Fan, M., Tam, E., Ahadi, S., Fischbacher, B., et al. (2022). Integrating deep learning and unbiased automated high-content screening to identify complex disease signatures in human fibroblasts. Nat. Commun. *13*, 1590. https://doi.org/10.1038/s41467-022-28423-4.

Schwab, A.J., Sison, S.L., Meade, M.R., Broniowska, K.A., Corbett, J.A., and Ebert, A.D. (2017). Decreased sirtuin deacetylase activity in LRRK2 G2019S iPSC-derived dopaminergic neurons. Stem Cell Rep. *9*, 1839–1852. https://doi.org/10.1016/j.stemcr.2017.10.010.

Sheng, Z., Zhang, S., Bustos, D., Kleinheinz, T., Le Pichon, C.E., Dominguez, S.L., Solanoy, H.O., Drummond, J., Zhang, X., Ding, X., et al. (2012). Ser1292 autophosphorylation is an indicator of LRRK2 kinase activity and contributes to the cellular effects of PD mutations. Sci. Transl. Med. *4*, 164ra161. https://doi.org/10.1126/scitranslmed.3004485.

Smith, W.W., Pei, Z., Jiang, H., Dawson, V.L., Dawson, T.M., and Ross, C.A. (2006). Kinase activity of mutant LRRK2 mediates neuronal toxicity. Nat. Neurosci. *9*, 1231–1233. https://doi.org/10.1038/nn1776.

Steger, M., Tonelli, F., Ito, G., Davies, P., Trost, M., Vetter, M., Wachter, S., Lorentzen, E., Duddy, G., Wilson, S., et al. (2016). Phosphoproteomics reveals that Parkinson's disease kinase LRRK2 regulates a subset of Rab GTPases. Elife *5*, e12813. https://doi.org/10.7554/eLife.12813.

Tabata, Y., Imaizumi, Y., Sugawara, M., Andoh-Noda, T., Banno, S., Chai, M., Sone, T., Yamazaki, K., Ito, M., Tsukahara, K., et al. (2018). T-Type calcium channels determine the vulnerability of dopaminergic neurons to mitochondrial stress in familial Parkinson disease. Stem Cell Rep. *11*, 1171–1184. https://doi.org/10.1016/j.stemcr.2018.09.006.

Volpicelli-Daley, L.A., Abdelmotilib, H., Liu, Z., Stoyka, L., Daher, J.P.L., Milnerwood, A.J., Unni, V.K., Hirst, W.D., Yue, Z., Zhao, H.T., et al. (2016). G2019S-LRRK2 expression augments α-synuclein sequestration into inclusions in neurons. J. Neurosci. *36*, 7415–7427. https://doi.org/10.1523/JNEUROSCI.3642-15.2016.

Walsh, I., Fishman, D., Garcia-Gasulla, D., Titma, T., Pollastri, G., Harrow, J., Psomopoulos, F.E., and Tosatto, S.C.E. (2021). DOME: recommendations for supervised machine learning validation in biology. Nat. Methods *18*, 1122–1127. https://doi.org/10.1038/s41592-021-01205-4.

Wang, Y., Huang, H., Rudin, C., and Shaposhnik, Y. (2021). Understanding how dimension reduction tools work: an empirical approach to deciphering t-SNE, UMAP, TriMap, and PaCMAP for data visualization. J. Mach. Learn. Res. *22*, 1–73.

Waskom, M.L. (2021). seaborn: statistical data visualization. J. Open Source Softw. *6*, 3021. https://doi.org/10.21105/joss.03021.

Weber, M. (2022). Statannot - Statistical Annotations for Boxplots/barplots Generated by Seaborn.

West, A.B., Moore, D.J., Biskup, S., Bugayenko, A., Smith, W.W., Ross, C.A., Dawson, V.L., and Dawson, T.M. (2005). Parkinson's disease-associated mutations in leucine-rich repeat kinase 2 augment kinase activity. Proc. Natl. Acad. Sci. USA *102*, 16842–16847. https://doi.org/10.1073/pnas.0507360102.

Weykopf, B., Haupt, S., Jungverdorben, J., Flitsch, L.J., Hebisch, M., Liu, G.-H., Suzuki, K., Belmonte, J.C.I., Peitz, M., Blaess, S., et al. (2019). Induced pluripotent stem cell-based modeling of mutant LRRK2-associated Parkinson's disease. Eur. J. Neurosci. *49*, 561–589. https://doi.org/10.1111/ejn.14345.

Xiong, Y., Neifert, S., Karuppagounder, S.S., Stankowski, J.N., Lee, B.D., Grima, J.C., Chen, G., Ko, H.S., Lee, Y., Swing, D., et al. (2017). Overexpression of Parkinson's disease-associated mutation LRRK2 G2019S in mouse Forebrain induces behavioral deficits and α-synuclein pathology. ENeuro *4*. ENEURO.0004-17. https://doi.org/10.1523/ENEURO.0004-17.

Yamaguchi, A., Ishikawa, K.-I., Inoshita, T., Shiba-Fukushima, K., Saiki, S., Hatano, T., Mori, A., Oji, Y., Okuzumi, A., Li, Y., et al. (2020). Identifying therapeutic agents for amelioration of mitochondrial clearance disorder in neurons of familial Parkinson disease. Stem Cell Rep. *14*, 1060–1075. https://doi.org/10.1016/j.stemcr.2020.04.011.

Zhang, J.-H., Chung, T.D.Y., and Oldenburg, K.R. (1999). A simple statistical parameter for use in evaluation and validation of high throughput screening assays. J. Biomol. Screen *4*, 67–73. https://doi.org/10.1177/108705719900400206.

Stem Cell Reports, Volume *17*

Supplemental Information

High-content phenotyping of Parkinson's disease patient stem cell-derived midbrain dopaminergic neurons using machine learning classification

Aurore Vuidel, Loïc Cousin, Beatrice Weykopf, Simone Haupt, Zahra Hanifehlou, Nicolas Wiest-Daesslé, Michaela Segschneider, Joohyun Lee, Yong-Jun Kwon, Michael Peitz, Arnaud Ogier, Laurent Brino, Oliver Brüstle, Peter Sommer, and Johannes H. Wilbertz

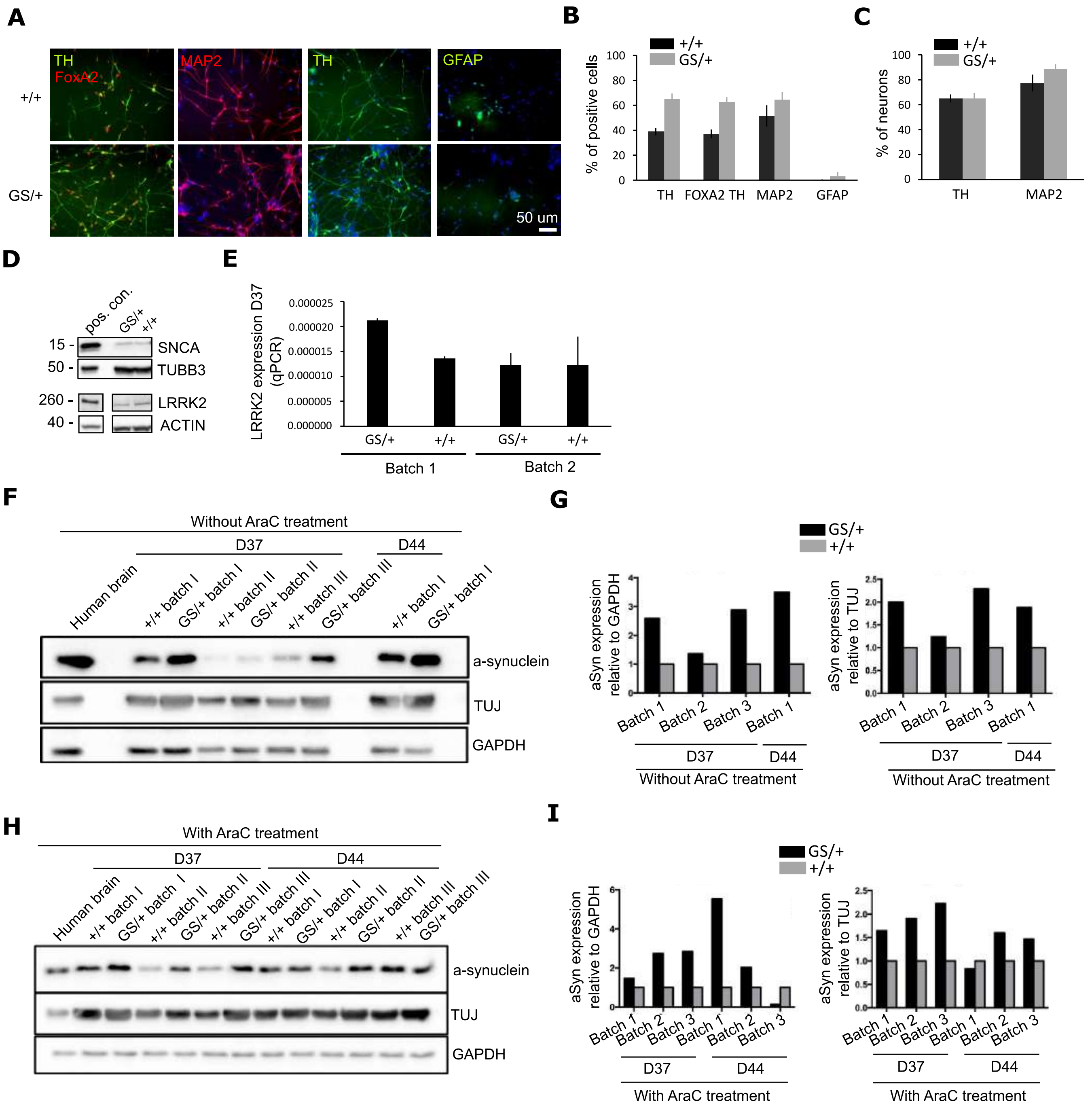

Figure S1 (referring to Figure 1): Characterization of human iPSC-derived LRRK2 G2019S mDA neurons. (A) Batches of +/+ isogenic control and GS/+ iPSCs were differentiated into mDA neurons for 30 days and cryo-preserved at 2x106 cells per well. Thawed neurons were cultured for additional 7 days and stained for Tyrosine hydroxylase (TH), Forkhead box protein A2 (FOXA2), Microtubule-associated protein 2 (MAP2), and Glial Fibrillary Acidic Protein (GFAP). **(B)** The purity of the mDA neuron population after differentiation and thawing was measured by the ratio of neuronal marker MAP2, dopaminergic marker TH, midbrain dopaminergic markers TH/FOXA2, and astrocyte marker GFAP positive cells. **(C)** The ratio of TH and MAP2 positive cells was calculated for the β-tubulin III (TUBB3) staining-positive neuronal population. **(D)** The presence of the proteins α-synuclein, TUBB3, and LRRK2 in the neuronal culture was confirmed by Western blotting. **(E)** qPCR analysis showed the expression of LRRK2 in isogenic control +/+ neurons as well as in mutation carrying GS/+ neurons. **(F)** D30 mDA neurons were cryopreserved, thawed and cultured for either 7 days (D37) or 14 days (D44) and lysed. α-synuclein, β-tubulin III (TUJ antibody) and GAPDH were detected by Western blotting. **(G)** Western blots were analyzed densiometrically and the α-synuclein level was expressed relative to GAPDH (left panel) or TUJ signal (right panel). **(H)** Experiment performed as in **(F)**, but cultured neurons were treated with the cytostatic drug cytosine arabinoside (AraC) in order to prevent glial proliferation. **(I)** Analysis was performed as in **(G)**.

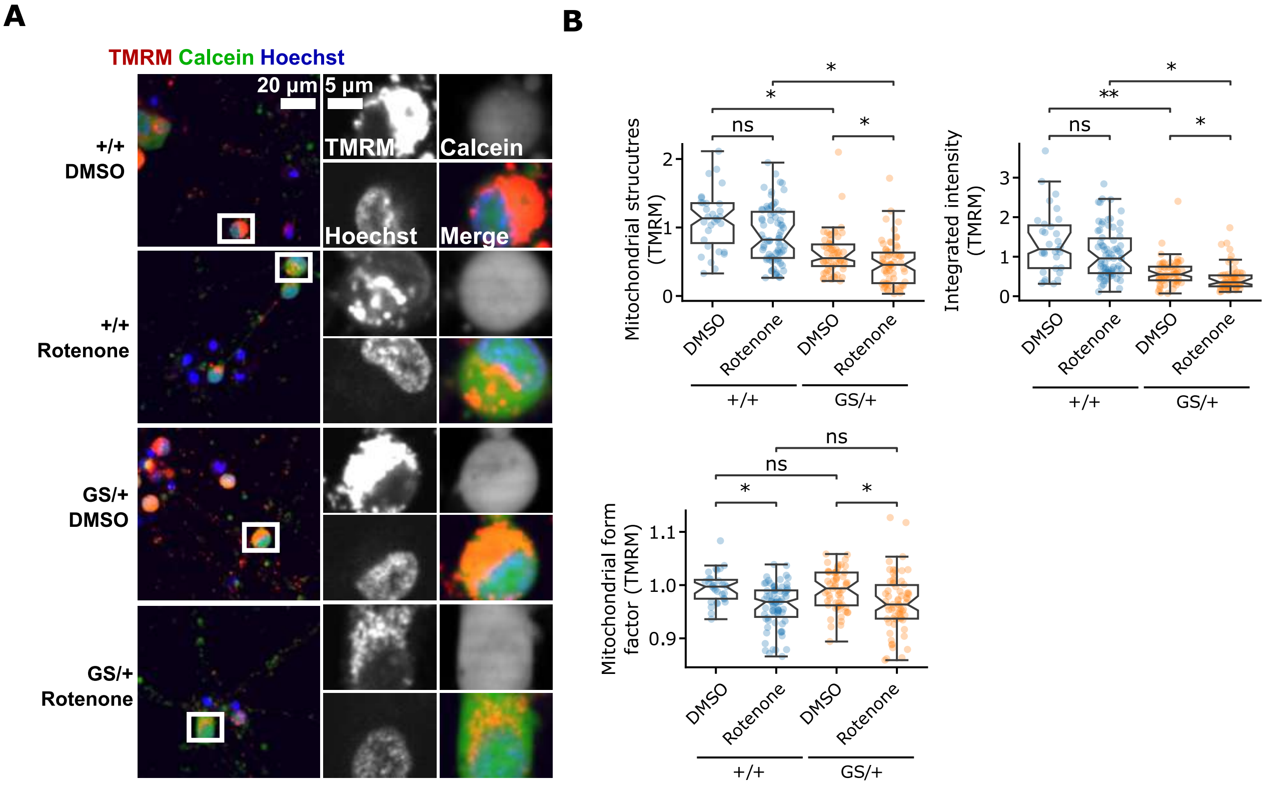

Figure S2 (referring to Figure 1): LRRK2 G2019S mDA neurons are more sensitive to the mitochondrial stressor Rotenone. (**A**) Representative images of cryopreserved D30 mDA neurons cultured for 7 days and treated with DMSO or Rotenone during the last 24 hours. Cells were stained with Hoechst, Calcein and TMRM and imaged. (**B**) Multiple mitochondrial features were quantified based on the TMRM stain including mitochondrial number, intensity, and shape.

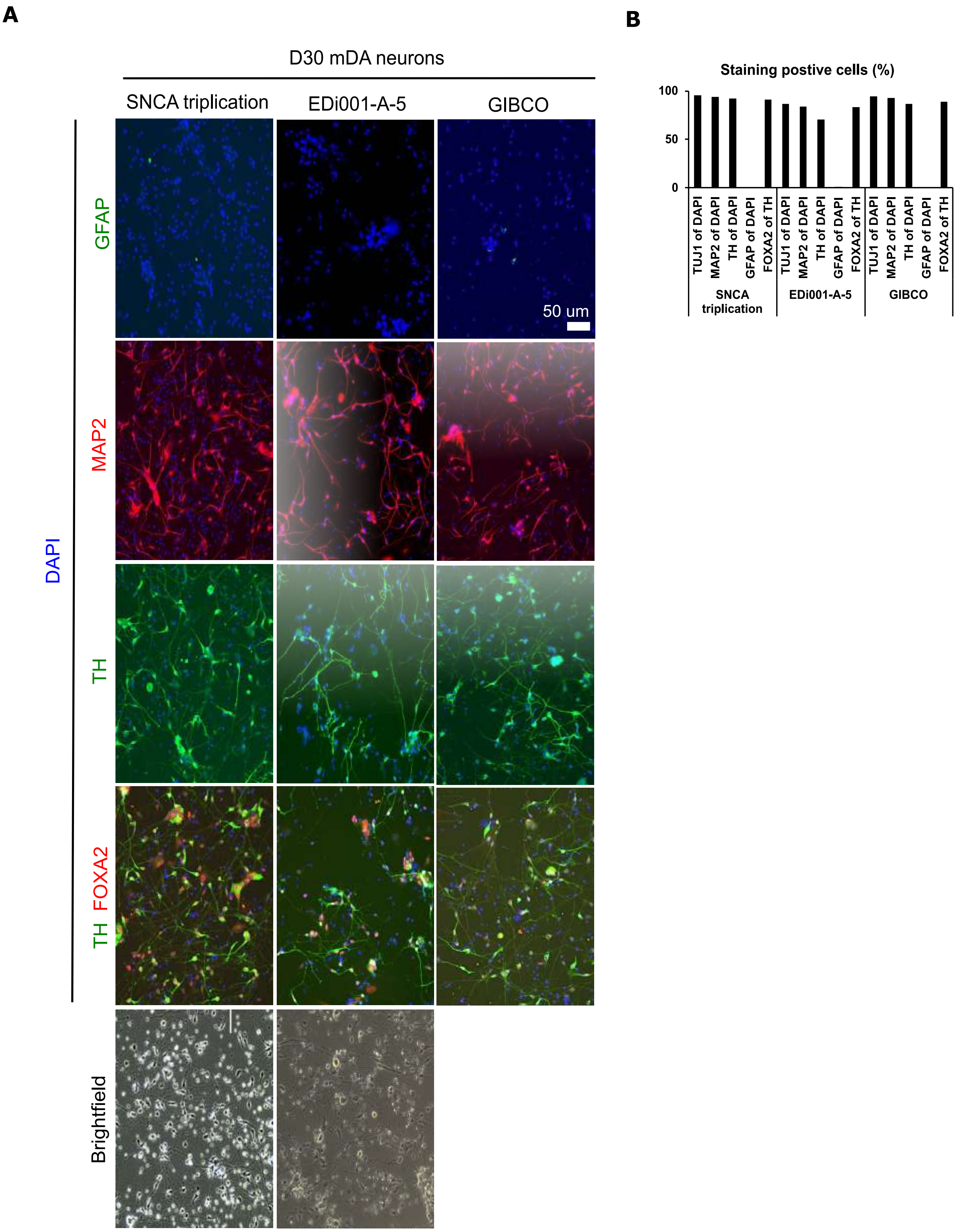

Figure S3 (referring to Figure 5): Characterization of additional (non-LRRK2) human iPSC-derived mDA neurons. (**A**) Batches of SNCA triplication, isogenic control (Edi001-A-5) and a genetically unrelated control (Gibco) iPSCs were differentiated into mDA neurons for 30 days and cryo-preserved at 2x106 cells per well. Thawed neurons were cultured for additional 7 days and stained for Tyrosine hydroxylase (TH), Forkhead box protein A2 (FOXA2), Microtubule-associated protein 2 (MAP2), and Glial Fibrillary Acidic Protein (GFAP). Representative images are shown. (**B**) Quantification of staining positive cells.

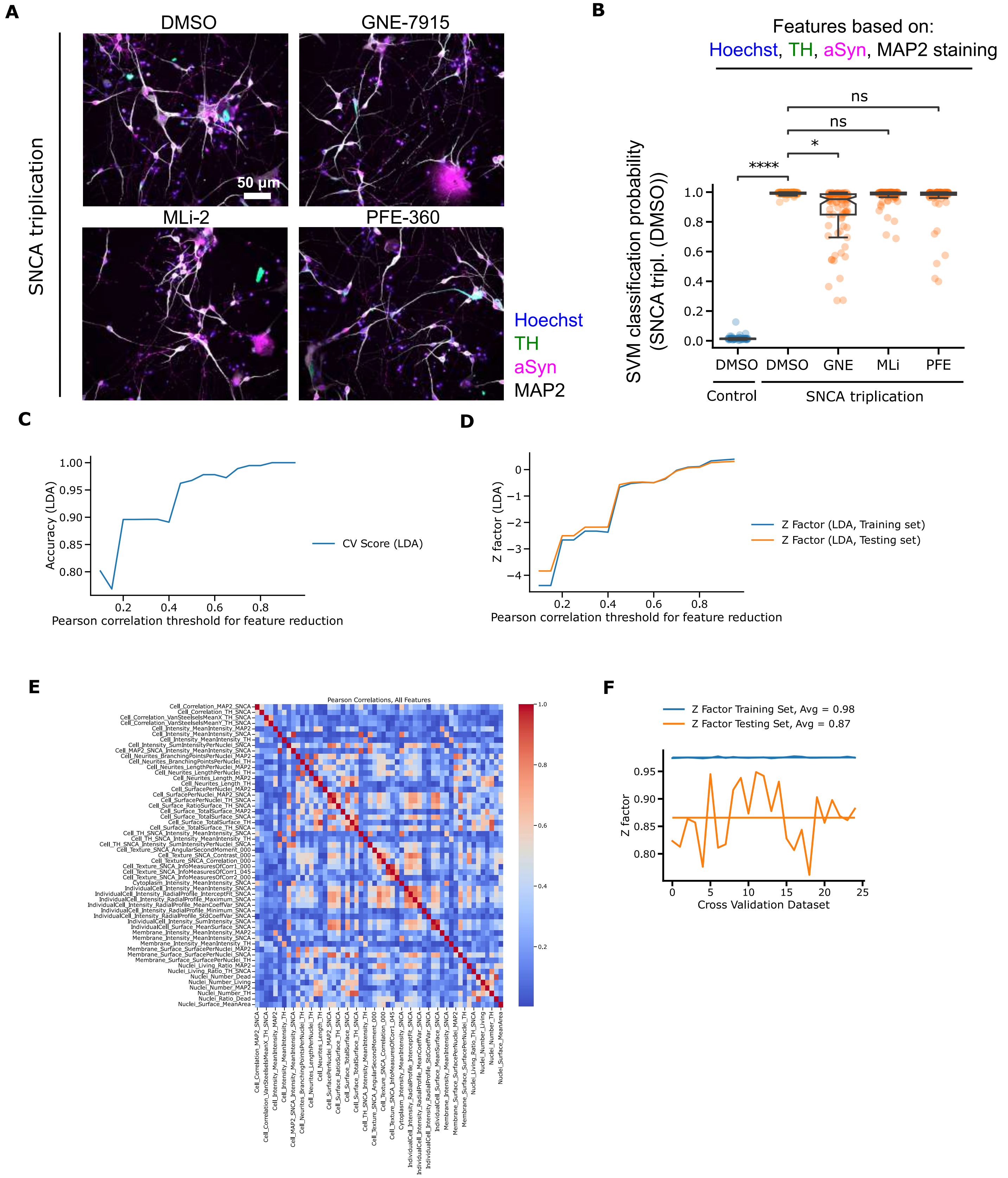

Figure S4 (referring to Figure 3): Feature selection, training and testing of Support Vector Machine (SVM) classifier using Hoechst/TH/α-synuclein/MAP2 staining derived features originating from SNCA triplication and isogenic control neurons. (**A**) Representative images of mDA neurons treated with vehicle control DMSO or LRRK2 inhibitors GNE-7915, MLi-2, and PFE-360. (**B**) SVM classification of SNCA triplication and isogenic control mDANs and mapping of neurons treated with the LRRK2 inhibitors GNE-7915, MLi-2 and PFE-360 to the reference classes' feature space. (**C**) Linear Discriminant Analysis (LDA) classification was used to select image features based on Hoechst/TH/α-synuclein/MAP2 staining that are not strongly correlated. LDA classifier accuracy is shown as a function of Pearson's correlation thresholds. (D) LDA training and testing set Z-factors between isogenic control and SNCA triplication reference classes as a function of Pearson's correlation thresholds used to exclude correlated image features. (E) Pearson's correlation matrix of all selected image features for model training with threshold 0.95. (**F**) Performance of SVM classifier during 25 cycles of training (80% of data) and testing (20% of data) using 25 shuffled data sets. Z-factor values were calculated between isogenic control and SNCA triplication reference classes.

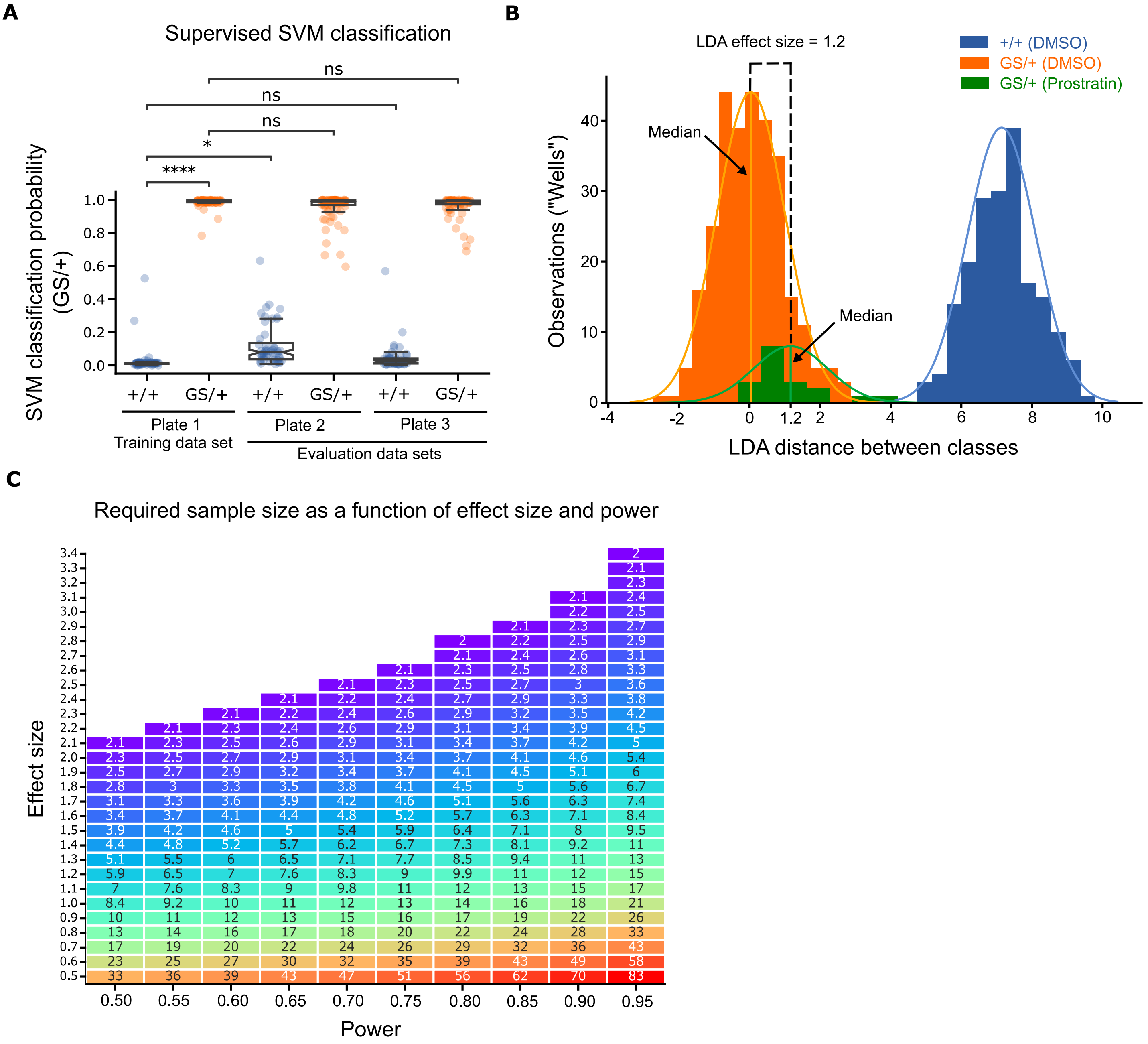

Figure S5 (referring to Figures 3-5): Parameter determination for neuronal image-based phenotyping. (**A**) A Support Vector Machine (SVM) model was trained to separate the two reference classes GS/+ and +/+ isogenic control mDANs originating from a single 384-well plate (Plate 1). Image features based on Hoechst, TH, α-synuclein and MAP2 staining were used. To evaluate the trained model, GS/+ and +/+ neuronal image features originating from two additional plates (Plate 2 and 3) where then mapped to the reference classes' feature space originating from Plate 1. (**B**) Distributions of LDA distances between experimental classes. The same data as in Figure 4 was used and Gaussian distributions were fitted. The broken lines indicate the effect size of 1.2 between the two treatment conditions GS/+ (DMSO) and GS/+ (Prostratin). (**C**) Required sample size (number of wells) as a function of the expected effect size and desired power to obtain at least one hit larger than the GS/+ (DMSO) median + 3x SD. The experimentally determined GS/+ (DMSO) distribution in (B) was used to calculate the median + 3x SDs window. Power can be defined as the probability that a test of significance will pick up on an effect that is present. Values >0.8 are typically desired.

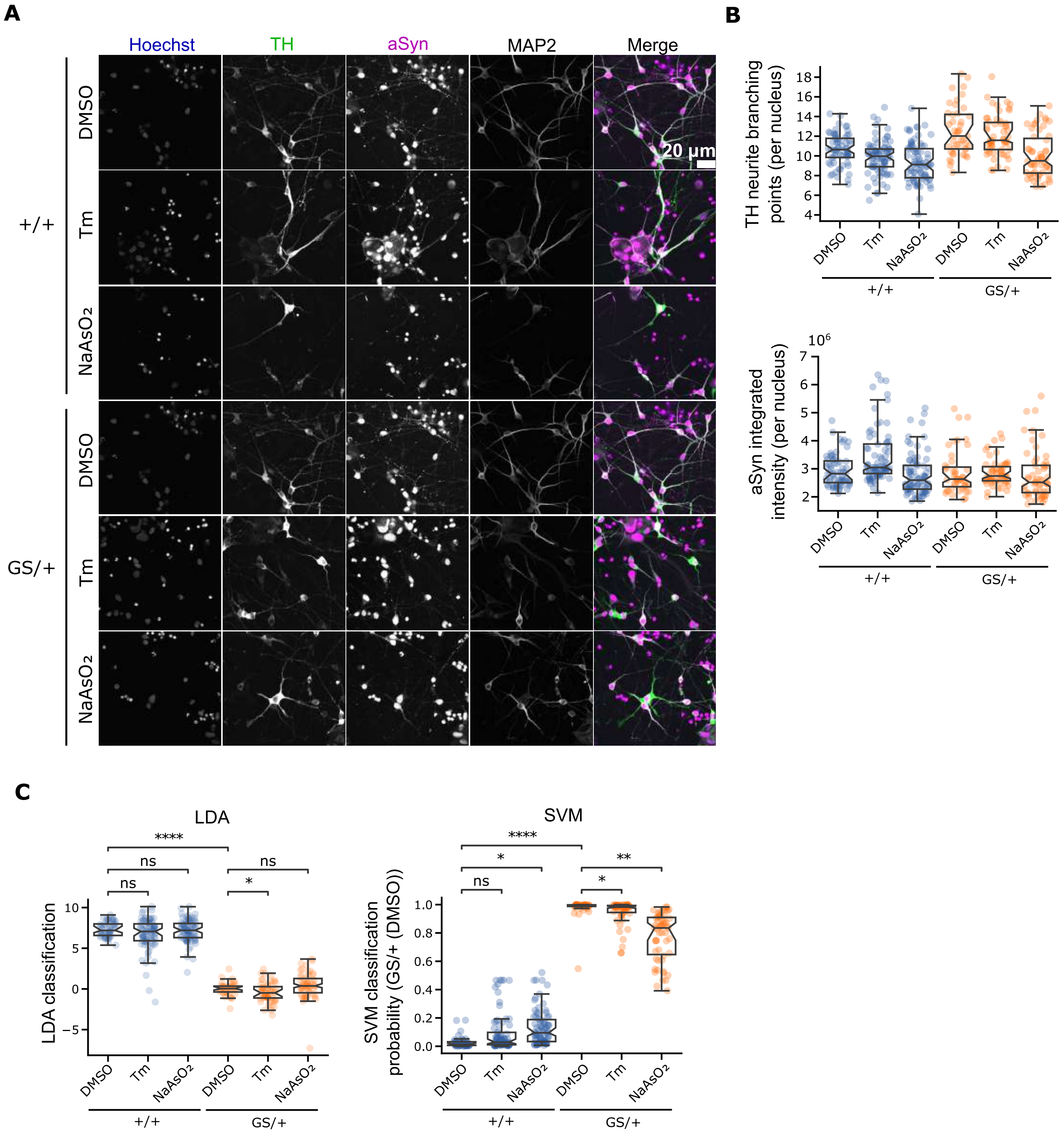

Figure S6 (referring to Figure 3): Multi-image feature guided detection of chemically stressed mDA neurons. (**A**) Representative images of mDA neurons treated for 3 hours with DMSO, 2µM Tunicamycin (Tm) or 50µM Sodium Arsenite ($NaAsO_2$). (**B**) Based on raw images, tyrosine hydroxylase (TH) neurite branching points and α-synuclein staining intensity was qunatified. **(C)** Linear Discriminant Analysis (LDA) and Support Vector Machine (SVM) classification of GS/+ and +/+ control mDANs based on cellular image features extracted from Hoechst, α-synuclein, TH, and MAP2 staining. Imaging data was generated in duplicate experiments with multiple technical replicates per plate. Each data point represents one well. Mann-Whitney U-testing was performed for significance testing. Notches in boxplots indicate the 95% confidence interval.

Table S1: Description of extracted image features after Hoechst/TH/α-synuclein/MAP2 staining and fluorescence channel segmentation.

	Feature name	Feature description
1	Cell_Correlation_MAP2_SNCA	Pearson correlation between MAP2 and SNCA channel
2	Cell_Correlation_TH_SNCA	Pearson correlation between TH and SNCA channel
3	Cell_Correlation_VanSteelselsMeanX_TH_SNCA	Van Steelsel's cross correlation between TH and SNCA channel, shift on x-axis
4	Cell_Correlation_VanSteelselsMeanY_TH_SNCA	Van Steelsel's cross correlation between TH and SNCA channel, shift on y-axis
5	Cell_Correlation_VanSteelselsSigmaX_TH_SNCA	SD of Van Steelsel's cross correlation between TH and SNCA channel, shift on x-axis
6	Cell_Correlation_VanSteelselsSigmaY_TH_SNCA	SD of Van Steelsel's cross correlation between TH and SNCA channel, shift on y-axis
7	Cell_Intensity_MeanIntensity_MAP2	Mean pixel intensity of MAP2 channel
8	Cell_Intensity_MeanIntensity_SNCA	Mean pixel intensity of SNCA channel
9	Cell_Intensity_MeanIntensity_TH	Mean pixel intensity of TH channel
10	Cell_Intensity_SumIntensityPerNuclei_SNCA	Integrated pixel intensity of SNCA channel normalized to number of nuclei
11	Cell_MAP2_SNCA_Intensity_MeanIntensity_SNCA	Mean pixel intensity of SNCA channel colocalized to MAP2 channel
12	Cell_Neurites_BranchingPointsPerNuclei_MAP2	Dendritic branching points of MAP2 channel normalized to number of nuclei
13	Cell_Neurites_BranchingPointsPerNuclei_TH	Dendritic branching points of TH channel normalized to number of nuclei
14	Cell_Neurites_LengthPerNuclei_MAP2	Dentritic network length of MAP2 channel normalized to number of nuclei
15	Cell_Neurites_LengthPerNuclei_TH	Dentritic network length of TH channel normalized to number of nuclei
16	Cell_Neurites_Length_MAP2	Dentritic network length of MAP2 channel
17	Cell_Neurites_Length_TH	Dentritic network length of TH channel
18	Cell_SurfacePerNuclei_MAP2	Surface pixels occupied by MAP2 channel, normalized to number of nuclei
19	Cell_SurfacePerNuclei_MAP2_SNCA	Surface pixels occupied by colocalized MAP2 and SNCA channel normalized to number of nuclei
20	Cell_SurfacePerNuclei_SNCA	Surface pixels occupied by SNCA channel, normalized to number of nuclei
21	Cell_SurfacePerNuclei_TH	Surface pixels occupied by TH channel, normalized to number of nuclei
22	Cell_SurfacePerNuclei_TH_SNCA	Surface pixels occupied by colocalized TH and SNCA channel normalized to number of nuclei
23	Cell_Surface_RatioSurface_TH_SNCA	Surface ratio occupied by colocalized TH and SNCA channel
24	Cell_Surface_TotalSurface_MAP2	Surface pixels occupied by MAP2 channel
25	Cell_Surface_TotalSurface_SNCA	Surface pixels occupied by SNCA channel
26	Cell_Surface_TotalSurface_SNCA_MAP2	Surface pixels occupied by colocalized SNCA and MAP2 channel
27	Cell_Surface_TotalSurface_TH	Surface pixels occupied by TH channel
28	Cell_Surface_TotalSurface_TH_SNCA	Surface pixels occupied by colocalized TH and SNCA channel
29	Cell_TH_SNCA_Intensity_MeanIntensity_SNCA	Mean pixel intensity of SNCA channel colocalized to TH channel
30	Cell_TH_SNCA_Intensity_MeanIntensity_TH	Mean pixel intensity of TH channel colocalized to SNCA channel
31	Cell_TH_SNCA_Intensity_SumIntensityPerNuclei_SNCA	Integrated pixel intensity of SNCA channel colocalized to TH channel normalized to number of nuclei
32	Cell_Texture_SNCA_AngularSecondMoment_000	Haralick uniformity of distribution of gray levels at 0 degree shift
33	Cell_Texture_SNCA_AngularSecondMoment_045	Haralick uniformity of distribution of gray levels at 45 degree shift
34	Cell_Texture_SNCA_AngularSecondMoment_090	Haralick uniformity of distribution of gray levels at 90 degree shift
35	Cell_Texture_SNCA_AngularSecondMoment_135	Haralick uniformity of distribution of gray levels at 135 degree shift
36	Cell_Texture_SNCA_Contrast_000	Haralick contrast of gray levels at 0 degree shift
37	Cell_Texture_SNCA_Contrast_045	Haralick contrast of gray levels at 45 degree shift
38	Cell_Texture_SNCA_Contrast_090	Haralick contrast of gray levels at 90 degree shift
39	Cell_Texture_SNCA_Contrast_135	Haralick contrast of gray levels at 135 degree shift
40	Cell_Texture_SNCA_Correlation_000	Haralick correlation of gray levels at 0 degree shift
41	Cell_Texture_SNCA_Correlation_045	Haralick correlation of gray levels at 45 degree shift
42	Cell_Texture_SNCA_Correlation_090	Haralick correlation of gray levels at 90 degree shift
43	Cell_Texture_SNCA_Correlation_135	Haralick correlation of gray levels at 135 degree shift
44	Cell_Texture_SNCA_DifferenceEntropy_000	Haralick difference of randomness of gray levels at 0 degree shift
45	Cell_Texture_SNCA_DifferenceEntropy_045	Haralick difference of randomness of gray levels at 45 degree shift
46	Cell_Texture_SNCA_DifferenceEntropy_090	Haralick difference of randomness of gray levels at 90 degree shift
47	Cell_Texture_SNCA_DifferenceEntropy_135	Haralick difference of randomness of gray levels at 135 degree shift
48	Cell_Texture_SNCA_DifferenceVariance_000	Haralick difference of variance of gray levels at 0 degree shift
49	Cell_Texture_SNCA_DifferenceVariance_045	Haralick difference of variance randomness of gray levels at 45 degree shift
50	Cell_Texture_SNCA_DifferenceVariance_090	Haralick difference of variance randomness of gray levels at 90 degree shift
51	Cell_Texture_SNCA_DifferenceVariance_135	Haralick difference of variance randomness of gray levels at 135 degree shift
52	Cell_Texture_SNCA_Entropy_000	Haralick randomness of gray levels at 0 degree shift
53	Cell_Texture_SNCA_Entropy_045	Haralick randomness of gray levels at 45 degree shift
54	Cell_Texture_SNCA_Entropy_090	Haralick randomness of gray levels at 90 degree shift
55	Cell_Texture_SNCA_Entropy_135	Haralick randomness of gray levels at 135 degree shift
56	Cell_Texture_SNCA_InfoMeasuresOfCorr1_000	Haralick information measure of correlation 1 of gray levels at 0 degree shift
57	Cell_Texture_SNCA_InfoMeasuresOfCorr1_045	Haralick information measure of correlation 1 of gray levels at 45 degree shift
58	Cell_Texture_SNCA_InfoMeasuresOfCorr1_090	Haralick information measure of correlation 1 of gray levels at 90 degree shift
59	Cell_Texture_SNCA_InfoMeasuresOfCorr1_135	Haralick information measure of correlation 1 of gray levels at 135 degree shift
60	Cell_Texture_SNCA_InfoMeasuresOfCorr2_000	Haralick information measure of correlation 2 of gray levels at 0 degree shift
61	Cell_Texture_SNCA_InfoMeasuresOfCorr2_045	Haralick information measure of correlation 2 of gray levels at 45 degree shift
62	Cell_Texture_SNCA_InfoMeasuresOfCorr2_090	Haralick information measure of correlation 2 of gray levels at 90 degree shift
63	Cell_Texture_SNCA_InfoMeasuresOfCorr2_135	Haralick information measure of correlation 2 of gray levels at 135 degree shift
64	Cell_Texture_SNCA_InverseDiffMoment_000	Haralick homogeneity of gray levels at 0 degree shift
65	Cell_Texture_SNCA_InverseDiffMoment_045	Haralick homogeneity of gray levels at 45 degree shift
66	Cell_Texture_SNCA_InverseDiffMoment_090	Haralick homogeneity of gray levels at 90 degree shift
67	Cell_Texture_SNCA_InverseDiffMoment_135	Haralick homogeneity of gray levels at 135 degree shift
68	Cell_Texture_SNCA_SumAverage_000	Haralick sum of averages of gray levels at 0 degree shift
69	Cell_Texture_SNCA_SumAverage_045	Haralick sum of averages of gray levels at 45 degree shift
70	Cell_Texture_SNCA_SumAverage_090	Haralick sum of averages of gray levels at 90 degree shift
71	Cell_Texture_SNCA_SumAverage_135	Haralick sum of averages of gray levels at 135 degree shift
72	Cell_Texture_SNCA_SumEntropy_000	Haralick sum of gray level randomness at 0 degree shift
73	Cell_Texture_SNCA_SumEntropy_045	Haralick sum of gray level randomness at 45 degree shift
74	Cell_Texture_SNCA_SumEntropy_090	Haralick sum of gray level randomness at 90 degree shift
75	Cell_Texture_SNCA_SumEntropy_135	Haralick sum of gray level randomness at 135 degree shift
76	Cell_Texture_SNCA_SumOfSquares_000	Haralick sum of square gray level variance at 0 degree shift
77	Cell_Texture_SNCA_SumOfSquares_045	Haralick sum of square gray level variance at 45 degree shift
78	Cell_Texture_SNCA_SumOfSquares_090	Haralick sum of square gray level variance at 90 degree shift
79	Cell_Texture_SNCA_SumOfSquares_135	Haralick sum of square gray level variance at 135 degree shift
80	Cell_Texture_SNCA_SumVariance_000	Haralick sum of gray level variance at 0 degree shift

Table S1 (continued)

81	Cell_Texture_SNCA_SumVariance_045	Haralick sum of gray level variance at 45 degree shift
82	Cell_Texture_SNCA_SumVariance_090	Haralick sum of gray level variance at 90 degree shift
83	Cell_Texture_SNCA_SumVariance_135	Haralick sum of gray level variance at 135 degree shift
84	Cytoplasm_Intensity_MeanIntensity_SNCA	Mean pixel intensity of cytoplasmic SNCA channel
85	Cytoplasm_MAP2_SNCA_Intensity_MeanIntensity_SNC	Mean pixel intensity of cytoplasmic SNCA channel colocalized to MAP2 channel
86	Cytoplasm_SurfacePerNuclei_SNCA	Surface pixels occupied by cytoplasmic SNCA channel normalized to number of nuclei
87	Cytoplasm_SurfacePerNuclei_TH_SNCA	Surface pixels occupied by colocalized cytoplasmic TH and SNCA channel normalized to number of nuclei
88	Cytoplasm_Surface_TotalSurface_SNCA	Surface pixels occupied by cytoplasmic SNCA channel
89	IndividualCell_Intensity_MeanIntensity_SNCA	Mean pixel intensity of SNCA channel based on all individually segmented cells
90	IndividualCell_Intensity_RadialProfile_InterceptFit_SNC	Fitted intercept of SNCA channel decay from center to edge
91	IndividualCell_Intensity_RadialProfile_MaxSlope_SNCA	Maximum steepness of SNCA channel decay from center to edge
92	IndividualCell_Intensity_RadialProfile_Maximum_SNCA	Maximum intensity of SNCA channel from center to edge
93	IndividualCell_Intensity_RadialProfile_MeanCoeffVar_SN	Mean SNCA channel dispersion from center to edge
94	IndividualCell_Intensity_RadialProfile_MeanGradient_SN	Mean shape of SNCA channel decay from center to edge
95	IndividualCell_Intensity_RadialProfile_Mean_SNCA	Mean intensity of SNCA channel from center to edge
96	IndividualCell_Intensity_RadialProfile_Median_SNCA	Median intensity of SNCA channel from center to edge
97	IndividualCell_Intensity_RadialProfile_Minimum_SNCA	Minimum intensity of SNCA channel from center to edge
98	IndividualCell_Intensity_RadialProfile_Q1_SNCA	First quartile intensity of SNCA channel from center to edge
99	IndividualCell_Intensity_RadialProfile_Q3_SNCA	Third quartile intensity of SNCA channel from center to edge
100	IndividualCell_Intensity_RadialProfile_SlopeFit_SNCA	Fitted slope of SNCA channel decay from center to edge
101	IndividualCell_Intensity_RadialProfile_StdCoeffVar_SNC	SD of SNCA channel dispersion from center to edge
102	IndividualCell_Intensity_RadialProfile_Std_SNCA	SD of SNCA channel intensity from center to edge
103	IndividualCell_Intensity_SumIntensity_SNCA	Integrated pixel intensity of SNCA channel based on all individually segmented cells
104	IndividualCell_Surface_MeanSurface_SNCA	Mean surface pixels occupied by SNCA channel based on all individually segmented cells
105	Membrane_Intensity_MeanIntensity_MAP2	Mean pixel intensity of MAP2 channel on cellular edge
106	Membrane_Intensity_MeanIntensity_SNCA	Mean pixel intensity of TH channel on cellular edge
107	Membrane_Intensity_MeanIntensity_TH	Mean pixel intensity of MAP2 channel on cellular edge
108	Membrane_Surface_SurfacePerNuclei_MAP2	Surface pixels on cellular edge occupied by MAP2 channel normalized to number of nuclei
109	Membrane_Surface_SurfacePerNuclei_SNCA	Surface pixels on cellular edge occupied by SNCA channel normalized to number of nuclei
110	Membrane_Surface_SurfacePerNuclei_TH	Surface pixels on cellular edge occupied by TH channel normalized to number of nuclei
111	Nuclei_Living_Ratio_MAP2	Ratio of MAP2 channel positive nuclei
112	Nuclei_Living_Ratio_MAP2_SNCA	Ratio of MAP2 and SNCA channel positive nuclei
113	Nuclei_Living_Ratio_SNCA	Ratio of SNCA channel positive nuclei
114	Nuclei_Living_Ratio_TH	Ratio of TH channel positive nuclei
115	Nuclei_Living_Ratio_TH_SNCA	Ratio of TH and SNCA channel positive nuclei
116	Nuclei_Number_Big	Number of large nuclei
117	Nuclei_Number_Dead	Number of condensed/bright nuclei
118	Nuclei_Number_Living	Number of nuclei based on Hoechst channel
119	Nuclei_Number_MAP2	Number of MAP2 channel positive nuclei
120	Nuclei_Number_MAP2_SNCA	Number of MAP2 and SNCA channel positive nuclei
121	Nuclei_Number_SNCA	Number of SNCA channel positive nuclei
122	Nuclei_Number_TH	Number of TH channel positive nuclei
123	Nuclei_Number_TH_SNCA	Number of TH and SNCA channel positive nuclei
124	Nuclei_Ratio_Dead	Ratio of condensed/bright nuclei
125	Nuclei_Ratio_Living	Ratio of nuclei not considered condensed/bright
126	Nuclei_Surface_MeanArea	Mean surface pixels of Hoechst channel

Table S2: Description of extracted image features after Hoechst/Calcein/TMRM staining and fluorescence channel segmentation.

	Feature name	Feature description
1	avg_compactness_all	Average Compactness = area * 4 * pi / major axis : all the components
2	avg_compactness_calcein	Average Compactness = area * 4 * pi / major axis : only the components in the calcein mask
3	avg_compactness_not_calcein	Average Compactness = area * 4 * pi / major axis : only the components not in the calcein mask
4	avg_eccentricities_all	Average Eccentricity = sqrt(1 - (minor axis/major axis)^2) : all the components
5	avg_eccentricities_not_calcein	Average Eccentricity = sqrt(1 - (minor axis/major axis)^2) : only the components not in the calcein mask
6	avg_form_factor_all	Average Form Factor = 4*pi*area/perimeter : all the components
7	avg_form_factor_calcein	Average Form Factor = 4*pi*area/perimeter : only the components in the calcein mask
8	avg_form_factor_not_calcein	Average Form Factor = 4*pi*area/perimeter : only the components not in the calcein mask
9	avg_intensity_calcein	Average intensity of the calcein channel
10	avg_intensity_tmrm	Average intensity of the TMRM channel
11	avg_major_axis_all	Average major axis : all the components
12	avg_major_axis_calcein	Average major axis : only the components in the calcein mask
13	avg_major_axis_not_calcein	Average major axis : only the components not in the calcein mask
14	avg_minor_axis_all	Average minor axis : all the components
15	avg_minor_axis_calcein	Average minor axis : only the components in the calcein mask
16	avg_minor_axis_not_calcein	Average minor axis : only the components not in the calcein mask
17	avg_nb_tmrm_calcein_per_nuclei	Average number of components tmrm in calcein mask normalized per nulcei TMRM positive
18	avg_nb_tmrm_per_nuclei	Total number of components tmrm normalized per nulcei TMRM positive
19	avg_perimeters_all	Average perimeter of components : all the components
20	avg_perimeters_calcein	Average perimeter of components : only the components in the calcein mask
21	avg_perimeters_not_calcein	Average perimeter of components : only the components not in the calcein mask
22	compo_avg_intensity_tmrm_calcein	Average TMRM intensity per component : only the components in the calcein mask
23	compo_avg_intensity_tmrm_not_calcein	Average TMRM intensity per component : only the components not in the calcein mask
24	compo_avg_sum_intensity_tmrm_calcein	Average sum of TMRM intensity per component : only the components in the calcein mask
25	compo_avg_sum_intensity_tmrm_not_calcein	Average sum of TMRM intensity per component : only the components not in the calcein mask
26	compo_avg_surface_tmrm_calcein	Average surface of TMRM components : only the components in the calcein mask
27	compo_avg_surface_tmrm_not_calcein	Average surface of TMRM components : only the components not in the calcein mask
28	dead_nuclei	Number of dead cells
29	living_nuclei	Number of living cells
30	nuclei_tot	Total number of cells
31	ratio_dead_nuclei	Ratio of dead cells
32	ratio_living_nuclei	Ratio of living cells
33	ratio_nb_tmrm_calcein	Ratio of TMRM components in the calcein mask
34	ratio_nb_tmrm_not_calcein	Ratio of TMRM components in the not calcein mask
35	sum_intensity_calcein_per_nuclei	Sum of intensities of calcein channel normalized by living cells
36	sum_intensity_calcein_tot	Sum of intensities of calcein channel
37	sum_intensity_calcein_tot_per_nuclei	Sum of intensities of calcein channel normalized by living cells
38	sum_intensity_tmrm_calcein	Sum of intensities of TMRM channel in the calcein mask
39	sum_intensity_tmrm_calcein_per_nuclei	Sum of intensities of TMRM channel in the calcein mask normalized by calcein cell positive cell number
40	sum_intensity_tmrm_not_calcein	Sum of intensities of TMRM channel not in the calcein mask
41	sum_intensity_tmrm_not_calcein_per_nuclei	Sum of intensities of TMRM channel not in the calcein mask normalized by calcein cell positive cell number
42	sum_intensity_tmrm_tot	Sum of intensities of TMRM channel
43	sum_intensity_tmrm_tot_per_nuclei	Sum of intensities of TMRM channel normalized by living cell number
44	surface_calcein	Surface of calcein channel above the threshold
45	surface_calcein_per_nuclei_calcein	Surface of calcein channel above the threshold normalized by calcein positive cell number
46	surface_tmrm_calcein	Total surface of TMRM in calcein mask.
47	surface_tmrm_per_nuclei_not_calcein	Surface of TMRM not in the calcein mask normalized by negative calcein cell number
48	surface_tmrm_per_nuclie_calcein	Surface of TMRM in the calcein mask normalized by positive calcein cell number
49	total_nb_tmrm	Total number of components TMRM
50	total_nb_tmrm_calcein	Total number of components TMRM in calcein mask
51	avg_eccentricities_calcein	Average Eccentricity = sqrt(1 - (minor axis/major axis)^2) : only the components in the calcein mask
52	avg_nb_tmrm_not_calcein_per_nuclei	Number of TMRM components not in calcein mask normalized by negative calcein cell number
53	surface_tmrm	Total surface of TMRM channel above the threshold
54	total_nb_tmrm_not_calcein	Total number of TMRM components not in the calcein mask

Table S3: Used cell lines, antibodies, primers, and key reagents.

**Cell lines**					
**Genotype**	**hPSCreg name**	**Donor source**	**Provider**	**Reprogramming method**	**Ref**
LRRK2 G2019S	STBCi004-B (GS/+)	Female, dermal fibroblasts	Distributor: EBiSC; Generator: StemBANCC	Non-integrating Sendai virus	(Morrison et al., 2015)
Corrected LRRK2 G2019S mutation in STBCi004-B	STBCi004-B-1 (+/+)	Female, dermal fibroblasts	Distributor: EBiSC; Generator: StemBANCC	Non-integrating Sendai virus	(Morrison et al., 2015)
SNCA triplication	EDi001-A (AST23)	Female, dermal fibroblasts	Distributor: EBiSC; Generator: University of Edinburgh	Integrating Retro virus	(Devine et al., 2011; Gwinn et al., 2011)
Corrected SNCA triplication in EDi001-A	EDi001-A-5 (AST23-2KO-8B)	Female, dermal fibroblasts	Distributor: EBiSC; Generator: University of Edinburgh	Integrating Retro virus	(Devine et al., 2011; Gwinn et al., 2011)
No known mutations	TMOi001-A (Gibco A18944)	Female, CD34+ cord blood	Distributor: EBiSC; Generator: ThermoFisher	Non-integrating Epstein-Barr virus	(Burridge et al., 2011)

**Antibodies**			
**Antibody**	**Assay**	**Dilution**	**Distributor**
TH	ICC (quality control)	1/1500, 5% BSA + PBS	Millipore, #AB152
FOXA2	ICC (quality control)	1/200 5% BSA + PBS	Biotechne, #AF2400
MAP2	ICC (quality control)	1/1000, 5% BSA + PBS	Sigma, #M4403
GFAP	ICC (quality control)	1/1500, 5% BSA + PBS	Merck, #AB5804
α-synuclein	WB	1/1000 in Invitrogen™ iBind™ Flex Solution Kit	Novus, #NBP1-05194
TUBB3/TUJ	WB	1/1000, 5% milk + TBS-T, 2h, RT	Cell Signaling, #5568
LRRK2	WB	1/500, 5% milk + TBS-T, 2h, RT	NeuroMab, #N241A/34
Actin	WB	1/1000, 5% milk + TBS-T, 2h, RT	Cell Signaling, #58169
GAPDH	WB	1/1000 in Invitrogen™ iBind™ Flex Solution Kit	Cell Signaling, #2118
Anti-Rabbit IgG HRP-linked	WB	1/1000, 5% milk + TBS-T, 1h, RT	Cell Signaling, #7074S
α-synuclein rabbit	ICC	1/500, 5% FBS + 0.1% Triton X-100 + PBS, overnight, 4°C	Abcam, #138501
TH	ICC	1/1000, 5% FBS + 0.1% Triton X-100 + PBS, overnight, 4°C	Merck, #T2928
MAP2	ICC	1/5000, 5% FBS + 0.1% Triton X-100 + PBS, overnight, 4°C	Novus, #NB300-213
pS129 α-synuclein	ICC	1/500, 5% FBS + 0.1% Triton X-100 + PBS, overnight, 4°C	Cell Signaling, #23706S
α-synuclein mouse	ICC	1/500, 5% FBS + 0.1% Triton X-100 + PBS, overnight, 4°C	BD Biosciences, #610787
Alexa Fluo 488, Anti-Mouse	ICC	1/1000, 5% FBS + 0.1% Triton X-100 + PBS, 1h, RT	ThermoFisher, #A11001
Alexa Fluor 647 Anti-Chicken	ICC	1/250, 5% FBS + 0.1% Triton X-100 + PBS, 1h, RT	Jackson Immuno Research, #703-605-155

Table S3 (continued)

Alexa Fluo 555, Anti-Rabbit	ICC	1/1000, 5% FBS + 0.1% Triton X-100 + PBS, 1h, RT	ThermoFisher, #A21429		
TMRM	ICC	25nM, Differentiation medium, 30 min, 37°C	ThermoFisher, #T668		
Calcein	ICC	1.25μM, Differentiation medium, 30 min, 37°C	ThermoFisher, #C3100MP		
Hoechst 33342	ICC	1/2000, 30 min, 37°C for live staining or 1/3000 1h, RT for ICC	Sigma, # 14533		
**Primers**					
**LRRK2 Primer**	**Melting temp (˚C)**	**Annealing temp (˚C)**	**GC content (%)**	**Sequence**	**Product size (bp)**
FWD	58.98	55.98	50	GCTTGTTGTTGGACAGCTGA	Genomic: 1262
REV	58.97	55.97	50	GCTTGTTGTTGGACAGCTGA	mRNA: 224
**Media**					
**Reagents**	**Stock**	**Dilution**	**Distributor**		
Basal medium					
Neurobasal medium		1	Gibco, #21103-049		
GlutaMAX	100x	1:100	Gibco, #25030-081		
Pen/Strep	100x	1:100	Gibco, #15070-063		
B27	50x	1:50	Gibco, #12587-010		
Differentiation medium					
Basal medium		1			
BDNF	10 μg/mL	1:500	Cell Guidance Sys., #GFH1-10		
GDNF	10 μg/mL	1:500	Cell Guidance Sys., #GFH2-10		
LAAP	221 mM	1:1000	Sigma, #A8960-5G		
DAPT	10 mM	1:1000	Axon Medchem, #1484		
TGF-β3	1 μg/mL	1:1000	Peprotech, #100-36E		
dbcAMP	100 mM	1:200	Enzo, #BML-CN125-0100		

Table S4: Machine learning (ML) summary table as per data, optimization, model and evaluation (DOME) recommendations (Walsh et al., 2021).

**Data**	Provenance	Source of all used data where the experiments and image analysis described in this paper. **Table S5** contains information on all generated datasets.
	Data splits	Available data was split into 80% training data and 20% testing data. No separate validation set was used due to the use of k-fold cross validation.
	Redundancy between data splits	Train and test sets were generated using scikitlearn's train_test_split function with enabled stratification and random seeding to ensure that relative class frequencies were approximately preserved in each train and test set.
	Availability of data	All data is available in the **Supporting Material**. Data splits can be reproduced using the provided Jupyter Notebooks (link to GitHub in online version of paper).
**Optimization**	Algorithm	Established supervised binary classification algorithms were used. LDA was used for feature reduction and initial classification. Non-linear SVM proved to be more accurate and was subsequently used for classification tasks followed by leave-one-out (LOO) analysis to determine feature contributions. Tree-based LightGBM was used to confirm feature contributions determined by LOO.
	Meta-prediction	LDA was used during the feature selection process. See also "Features" section.
	Data encoding	Outliers were removed by applying a 3xSD window around each feature's median. Data was transformed on the same scale using the following formula: $(x_{Feature\ N} - median_{Feature\ N})/SD$.
	Parameters	SVM parameters were systematically identified using scikitlearn's GridSearchCV function. The parameters of all other models were determined empirically.<br>LDA: solver='eigen', n_components=1, shrinkage='auto'<br>SVM: probability=True, kernel='rbf', C=0.1-0.5 (depends on dataset, see provided Jupyter notebooks), gamma='scale'<br>LightGBM: boosting_type='goss', n_estimators=10000, class_weight = 'balanced'
	Features	**Table S5** contains information on all generated datasets including the total number of features. For feature reduction, Pearson correlation was used to exclude strongly correlated image features. For each dataset, LDA was used to determine a Pearson correlation cut-off value which maximized the accuracy of the classification and the Z factor between the two reference classes. The selected number and type of features are detailed per data set in the provided Jupyter notebooks.
	Fitting	Since the number of total features was high (>50) we used several approaches to prevent overfitting. GridSearchCV was used to optimize the SVM regularization parameter C. For LDA shrinkage was set to 'auto'. Additionally, we used k-fold cross validation to use a maximum of data for training and to prevent data leaking into the test set.
	Regularization	GridSearchCV was used to optimize the SVM regularization parameter C. The LDA parameter shrinkage was set to 'auto'.
	Availability of configuration	All configurations of all models for all data sets are reported in the provided Jupyter Notebooks (link to GitHub in online version of paper).
**Model**	Interpretability	Due to its non-linear nature the used SVM algorithm is not fully interpretable, but we performed LOO analysis to extract the approximate contribution of each used feature to the prediction.
	Output	SVM provides the classification probability to belong to one of the two used classes.
	Execution time	The entire workflow completes in ca. 80 seconds on a standard desktop computer.
	Availability of software	Jupyter Notebooks for each used data set are provided on GitHub. All dependencies are Python-based and freely available.
**Evaluation**	Evaluation method	25-fold cross-validation was used to validate the SVM and LDA models for all datasets.
	Performance measures	F1 score, recall and accuracy are reported.
	Comparison	We did not benchmark our models with previously existing data, since appropriate mDA neuron data is not available.
	Confidence	F1 score, recall and accuracy are reported together with confidence intervals.
	Availability of evaluation	Model evaluation can be reproduced using the Jupyter Notebooks for each used data set provided on GitHub.

Table S5: Overview of generated data sets for ML analysis.

Dataset	Figure in study	Cellular staining for image feature calculation	Dataset composition
1	Figure 3B-C	Hoechst/TH/aSyn/MAP2	N (imaged wells) = 504<br>N (total image features) = 126
2	Figure 3D	Hoechst/TH/aSyn/MAP2	N (imaged wells) = 502<br>N (total image features) = 126
3	Figure 3E	Hoechst/Calcein/TMRM	N (imaged wells) = 308<br>N (total image features) = 96
4	Figure 4D-E & Figure S5	Hoechst/TH/aSyn/MAP2	N (imaged wells) = 716<br>N (total image features) = 126
5	Figure 5C-E	Hoechst/TH/aSyn/MAP2	N (imaged wells) = 521<br>N (total image features) = 126
6	Figure S4	Hoechst/TH/aSyn/MAP2	N (imaged wells) = 560<br>N (total image features) = 126
7	Figure 6C-E	Hoechst/TH/aSyn/MAP2	N (imaged wells) = 1064<br>N (total image features) = 126
8	Figure 4F-G	Hoechst/LAMP2/aSyn/MAP2	N (imaged wells) = 196<br>N (total image features) = 57
9	Figure S6	Hoechst/TH/aSyn/MAP2	N (imaged wells) = 378<br>N (total image features) = 126

Supplemental experimental procedures

Western blotting
Samples were extracted from two differentiation batches of GS/+ and +/+ mDA neurons. Cell lysis was performed in RIPA buffer. Human cortex lysate served as control. Samples were loaded in NuPAGE LDS Sample Buffer (4x) (ThermoFisher, #NP0007) and ran on NUPAGE Novex 3-8% Tris-acetate gels (ThermoFisher, #EA03752BOX) immersed in NUPAGE Tris-acetate SDS running buffer (1x) (ThermoFisher, #LA0041) for 1 hour at 150V. Gels were transferred to PVDF membranes (Biorad, #1620177) in transfer buffer (25mM Tris, 192mM Glycine, 10% Methanol (pre-cooled)) overnight at 60V and room temperature. Membrane blocking was performed in 5% skim milk powder (ThermoFisher, #LP0031B) in TBS-T for 1 hour at room temperature. Used primary and secondary antibodies are summarized in **Table S3**. The membrane was developed using Super Signal® West Femto Maximum Sensitivity Substrate (ThermoFisher, #34095).

RT-qPCR
Samples were extracted from two differentiation batches of GS/+ and +/+ mDA neurons. Sample extraction was performed using a Maxwell 16 total RNA purification kit and the Maxwell RSC instrument. RNA concentration was determined using a NanoDrop ™. Quality control was performed using an Agilent 2100 Bioanalyzer. All samples had a RNA integrity number (RIN) value of 10. cDNA synthesis was performed using the qScript cDNA synthesis kit (Quantabio, #95047) on 500 ng RNA. The cDNA was diluted 1:5 in ddH_20 and GoTaq SYBR green 2x Super Mix (Promega) was used on a ViiA 7 Real-Time PCR System (ThermoFisher) using the primers indicated in Table S3.

Multi-electrode array (MEA)
Cryopreserved 30 DIV (days in vitro) old neurons were thawed in a water bath and centrifuged (400g, 5 min, RT) in basal medium (Table S3) supplemented with ROCK inhibitor (Tocris, #1254). Cell pellets were resuspended in differentiation medium (Table S3) supplemented with ROCK inhibitor. 48-well CytoView MEA plates (Axion Biosystems, #M768-tMEA-48W) were coated with 15 µg/ml Polyethyleneimine solution (Sigma, #P3143) for 1 hour at 37°C followed by 10µg/ml Laminin coating for 2 hours at 37°C. Using Tryphan Blue (Sigma, # T8154-20ML) and a Countess automated cell counter (Invitrogen), 80×10^3 cells/well were seeded in the CytoView plate. Cells were incubated at 37°C and 5% CO_2 for 31 days until 61 DIV with differentiation medium changes twice a week. Once a week, 1µg/ml Laminin was added to the fresh media to maintain cell attachment. Before each media change, the electrical activity was recorded on the Maestro Pro multiwell MEA (Axion Biosystems) using the AxIS Navigator software (version 3.5.1, Axion Biosystems). Electrical activity was recorded for 5 minutes at a 12.5 kHz sampling rate and a 5.5 standard deviation threshold level for action potential detection. Before plate loading the device was allowed to equilibrate for approximately 30 minutes to 37°C and 5% CO_2. Data analyses were performed using the Neural Metric Tool (version 3.1.7, Axion Biosystems). Activity is expressed as the mean firing rate across electrodes in a well. The activity represents the action potentials, defining the neuron function. Synchrony is expressed via the area under the well pooled inter-electrode cross-correlation normalized to the auto-correlations. Synchrony reflects the strength of synaptic connections, and thus how likely neurons are to generate action potentials simultaneously.

Dimensionality reduction
For dimensionality reduction we used the scikit-learn package's KernelPCA algorithm and PaCMAP (https://github.com/YingfanWang/PaCMAP). For PCA the following parameters were empirically chosen: n_components=2 and kernel='sigmoid'. For PaCMAP we performed a systematic parameter search and identified n_neighbors=25, MN_ratio=0.25, FP_ratio=0.5, and num_iters=5000 as ideal parameters to resolve the data structure. The raw data can be found in the Supplemental Information and the pipeline used for dimensionality reduction is available as a Jupyter notebook on GitHub (https://github.com/johanneswilbertz/mDA-neuron-classification).
